# Engineered Nonviral Protein Cages Modified for MR Imaging

**DOI:** 10.1021/acsabm.2c00892

**Published:** 2023-01-10

**Authors:** Megan A. Kaster, Mikail D. Levasseur, Thomas G. W. Edwardson, Michael A. Caldwell, Daniela Hofmann, Giulia Licciardi, Giacomo Parigi, Claudio Luchinat, Donald Hilvert, Thomas J. Meade

**Affiliations:** † Departments of Chemistry, Molecular Biosciences, Neurobiology and Radiology, Northwestern University, 2145 N. Sheridan Road, Evanston, Illinois60208, United States; ‡ Laboratory of Organic Chemistry, ETH Zurich, Vladimir-Prelog-Weg 1-5/10, Zürich8093, Switzerland; § Magnetic Resonance Center (CERM), University of Florence, via Luigi Sacconi 6, Sesto Fiorentino50019Italy; ∥ Department of Chemistry “Ugo Schiff”, University of Florence, via della Lastruccia 3, Sesto Fiorentino50019, Italy; ⊥ Consorzio Interuniversitario Risonanze Magnetiche Metallo Proteine (CIRMMP), via Luigi Sacconi 6, Sesto Fiorentino50019, Italy

**Keywords:** nonviral protein cages, magnetic resonance imaging, gadolinium, magnetism, NMRD

## Abstract

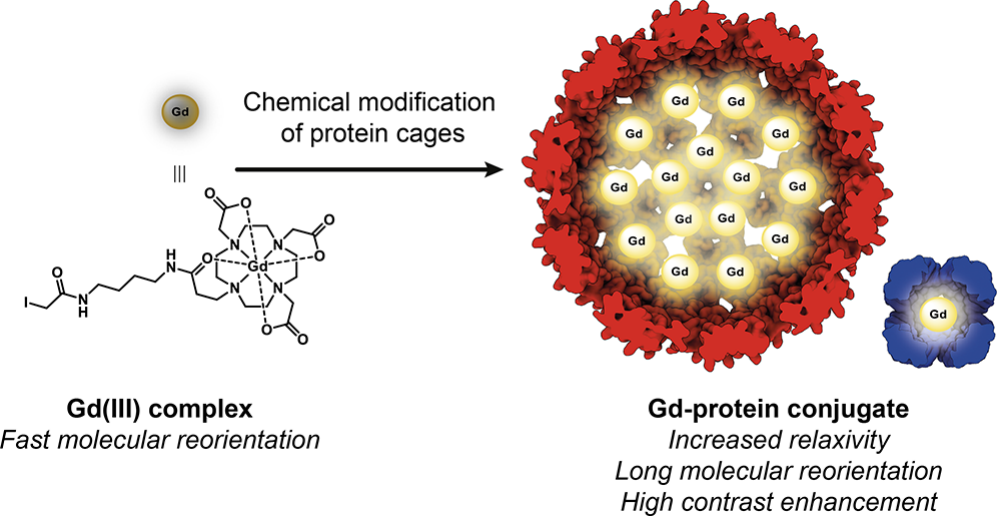

Diagnostic medical imaging utilizes magnetic resonance (MR) to provide anatomical, functional, and molecular information in a single scan. Nanoparticles are often labeled with Gd(III) complexes to amplify the MR signal of contrast agents (CAs) with large payloads and high proton relaxation efficiencies (relaxivity, *r*_1_). This study examined the MR performance of two structurally unique cages, AaLS-13 and OP, labeled with Gd(III). The cages have characteristics relevant for the development of theranostic platforms, including (i) well-defined structure, symmetry, and size; (ii) the amenability to extensive engineering; (iii) the adjustable loading of therapeutically relevant cargo molecules; (iv) high physical stability; and (v) facile manufacturing by microbial fermentation. The resulting conjugates showed significantly enhanced proton relaxivity (*r*_1_ = 11–18 mM^–1^ s^–1^ at 1.4 T) compared to the Gd(III) complex alone (*r*_1_ = 4 mM^–1^ s^–1^). Serum phantom images revealed 107% and 57% contrast enhancements for Gd(III)-labeled AaLS-13 and OP cages, respectively. Moreover, proton nuclear magnetic relaxation dispersion (^1^H NMRD) profiles showed maximum relaxivity values of 50 mM^–1^ s^–1^. Best-fit analyses of the ^1^H NMRD profiles attributed the high relaxivity of the Gd(III)-labeled cages to the slow molecular tumbling of the conjugates and restricted local motion of the conjugated Gd(III) complex.

## 1 Introduction

Magnetic resonance (MR) imaging is an attractive modality for medical diagnostic imaging because of its unlimited depth penetration, excellent spatiotemporal resolution, and safety profile that does not require ionizing radiation or radiotracers. Furthermore, MR provides unparalleled native soft tissue contrast with highly detailed anatomical information based on inherent tissue differences arising from proton density, perfusion and diffusion, and biomolecule content.^1,2^ Administration of contrast agents (CAs) with paramagnetic metal ions greatly enhances tissue contrast by shortening the longitudinal (*T*_1_) and transverse (*T*_2_) relaxation times of protons on local water molecules. In addition, CAs can be designed to report on biomarkers for molecular imaging applications, enabling the correlation of molecular information with tissue structures in a single scan.

Clinical MR imaging often utilizes trivalent gadolinium (Gd(III)) based CAs for *T*_1_-weighted images, where short *T*_1_ values correspond to a bright MR signal.^3^ The efficiency with which a CA influences the water proton *T*_1_ is the relaxivity (*r*_1_), defined by eq 1. The observed longitudinal relaxation rate constant in the presence of CA (1/*T*_1_^obs^) comprises a background diamagnetic component (*T*_1_^o^) and a paramagnetic component consisting of *r*_1_ and the concentration of Gd(III) in the CA ([Gd(III)]).
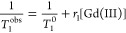
1

Clinically approved small molecule Gd(III) CAs typically have *r*_1_ ∼ 4 mM^–1^ s^–1^ at clinical MR field strengths (0.2–3 T), where a local concentration of over 100 μM CA is required to induce a detectable change in *T*_1_ over a biological background.^4−6^ However, it is difficult for small molecule CAs to reach these local concentrations through biomarker targeting because most biomarkers of interest are expressed at micromolar to picomolar concentrations.^7^ Thus, signal amplification strategies are needed to bridge the gap between biomarker expression and the MR detection limit.

Nanoparticles (NPs) provide an attractive platform for MR imaging with several advantages over their small molecule counterparts, as they can (1) carry a high Gd(III) payload; (2) improve relaxivity per Gd(III) at clinically relevant field strengths; (3) incorporate targeting groups and therapeutic cargo; and (4) extend circulation lifetimes, offering a means to tune biodistribution. The first two points are signal amplification strategies that increase the paramagnetic contribution to the observed MR signal (eq 1). The second, increasing relaxivity, also enhances the safety profile by requiring lower dosing concentrations of Gd(III).^8^ Concerns about toxicity related to Gd(III)-based CAs, such as nephrogenic systemic fibrosis (NSF) and Gd(III) deposition in organs, arise primarily from free Gd(III) ions that dissociate from their ligand due to poor kinetic stability. Thus, kinetically stable Gd(III) complexes with macrocyclic ligands have been used clinically in over 500 million MR scans worldwide with only 1 severe adverse event per 40,000 injections.^3,8^ Gd(III)-labeled NPs further mitigate toxicity issues by having lower dosing concentrations than required for molecular Gd(III) complexes.

Several types of NPs have been investigated for MR signal amplification as Gd(III)-labeled conjugates,^9^ including gold NPs,^10−17^ carbon nanodiamonds,^18^ metal–organic frameworks,^19^ liposomes,^20,21^ dendrimers,^22−28^ polymers,^29−31^ micelles,^32−35^ and hydrogels.^36,37^ Of the parameters that govern *r*_1_ (eqs 1 and 2), conjugating Gd(III) complexes to NPs most strongly influences the rotational correlation time (τ_R_, eq 3) to achieve what is called a “*τ*_R_ boost” in relaxivity at clinical field strengths. A brief discussion of MR physics is required to understand the origin of this *τ*_R_ boost.

The relaxation processes of nuclear spins (i.e., *T*_1_ of water protons) can be enhanced through magnetic dipole–dipole interactions with unpaired electrons in paramagnetic metals. The strength of this interaction depends on the spin of the paramagnetic metal, the distance between nuclear and electronic spins, and the precession frequencies of nuclear and electronic spins. The high spin state (S = 7/2) and long electronic relaxation time (*T*_1e_ ∼ 10^–9^ s at clinical field strengths) of Gd(III) make it an excellent candidate to influence water protons for MR imaging. The highest contribution to *r*_1_ comes from water molecules directly coordinated to Gd(III) with a mean residence time *τ*_m_ and exchange with bulk water (eq 2). While *r*_1_ is directly proportional to the number of water molecules bound to Gd(III) (*q*), increasing *q* can lead to poor kinetic ligand stability, which would lead to the release of Gd(III) ions from the ligand.^4^

The dipolar longitudinal relaxation time (*T*_1m_) originates from the modulation of the magnetic dipole–dipole interaction between electron and proton spins. The time constant of this modulation known as the rotational correlation time (*τ*_c_), is determined by the fastest parameter among *T*_1e_, *τ*_R_, and *τ*_m_ (eq 3). Relaxivity approaches the theoretical maximum value when the inverse correlation time (*τ*_c_^–1^) matches the Larmor frequency (*ω*_I_) of water protons. This is when the coupling of the electronic and nuclear spins is most efficient. At clinically relevant field strengths, the fast *τ*_R_ values (10^–10^ s to 10^–12^ s) of small molecule Gd(III) CAs determine *τ*_c_ and limit relaxivity. Tethering Gd(III) complexes to macromolecules results in a slower *τ*_R_ that does not determine *τ*_c_, allowing relaxivity to approach theoretical maximum values (the *τ*_R_ boost).
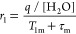
2
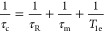
3

The extended circulation lifetimes of NPs and their amenability to modification also make them attractive cargo delivery vehicles. Many NPs can also serve as theranostic agents, carrying both diagnostic agents and therapeutic drugs, to monitor the drug’s efficacy noninvasively and in real time.^38−41^ NPs can be further modified to control their biodistribution and for targeted delivery of a therapeutic drug or molecular imaging of a biomarker, depending on the nature of the cargo. Finally, the extended circulation lifetimes of Gd(III)-labeled NPs compared to small molecule Gd(III) complexes increases bioavailability and potential for cellular uptake.^3,4,42^ Although NPs provide many benefits for Gd(III)-based MR CAs, concerns with the safety profile of synthetic and inorganic NPs have motivated the search for platforms made from biocompatible materials.

Nanoscale compartments formed by self-assembling proteins are a promising class of NP for achieving MR signal amplification. These protein cages are biodegradable and readily produced recombinantly from bacterial or mammalian cell cultures. Furthermore, the cages assemble with high efficiency and fidelity into monodisperse particles amenable to characterization at the molecular scale not afforded by many other NP materials.^43^ Protein cages have been investigated as high-relaxivity agents due to their diversity in terms of shape, size, valency, and the ability to modify both the exterior and interior surfaces.^43^ These high relaxivity agents can be broadly grouped into three main categories based on design: (1) binding of Gd(III) ion at endogenous^44^ or genetically engineered^45,46^ metal binding sites, (2) noncovalent loading of Gd(III) complexes as cargo, and (3) covalent conjugation of Gd(III) complexes to protein cages.^45,47−58^ The third approach is attractive for developing theranostic platforms as it allows the cage to be optimized for therapeutic cargo while maintaining an ability to covalently bind Gd(III) complexes.

**Figure 1:**
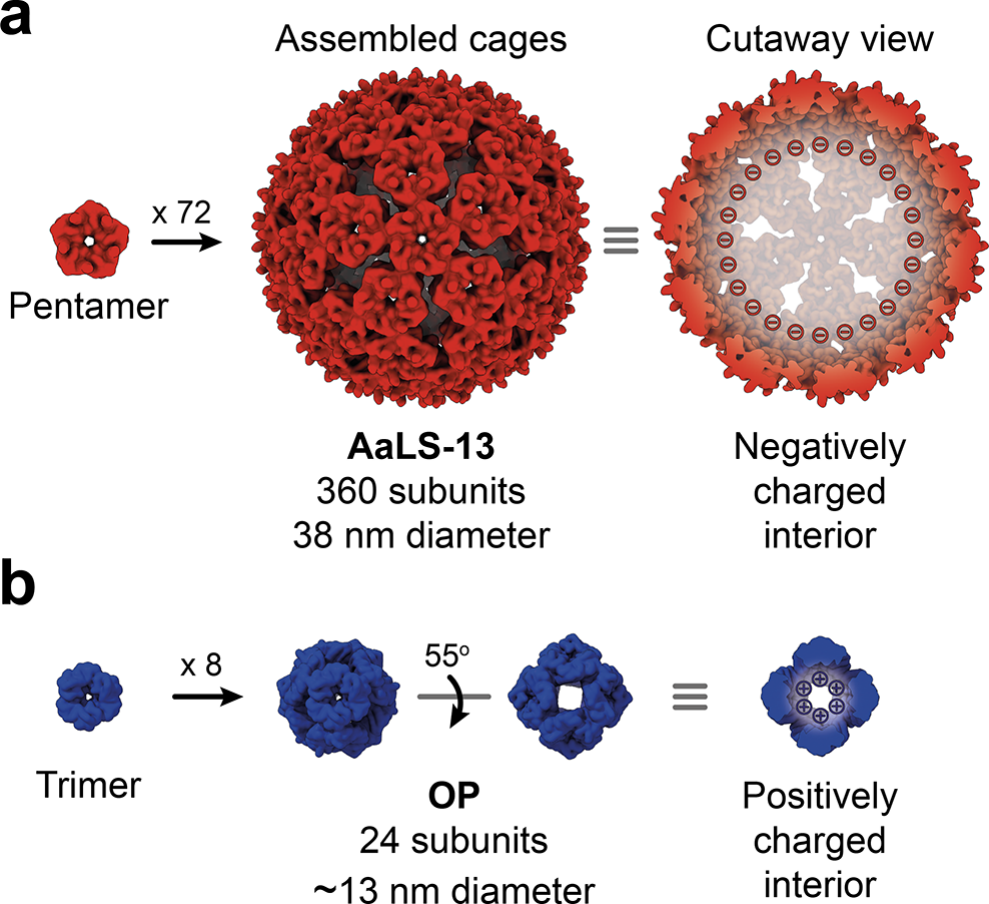
Engineered AaLS-13 and OP protein cages. Surface representation of (a) AaLS-13 (PDB 5MQ7) and (b) OP (PDB 6FDB) cages. AaLS-13 assembles into 38 nm spherical cages, possessing a negatively supercharged interior, from 72 pentameric subunits. OP forms ∼13 nm positively supercharged cubic cages from eight trimeric capsomers.

Two structurally distinct engineered protein cages, AaLS-13 and OP, are excellent candidates for the development of NP platforms for MR imaging. AaLS-13 is an evolved variant of the cage-forming enzyme lumazine synthase from *Aquifex aeolicus.* AaLS-13 self-assembles from 360 monomer proteins into 38 nm icosahedrally symmetric cages (Figure 1a).^59−61^ Owing to its negatively supercharged interior and large keyhole-shaped surface pores, AaLS-13 encapsulates positively charged cargo at rates approaching the diffusion limit.^62,63^ Additionally, the surface-exposed termini of AaLS-13 offer further functionalization opportunities, which have already been exploited to display antibodies^64^ or for enzymatic labeling.^65−67^

OP, in contrast, is a smaller (∼13 nm diameter), 24-subunit, octahedrally symmetric cage, which derives from the computationally designed O3–33 cage (Figure 1b).^68^ Positive charges were introduced by mutating six lumenal residues in the starting scaffold to arginine, enabling efficient encapsulation of negatively charged cargo, such as oligonucleotides^69^ and anionic surfactants.^70^ Therapeutically relevant guests, like siRNA and drug-loaded micelles, have been successfully delivered to cells using OP, substantially improving the potency of the active ingredients. These properties make it a promising molecular delivery vehicle.^71^

AaLS-13 and OP cages have been applied as delivery vehicles for proteins, oligonucleotides, and small molecules. Conjugating DOTA-based Gd(III) complexes to these tunable cages provides an opportunity to develop nonviral theranostic platforms. To this end, we have covalently linked Gd(III) complexes to the interior and exterior of AaLS-13 and OP cages and assessed how these modifications influence the MR signal. Relaxivity measurements of Gd(III)-labeled proteins show substantial signal amplification with high Gd(III) payloads per cage as well as high relaxivity. In addition to the τ_R_ boost, the highly charged cage interior appears to restrict Gd(III) complex mobility. Notably, because of the significant signal amplification, these Gd(III)-protein conjugates provide detectable contrast enhancement at concentrations below those of common small molecule Gd(III) complexes used in the clinic.

## 2 Results and Discussion

### 2.1 Synthesis and Characterization of Gd-C4-IA

**Figure 2:**
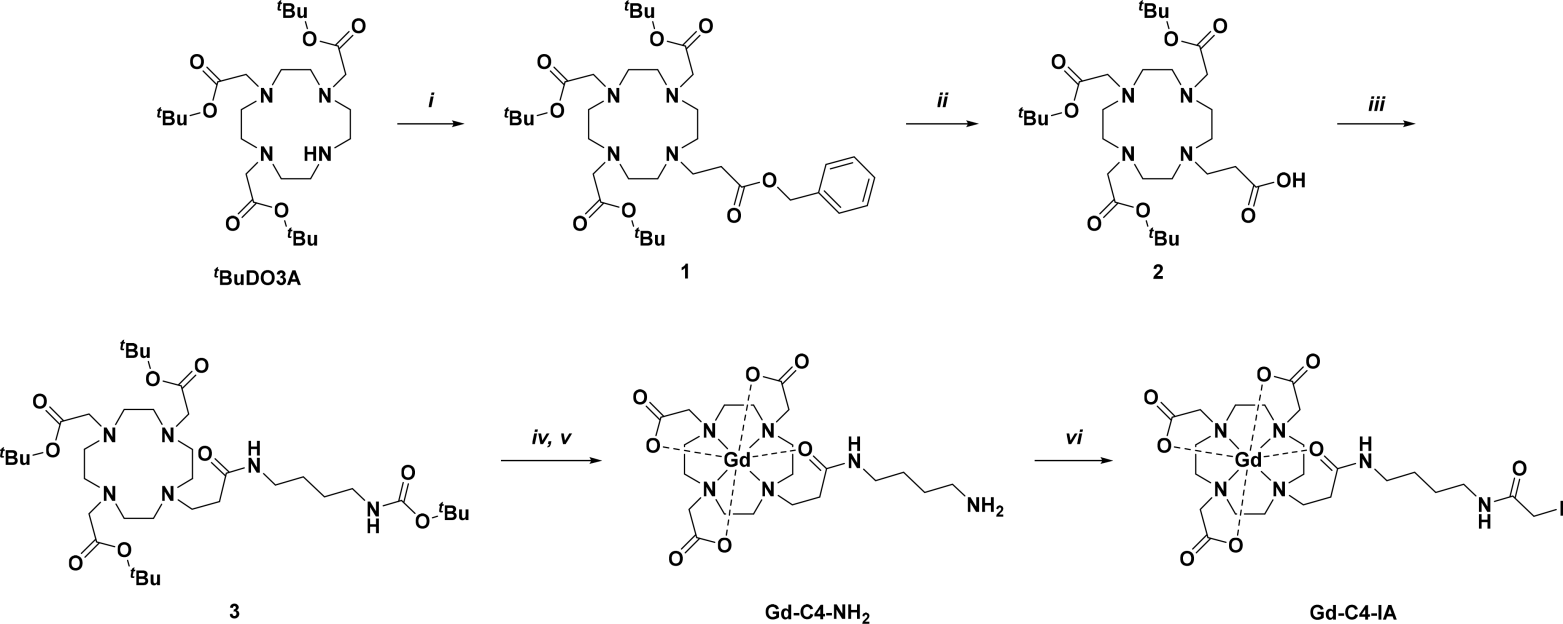
Synthetic scheme for Gd-C4-IA. (*i*) ^*t*^BuDO3A (1 equiv), benzyl acrylate (2 equiv), DIEA (5.9 equiv), MeCN, N_2_ (g), RT, 47%; (*ii*) **1** (1 equiv), Pd/C (catalyst), MeOH, H_2_ (g), RT, 27%; (*iii*) **2** (1 equiv), ^*t*^Bu (4-aminobutyl)carbamate (1.5 equiv), NHS (3 equiv), DIEA (5 equiv), DIC (5 equiv), DMF, N_2_ (g), RT, quantitative yield; (*iv*) **3** (1 equiv), TFA, CH_2_Cl_2_, N_2_ (g), RT, crude; (*v*) **4** (1 equiv), GdCl_3_·6H_2_O (1.3 equiv), H_2_O, N_2_ (g), RT, 34% over 2 steps; (*vi*) Gd-C4–NH_2_ (1 equiv), iodoacetic anhydride (3 equiv), K_2_CO_3_ (3 equiv), DMF, N_2_ (g), 0 °C, 20%.

The Gd(III) complex Gd-C4-IA was designed using the macrocyclic cyclen scaffold of clinically approved CAs that exhibits good kinetic stability. Gd-C4-IA was synthesized as described in Figure 2, with characterization data for compounds **1**−**4** (Scheme S1) provided in Figures S1−S23. Literature conditions were followed to prepare tri-^*t*^Bu 2,2′,2″-(1,4,7,10-tetraazacyclododecane-1,4,7-triyl)triacetate (^*t*^BuDO3A) and benzyl acrylate.^51,72,73^ Benzyl acrylate served as the foundation for the functional arm that was later used to covalently link Gd-C4-IA to the protein cages. ^*t*^BuDO3A and benzyl acrylate were reacted via an aza-Michael addition. The functional arm was deprotected by Pd/C hydrogenation and reacted with ^*t*^Bu-(4-aminobutyl)carbamate. Acidic conditions were used to perform a global deprotection of all ^*t*^Bu groups, followed by metalation with GdCl_3_. The Gd-C4-NH_2_ intermediate was purified by high-performance liquid chromatography (HPLC) (Figure S24a), and then reacted with iodoacetic anhydride to give Gd-C4-IA, which was purified by HPLC (Figure S24b).

### 2.2 Preparation of Protein Variants

**Figure 3:**
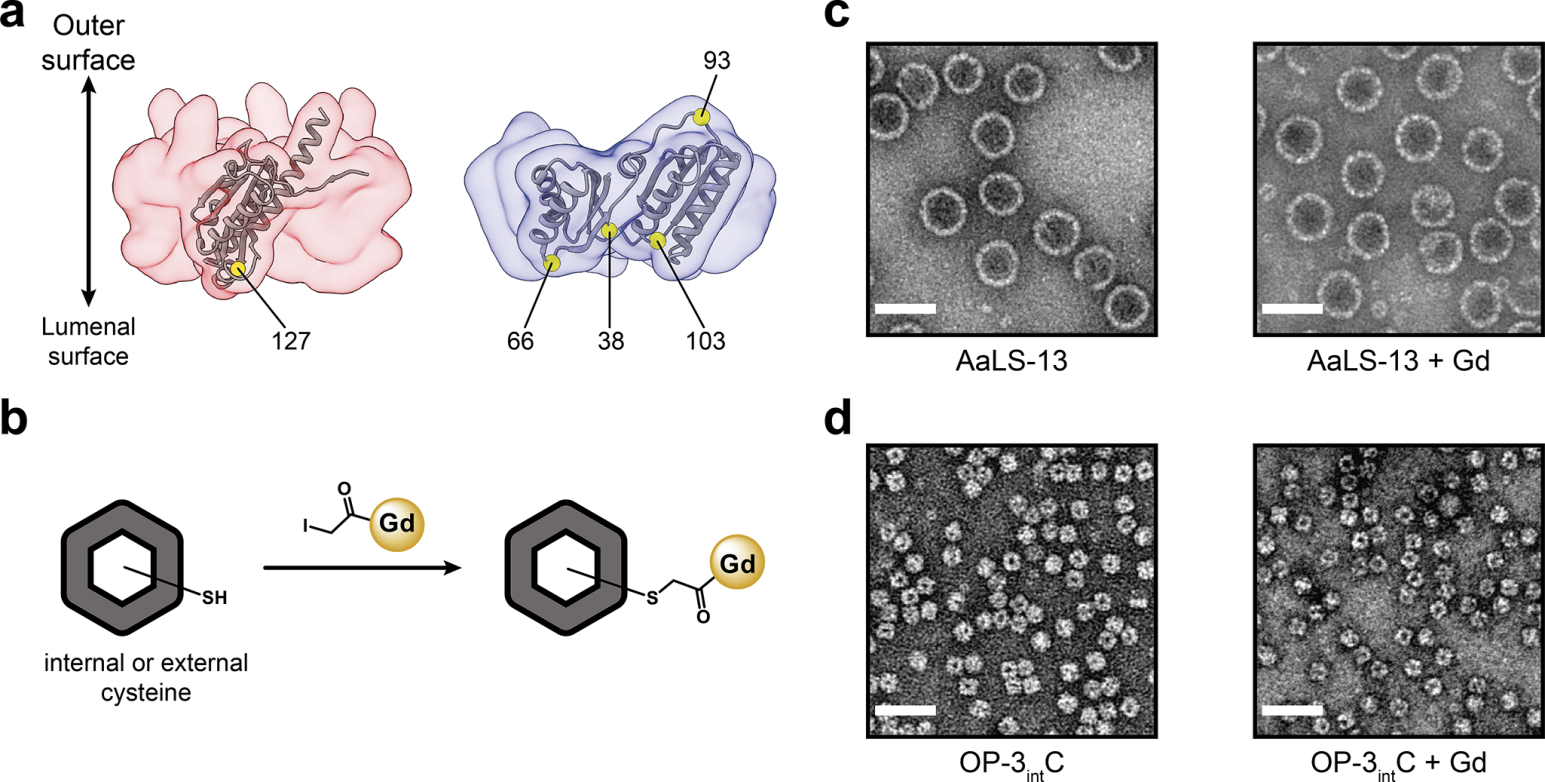
Thiol-mediated functionalization of AaLS-13 and OP and transmission electron microscopy (TEM) images of AaLS-13 and OP-3_int_C. (a) Transparent surface of a pentamer used to construct an AaLS-13 cage (left) and a trimer used to construct OP (right). Monomers are shown as gray ribbons. Targeted cysteine residue on AaLS-13 (Cys127) and positions on OP targeted for cysteine mutations (Ser38, Arg66, and Arg103) are highlighted as yellow spheres. (b) Representations of Gd(III) labeling the cysteine reactive sites. TEM images of (c) AaLS-13 and (d) OP-3_int_C cages, unmodified (left) or labeled with Gd-complexes (right). Scale bar is equal to 50 nm.

Wild-type (wt) AaLS possesses one buried cysteine (Cys37) per monomer with negligible reactivity.^62^ Functionalization of wt AaLS using thiol-reactive species requires introducing additional surface exposed cysteine residues.^74^ In contrast, AaLS-13 contains two additional cysteine residues (Cys52 and Cys127) per monomer that were introduced during evolution. Although Cys52 is buried, Cys127 is located on the lumenal surface and can be exploited for thiol-mediated labeling (Figure 3a, left).

OP possesses two cysteines per monomer (Cys108 and Cys136) that form a buried disulfide. To provide specific reactive handles for Gd-C4-IA conjugation, cysteine mutations were introduced at surface exposed, inner loop positions that were previously shown to be mutable.^69^ The three variants, OP-1_int_C (S38C), OP-2_int_C (S38C, R103C), and OP-3_int_C (S38C, R66C, R103C), provide 24, 48, or 72 reactive sites per multimeric assembly on the lumenal surface, respectively. An additional variant, OP-1_ext_C, which presents 24 cysteine residues on the exterior surface of each cage, was designed by mutating the surface exposed lysine at position 93 to cysteine (K93C) (Figure 3a, right).

### 2.3 Conjugation of Gd(III) Complex with Protein Cages

The Gd-C4-IA complex was conjugated to the protein cages by alkylation of the cysteine thiols in the protein cages with the iodoacetamide group in Gd-C4-IA (Figure 3b). Modification was performed by mixing AaLS-13 or the OP cages with 2 equiv of Gd-C4-IA per reactive thiol and incubating for 4.5 h at room temperature in the dark. Unreacted Gd-C4-IA was removed through desalting columns and the Gd-C4-protein conjugates were isolated by size exclusion chromatography (SEC, Figure S27). The AaLS-13 and OP cages remain intact after labeling with Gd-C4-IA (Figure 3c,d).

The concentration of Gd-C4-IA and protein in the purified samples was determined by ICP-MS and UV–vis measurements, respectively (Table S1, Figure S30). Labeling efficiency, defined as the number of reactive sites per cage successfully labeled with Gd-C4-IA, was measured to evaluate the ability of the cages to carry a high payload of Gd(III) complex for MR imaging (Table 1).

**Table 1:** Labeling of Protein Cages with Gd(III) Complexes

sample name	labeling^a^(complexes/cage)	reactive sites	labeling efficiency (%)
Gd-AaLS-13	149 ± 12	360	41
Gd-OP-3_int_C	33 ± 7	72	46
Gd-OP-2_int_C	23 ± 1	48	47
Gd-OP-1_int_C	11 ± 1	24	46
Gd-OP-1_ext_C	14 ± 2	24	60

a Standard deviation accounts for variations across biological replicates.

The porous nature of the cages enables efficient labeling of reactive sites positioned on the lumenal surface (41–47% for AaLS-13, OP-3_int_C, OP-2_int_C, and OP-1_int_C). Labeling efficiency is even higher for reactive sites positioned on the exterior surface (60% for OP-1_ext_C). These labeling efficiencies are in the range of previously reported values.^62,74^ Furthermore, the Gd(III)-labeled cages were shown to be stable for several months by MS analysis (Figures S28, S29).

### 2.4 MR Signal Amplification Revealed through Relaxivity Measurements

Relaxivity measurements for Gd-C4-IA and the Gd-C4-protein conjugates were performed at a clinically relevant low field strength (1.4 T, Figure S31) and at a higher field strength (7 T, Figures S31−S38) used for high-resolution imaging. The results are reported in Table 2 and Table S2. Increasing the field strength of MR instruments improves the signal-to-noise ratio (SNR) and spatial resolution, and also shortens acquisition times.^75,76^ The ionic relaxivity (*r*_1,ionic_) values are normalized per millimolar Gd(III) to identify the structure that imparts the best MR physics properties, whereas the particle relaxivity (*r*_1,particle_, Eq. S1) accounts for the number of Gd(III) complexes per particle as well as *r*_1,ionic_.

**Table 2:** Relaxivity Measurements of Gd-C4-IA and Gd(III)-Labeled Protein Cages at 1.4 and 7 T^a^

	1.4 T at 37 °C		7 T at 25 °C	
sample name	*r*_1,ionic_(mM^–1^ s^–1^)	*r*_1,particle_(mM^–1^ s^–1^)	*r*_1,ionic_(mM^–1^ s^–1^)	*r*_1,particle_(mM^–1^ s^–1^)
Gd-C4-IA^b^	4.2	N/A	2.6	N/A
Gd-C4-IA^c^	4.1	N/A	3.2	N/A
Gd-AaLS-13^b^	18.3	2727	8.0	1192
Gd-OP-3_int_C^c^	15.9	525	5.3	175
Gd-OP-2_int_C^c^	18.0	419	5.4	124
Gd-OP-1_int_C^c^	15.0	165	4.6	51
Gd-OP-1_ext_C^c^	11.2	157	4.9	69

a Relaxation times (*T*_1_) were measured with error of <5%, while standard deviations of [Gd(III)] were determined by ICP-MS of triplicate samples.

b Relaxivity data in 50 mM sodium phosphate (pH 8.0), 200 mM NaCl, 5 mM EDTA.

c Relaxivity data in 25 mM Tris (pH 7.6), 200 mM NaCl, 5 mM EDTA.

Gd-C4-IA was studied in sodium phosphate buffer (50 mM sodium phosphate (pH 8.0), 200 mM NaCl, 5 mM EDTA) and Tris buffer (25 mM Tris (pH 7.6), 200 mM NaCl, 5 mM EDTA). The ionic relaxivity of Gd-C4-IA behaved as expected for a small molecule Gd(III) complex, with values of 4.1–4.2 mM^–1^ s^–1^ at 1.4 T and 37 °C, and decreased to 2.6 and 3.2 mM^–1^ s^–1^ at 7 T and 25 °C (Table 2). These values are consistent those observed for *q* = 1 Gd(III) complexes.

As expected, higher *r*_1,ionic_ values were measured for the Gd-C4-protein conjugates compared to Gd-C4-IA at both field strengths due to the τ_R_ boost (Table 2), with a stronger *τ*_R_ boost observed at the lower field strength (1.4 T, 37 °C). For Gd-AaLS-13, *r*_1,ionic_ increased from ∼4 to 18.3 mM^–1^ s^–1^, while conjugation to the OP cages increased *r*_1,ionic_ to 11–18 mM^–1^ s^–1^. At 7 T and 25 °C, a smaller *τ*_R_ boost was also observed, with *r*_1,ionic_ increasing from ∼3 mM^–1^ s^–1^ to 5–8 mM^–1^ s^–1^ for the Gd(III)-C4-protein conjugates. Interestingly, the protein cages labeled with Gd(III) on the lumenal surface (Gd-AaLS-13, Gd-OP-3_int_C, Gd-OP-2_int_C, and Gd-OP-1_int_C) showed higher *r*_1,ionic_ values than Gd-OP-1_ext_C with Gd(III) labeled on the external surface at the low field strength conditions (1.4 T and 37 °C). This trend is also observed under the high field strength conditions (7 T and 25 °C), except for Gd-OP-1_int_C, which shows an *r*_1,ionic_ lower than but comparable to that of Gd-OP-1_ext_C.

The ionic relaxivities measured here are comparable to other Gd(III)-labeled protein cages, which show relaxivities of 10–60 mM^–1^ s^–1^, depending on experimental conditions, particle size, and location of the Gd(III) complex.^47,48,50−55,57,58^ Notably, previous studies of Gd(III)-labeled wt AaLS cages showed ionic relaxivities of 30–60 mM^–1^ s^–1^ at 1.4 T and 37 °C and 16 mM^–1^ s^–1^ at 7 T and 25 °C.^52,58^ Gd(III) complexes in the interior of MS viral capsids similarly had higher ionic relaxivities than Gd(III) complexes on the cage exterior,^48^ likely due to the restricted flexibility of Gd(III) complexes at the cage interior compared to their counterparts at the exterior surface.^49^

In order to determine the cause of the differences in *r*_1,ionic_ between the small molecule Gd-C4-IA and the Gd-C4-protein conjugates, and among the Gd(III)-labeled cages, the relaxation mechanisms were investigated with nuclear magnetic relaxation dispersion (NMRD) profiles.

### 2.5 Relaxation Mechanistic Details Obtained from ^1^H Nuclear Magnetic Relaxation Dispersion Profiles

#### General Description of Methodology

^1^H NMRD profiles are routinely used to study relaxation mechanisms of paramagnetic complexes and nanomaterials.^78^ This technique measures the relaxation rate constants of water protons across a range of magnetic field strengths (0.0002–1 T). Fitting NMRD data to relaxation theory models reveals mechanistic information about paramagnetic complexes.^78^ The low field portion of the NMRD profiles (0.0001–0.1 T) are fit using a modified Florence NMRD program,^79−81^ which accounts for the presence of static zero-field splitting (ZFS) of Gd(III) which primarily affects low field relaxivity. The high field region of the profile (0.1–1 T) is not affected by static ZFS and can thus be interpreted using the so-called SBM model requiring fewer parameters.^82^

**Figure 4:**
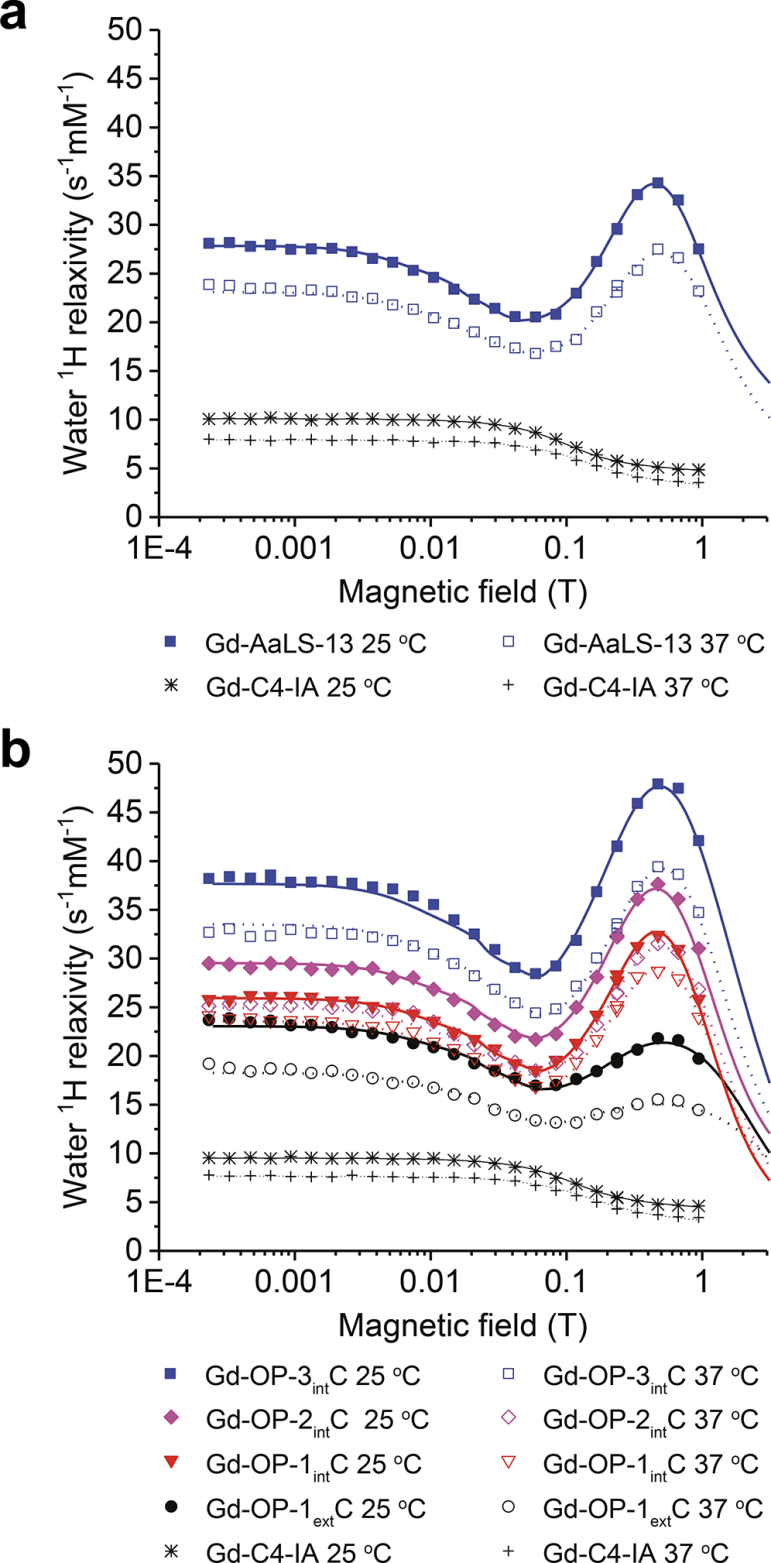
^1^H relaxivity profiles of Gd-C4-IA and Gd(III)-labeled protein cages. (a) Gd-C4-IA and Gd-AaLS-13 in sodium phosphate (pH 8.0) buffer at 25 °C and 37 °C. (b) Gd-C4-IA, Gd-OP-3_int_C, Gd-OP-2_int_C, Gd-OP-1_int_C, and Gd-OP-1_ext_C in Tris (pH 7.6) buffer at 25 °C and 37 °C. Solid and dotted lines are the best fit profiles at 25 and 37 °C, respectively, obtained with the parameters reported in Table 3 and Tables S4–S6.

Water ^1^H NMRD profiles for Gd-C4-IA and the Gd-C4-protein conjugates were collected at 25 and 37 °C in sodium phosphate (Gd-C4-IA and Gd-AaLS-13) and Tris (Gd-C4-IA and OP) buffers, and the normalized relaxivities per millimolar Gd(III) (*r*_1,ionic_) are shown in Figure 4. These best fit profiles (Figure 4, solid versus dotted lines) were obtained using the parameters reported in Table 5 and Tables S4–S6.

#### ***τ***_R_ Boost of the Gd-C4-Protein Conjugates Yields High Relaxivity

The profiles of Gd-C4-IA in both buffers look as expected for a small molecule Gd(III) complex with *q* = 1 (Figure 4).^78^ The relaxivities of the Gd(III)-labeled protein cages are much larger than that of Gd-C4-IA, showcasing successful signal amplification with a maximum *r*_1,ionic_ of 20–50 mM^–1^ s^–1^ (Table 3). In the case of the Gd(III)-labeled OP cages, the ionic relaxivities of cages with Gd(III) on the lumenal surface (Gd-OP-3_int_C, Gd-OP-2_int_C, and Gd-OP-1_int_C) are higher than cages with Gd(III) on the external surface (Gd-OP-1_ext_C) (Figure 4b). Furthermore, the ionic relaxivity of the lumenally labeled cages progressively increases with increasing number of Gd(III) complexes per cage from Gd-OP-1_int_C to Gd-OP-2_int_C to Gd- OP-3_int_C. These results are consistent with the ionic relaxivity at 1.4 and 7 T (Table 2).

**Table 3:** Maximum Ionic Relaxivity for Gd-C4-IA and the Gd-C4-Protein Conjugates

sample name	Max *r*_1,ionic_ at 25 °C	Max *r*_1,ionic_ at 37 °C
Gd-C4-IA^a^^,^^c^	10	7.5
Gd-C4-IA^b^^,^^c^	10	7.5
Gd-AaLS-13^a^^,^^d^	35	27.5
Gd-OP-3_int_C^b^^,^^d^	48	40
Gd-OP-2_int_C^b^^,^^d^	37.5	30
Gd-OP-1_int_C^b^^,^^d^	32.5	28
Gd-OP-1_ext_C^b^^,^^d^	20	15

a Relaxivity data in 50 mM sodium phosphate (pH 8.0), 200 mM NaCl, 5 mM EDTA.

b Relaxivity data in 25 mM Tris (pH 7.6), 200 mM NaCl, 5 mM EDTA.

c Relaxivity values at <0.02 T.

d Relaxivity values at 0.5 T.

The shapes of the Gd(III)-labeled cage profiles (Figure 4) are relatively similar, with the appearance of peaks in the high field region (∼0.5 T) indicating a field dependence of *τ*_c_ that originates from the field dependence of *T*_1e_. Thus, the other field independent parameters in eq 3 (*τ*_R_ and *τ*_m_) must be longer than *T*_1*e*_. On the other hand, the absence of this peak for the Gd-C4-IA profiles (Figure 4) demonstrates that relaxivity of the Gd(III) complex is limited by a *τ*_c_ determined by a fast *τ*_R_.

Very few Gd(III)-labeled protein cages have been studied by ^1^H NMRD, including Gd(III)-polymer covalently attached to the interior of protein cages^50,55^ and Gd(III)-labeled MS2 viral capsids.^49^ The Gd(III)-labeled MS2 system is comparable to Gd(III)-labeled AaLS-13 and OP cages studied here, with a peak appearing in the high field region (∼0.7 T) with maximum relaxivity values of 30–40 mM^–1^ s^–1^.^49^

#### Water Exchange Rate Does Not Limit the *τ*_R_ Boost

The profiles in Figure 4 show a significant decrease in ionic relaxivity with increasing temperature across the whole range of field strengths. This temperature dependence indicates that *τ*_m_ < *T*_1m_ (eq 2), i.e., that the coordinated water molecule is in the fast exchange regime. Under these conditions, the water lifetime (*τ*_m_) is in the range of 10^–8^ to 10^–7^ s (Tables S5 and S6) and does not limit the correlation time (*τ*_m_ > *T*_1e_). Gd(III)-labeled MS2 capsids have also been reported to possess water molecules in the fast exchange regime, observed by the temperature dependence of ^1^H NMRD profiles.^43^ A long *τ*_R_, and a lifetime *τ*_m_ longer than *T*_1e_ but shorter than *T*_1m_ (*τ*_R_*> T*_1m_*> τ*_m_*> T*_1e_) represent ideal conditions for maximizing relaxivity.

#### Two Correlation Times Contribute to the Relaxation Mechanism

The best fit analysis of the profiles indicates that two different correlation times must contribute to the modulation of the dipole–dipole interaction between Gd(III) and water proton spins for all Gd(III)-labeled protein cages. These two correlation times are modeled using the Lipari-Szabo model-free approach,^83^ with an *S*^2^ parameter providing the weight of the slower correlation time (*τ*_c1_ from eq 4 and 1 – *S*^2^ as the weight for the faster correlation time (*τ*_c2_ from eq 5), where *τ*_l_ is the correlation time of the faster local mobility (Table 4).
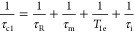
4
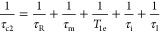
5In both buffers, Gd-C4-IA exhibits *τ*_R_ values of tens of picoseconds (Table S4), as expected for a fast-tumbling small molecule.^78,84^ On the other hand, both Gd(III)-labeled AaLS-13 and OP proteins show similar high-field *τ*_c1_ values on the nanosecond time scale, despite their different sizes (Table 4). These values are orders of magnitude smaller than the overall tumbling times of the protein cages (*τ*_R_) as indicated by the relaxation profiles of the diamagnetic proteins (Table S3, Figure S39), and are also smaller than *τ*_m_ (10^–8^ to 10^–7^ s, Tables S5 and S6). Thus, this time value should be determined by an intermediate correlation time (*τ*_i_) that is slower than the fast local mobility correlation time (*τ*_l_) and faster than the global correlation time (*τ*_R_). The *τ*_l_ values on the picosecond to nanosecond time scale (Table 4) suggest that considerable flexibility of the Gd(III) tag allows for extensive reorientation of the Gd(III) complex.

**Table 4:** Selected ^1^H NMRD Parameters to Describe Molecular Reorientation

	Gd-AaLS-13^a^		Gd-OP-3_int_C^b^		Gd-OP-2_int_C^b^		Gd-OP-1_int_C^b^		Gd-OP-1_ext_C^b^	
	25 °C	37 °C	25 °C	37 °C	25 °C	37 °C	25 °C	37 °C	25 °C	37 °C
*τ*_i_ (ns)	5.2	3.5	3.7	3.3	4.0	3.2	4.3	3.3	1.8	1.1
*τ*_l_ (ns)	540	250	1800	480	320	98	68	38	78	28
*S*^2^	0.28		0.46		0.39		0.36		0.36	

a Relaxivity data in 50 mM sodium phosphate (pH 8.0), 200 mM NaCl, 5 mM EDTA.

b Relaxivity data in 25 mM Tris (pH 7.6), 200 mM NaCl, 5 mM EDTA.

The different ionic relaxivity values among the Gd-C4-protein conjugates are ascribed to the different values of the *τ*_i_ and *τ*_l_ parameters. The lower relaxivity of Gd-OP-1_ext_C compared toGd-OP-1_int_C can be ascribed to somewhat smaller *τ*_i_ values than those observed for Gd-OP-2_int_C, Gd-OP-3_int_C, and Gd-AaLS-13. For the protein cages with Gd(III) complexes on the lumenal surface, the ionic relaxivity increases with increasing number of Gd(III) complexes from Gd-OP-1_int_C to Gd-OP-2_int_C to Gd-OP-3_int_C. This is due to a *τ*_l_ that increases with the number of Gd(III) complexes in the interior of the protein cage from tens of picoseconds for Gd-OP-1_int_C to a few nanoseconds for Gd-OP-3_int_C. This effect cannot be related to magnetic coupling between Gd(III) ions, which would rather decrease relaxivity. A relatively long *τ*_l_ of a few nanoseconds is also obtained for Gd-AaLS-13.

As previously mentioned, a study of Gd(III) complexes conjugated to either the interior or exterior surface of an MS2 viral capsid showed higher relaxivities for the interior conjugation strategy.^43^ A best fit analysis of the ^1^H NMRD profiles also used the Liparis Szabo model-free approach to model the anisotropic molecular reorientation time. In this case, the Gd(III) complexes on the exterior surface showed higher local flexibility than for those on the interior surface with *τ*_l_ values of 310 and 400 ps, respectively. The different flexibility was attributed to the amino acid side chains used for exterior (lysine) or interior (tyrosine) conjugation.^49^

Transient coordination of Gd(III) to nearby charged protein residues may explain *τ*_i_ and *τ*_m_ time scales in the AaLS-13 and OP cages. Although the nature of the obtained *τ*_i_ values and the origin of the long *τ*_l_ values are not fully clear, the overall lengthening of the correlation times to values in the nanosecond time scale make these systems interesting as MR CAs. Correlation times of a few nanoseconds are in fact optimal for maximizing the relaxivity at clinical field strengths.

The time scale of *τ*_i_ corresponds to conformational flexibility of protein regions that substantially reorient the dipole–dipole interaction between Gd(III) and water protons. However, this seems unlikely due to the relatively rigid nature of the multimeric assembly. Rather, we speculate that the high flexibility of the Gd(III) complexes may allow for transient coordination of the Gd(III) ion by nearby protein residues on the nanosecond time scale. The OP cages have several negatively charged residues (Asp and Glu) near the Gd(III) binding sites that could interact with Gd(III) (Figure S26). Furthermore, increasing the number of Gd(III) complexes inside the OP cage replaces positively charged Arg residues with Cys residues that are covalently linked to Gd-C4-IA, decreasing the overall positive charge of the capsid interior and resulting in increased crowding that potentially favors the bending of the complexes toward these residues. This would explain the unexpected increase of *τ*_l_ from Gd-OP-1_int_C to Gd-OP-2_int_C to Gd-OP-3_int_C such that *τ*_l_ approaches the longer correlation time *τ*_i_ as interior space for mobility is reduced.

Similarly, for Gd-AaLS-13, the large number of negatively charged residues (Asp and Glu) that line the interior capsid surface could favor transient coordination of protein residues to Gd(III) ions (Figure S26), reducing the tag mobility (*τ*_l_) to a value similar to Gd-OP-2_int_C despite the larger interior space (Table 4). The proposed transient coordination might also facilitate exchange of coordinated water molecules on a time scale that is typical for small Gd(III) complexes (*τ*_m_ = 10^–7^ to 10^–8^ s, Tables S5 and S6), but is a remarkable result for Gd(III)-labeled proteins.

### 2.6 Solution Phantom Images

MR phantom images were used to quantify the ability of Gd-C4-protein conjugates to increase MR image contrast under mock biological conditions. Based on particle relaxivity (Table 2) and ^1^H NMRD profiles (Figure 4), Gd-AaLS-13 and Gd-OP-3_int_C were chosen for study at clinically relevant 3 T as well as high fields 7 and 9.4 T. The Gd-C4-protein conjugates were incubated in 10% fetal bovine serum (FBS) in sodium phosphate (pH 8.0) or Tris (pH 7.6) buffers. The stability of the AaLS-13 and OP cages was previously demonstrated in both human serum and FBS,^64,69^ allowing the phantom image measurements to be performed in FBS.

**Figure 5:**
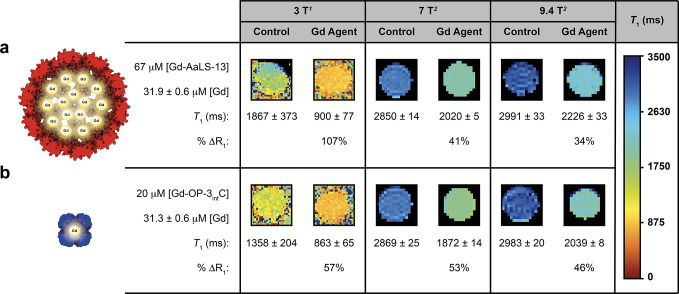
*T*_1_-weighted MR solution phantom images of (a) Gd-AaLS-13 and (b) Gd-OP-3_int_C at 3, 7, and 9.4 T. (a) Control sample of 10% FBS in 50 mM sodium phosphate (pH 8.0), 200 mM NaCl, 5 mM EDTA. The Gd-AaLS-13 sample was prepared at 67 μM with respect to monomer, and Gd(III) concentration was measured by ICP-MS. (b) Control sample of 10% FBS in 25 mM Tris-HCl (pH 7.6), 200 mM NaCl, 5 mM EDTA. The Gd-OP-3_int_C sample was prepared at 20 μM with respect to monomer, and Gd(III) concentration was measured by ICP-MS. ^1^Values at 3 T were measured using a dual gradient echo method with two different flip angles. ^2^Values at 7 and 9.4 T were obtained using a saturation recovery method.

The longitudinal relaxation rate constant (*R*_1_ = 1/*T*_1_) was measured for each sample (Figures S41−S43) and compared to a control solution to determine contrast enhancement (Eq. S3). Samples were prepared at 67 μM Gd-AaLS-13 and 20 μM Gd-OP-3_int_C with respect to the monomer, with Gd(III) concentrations measured by ICP-MS as 31.9 μM and 31.3 μM Gd(III), respectively. At 3 T, Gd-AaLS-13 increased *R*_1_ by 107%, while Gd-OP-3_int_C increased *R*_1_ by 57%. At high field strengths of 7 and 9.4 T, Gd-AaLS-13 increased *R*_1_ by 41% and 34%, respectively, whereas Gd-OP-3_int_C increased *R*_1_ by 53% and 46%, respectively (Figure 5).

In clinical MR exams, a detectable change in contrast requires an approximately 20% increase in *R*_1_.^4,5^ At all field strengths, the Gd-C4-protein conjugates show appreciable contrast enhancement (%Δ*R*_1_) relative to control conditions with 30 μM Gd(III). Considering that clinically approved Gd(III) agents require a local concentration of 125 μM of Gd(III) for detectable contrast enhancement, both Gd-AaLS-13 and Gd-OP-3_int_C show excellent contrast enhancement at four times lower Gd(III) concentration at all field strengths, but most notably at clinically relevant 3 T with 107% and 57% for Gd-AaLS-13 and Gd-OP-3_int_C, respectively. Lower concentrations of Gd-OP-3_int_C (12.5 μM monomer, 23.2 μM Gd(III)) still showed detectable contrast enhancement at 7 and 9.4 T at 32% and 27%, respectively (Figures S44, S45).

These results show that Gd-AaLS-13 and Gd-OP-3_int_C are promising candidates for *in vivo* MR imaging. However, the immunogenicity of protein-based delivery systems presents a potential limitation for biological applications, especially if multiple administrations are required. Since MR imaging would likely require only a single injection of a contrast agent, there is less concern about immunogenicity. Moreover, appending antibody binding domains to the surface of AaLS-13 was shown to mitigate the immune response.^64^ Other strategies to passivate the surface of protein scaffolds and extend the circulation times of the resulting cages have been described.^43^

## 3 Conclusions

Our results demonstrate the ability of Gd(III)-labeled AaLS-13 and OP protein cages to function as highly effective MR contrast agents. We have investigated these newly labeled cages by ^1^H NMRD to elucidate the parameters governing relaxivity. The MR performance of the protein cages can be summarized in four key results. (i) MR signal amplification was achieved through both a high payload of Gd(III) in each protein cage and increased relaxivity over the Gd(III) complex Gd-C4-IA. The Gd-C4-protein conjugates were labeled with 150 or 33 Gd(III) complexes for Gd-AaLS-13 and Gd-OP-3_int_C, respectively. The ionic relaxivity of the Gd(III)-labeled cages was increased 2.5- to 4.5-fold from Gd-C4-IA.

(ii) The increase in ionic relaxivity resulted from the τ_R_ boost common for Gd(III)-labeled NPs. Furthermore, this increase was not limited by the water exchange rate, which is in the fast exchange regime. (iii) The long *τ*_l_ values of tens to thousands of picoseconds and *τ*_m_ of 10–100 ns likely result from transient coordination of Gd(III) to charged protein residues near the covalently bound Gd(III) complex. This would also explain the increase in ionic relaxivity in the series Gd-OP-1_int_C to Gd-OP-2_int_C to Gd-OP-3_int_C. Steric crowding slows τ_l_ and favors transient Gd(III) interactions with nearby charged residues.

Finally, (iv) the parameters responsible for nuclear relaxation are optimized for high relaxivity at clinical field strengths, with coordinated water molecules in the fast exchange regime and correlation times on the nanosecond time scale. The serum phantom images at 3 T showcase this result with contrast enhancements of 57% or 107% for only 30 μM Gd(III) of Gd-AaLS-13 and Gd-OP-3_int_C, respectively.

The MR performance of Gd(III)-labeled AaLS-13 and OP cages is comparable to other previously studied Gd(III)-labeled proteins. The ionic relaxivity values at 1.4 and 7 T for Gd(III)-labeled AaLS-13 and OP compared with previously studied Gd(III)-labeled protein cages (10–18 mM^–1^ s^–1^ vs 10–60 mM^–1^ s^–1^).^45,47−55,57,58^ Only two previously studied protein cages report higher ionic relaxivity values than what is reported here (e.g., 60 mM^–1^ s^–1^).^52,54^ The differences in relaxivities reported for the Gd(III)-labeled cages likely arise from the different sizes and structures of the protein cages and the flexibility of the Gd(III) complex that is covalently bound to the protein cage.^53^

The high relaxivity of Gd(III)-protein conjugates results from the low flexibility of the covalently bound Gd(III) complex (*τ*_l_), so decreasing *τ*_l_ could result in even higher ionic relaxivity. This could be accomplished through a Gd(III) complex that employs a shorter, more rigid connecting arm,^53^ or by further increasing steric hindrance in the Gd(III)-labeled protein cages as seen in the OP variants. For example, a new variant OP-4_int_C would presumably show even higher relaxivity than Gd-OP-1_int_C, Gd-OP-2_int_C, and Gd-OP-3_int_C. Alternatively, the long correlation time (*τ*_i_) could be slowed to approach expected values for *τ*_R_, even though the nature of *τ*_i_ is currently not fully understood.

Gd(III)-labeled AaLS-13 and OP protein cages represent excellent platforms for a variety of MR imaging applications. The pharmacokinetics of Gd(III)-labeled cages can be studied by *in vivo* MR fate mapping or *ex vivo* biodistribution. Alternatively, these versatile cages can be modified to incorporate surface modifications that alter biodistribution and/or bind specific cell surface receptors for targeted molecular imaging and theranostic platforms.

## 4 Experimental Methods

Details of experimental methods are included in the Supporting Information. No unexpected or unusually high safety hazards were encountered.

## Supporting Information Available

Syntheses, NMR spectra, mass spectrometry traces, HPLC analyses, protein sequences, protein structures, SEC profiles, and general experimental methods (PDF)

## Abbreviations

CAs: contrast agents
CH_2_Cl_2_: dichloromethane
DIC: diisopropylcarbodiimide
DIEA: diisopropylethylamine
DMF: dimethylformamide
DOTA: 1,4,7,11-tetraazacyclododecane
EDTA: ethylenediaminetetraacetic acid
equiv: equivalent
FBS: fetal bovine serum
GdCl_3_·6H_2_O: gadolinium(III) chloride hexahydrate
^1^H: proton
H_2_: dihydrogen gas
H_2_O: water
K_2_CO_3_: potassium carbonate
MeCN: acetonitrile
MR: magnetic resonance
MQ: miili-Q direct water purification system
NaCl: sodium chloride
NHS: *N*-hydroxysuccinimide
NMRD: nuclear magnetic relaxation dispersion
N_2_: dinitrogen gas
NP: nanoparticle
Pd/C: palladium on carbon catalyst
RT: room temperature
SBM: Solomon-Bloembergen-Morgan
SNR: signal-to-noise ratio
tBu: tertiary butyl
Tris: tris(hydroxymethyl)aminomethane
^*t*^BuDO3A: tri-^*t*^Bu 2,2′,2″-(1,4,7,10-tetraazacyclododecane-1,4,7-triyl)triacetate
TFA: trifluoroacetic acid
wt: wild type
ZFS: zero-field splitting
