# Supplementary material for: Engineered Nonviral Protein Cages Modified for MR Imaging
Source: ACS Appl Bio Mater. 2023 Jan 10;6(2):591–602. doi: 10.1021/acsabm.2c00892 (PMC9945100; doi:10.1021/acsabm.2c00892)
Supplement: Supplementary file 1 — mt2c00892_si_001.pdf [file mt2c00892_si_001.pdf]

Supporting Information

Title: Engineered Nonviral Protein Cages Modified for MR Imaging

Authors: Megan A. Kaster,[†] Mikail D. Levasseur,[‡] Thomas G. W. Edwardson,[‡] Michael A. Caldwell,[†] Daniela Hofmann,[‡] Giulia Licciardi,[§,ǁ,⊥] Giacomo Parigi,[§,ǁ,⊥] Claudio Luchinat,[§,ǁ,⊥] Donald Hilvert,[*,‡] and Thomas J. Meade[*,†]

[†]Departments of Chemistry, Molecular Biosceinces, Neurobiology and Radiology, Northwestern University, 2145 N. Sheridan Road, Evanston, IL 60208, United States

[‡]Laboratory of Organic Chemistry, ETH Zurich, Vladimir-Prelog-Weg 1-5/10, 8093 Zürich, Switzerland

[§]Magnetic Resonance Center (CERM), University of Florence, via Luigi Sacconi 6, Sesto Fiorentino, 50019 Italy

[ǁ]Department of Chemistry "Ugo Schiff", University of Florence, via della Lastruccia 3, Sesto Fiorentino, 50019 Italy

[⊥]Consorzio Interuniversitario Risonanze Magnetiche Metallo Proteine (CIRMMP), via Luigi Sacconi 6, Sesto Fiorentino, 50019 Italy

***Email:** tmeade@northwestern.edu; hilvert@org.chem.ethz.ch

Table of Contents

1. General Methods S4
2. Synthetic Route of **Gd-C4-IA** S6

*Synthesis of **benzyl acrylate*** S6

*Synthesis of tri-tert-butyl 2,2',2''-(1,4,7,10-tetraazacyclododecane-1,4,7-triyl)triacetate (^{t}**Bu DO3A**)* S6

*Synthesis of tri-tert-butyl 2,2',2''-(10-(3-(benzyloxy)-3-oxopropyl)-1,4,7,10-tetraazacyclododecane-1,4,7-triyl)triacetate (**1**)* S7

*Synthesis of 3-(4,7,10-tris(2-(tert-butoxy)-2-oxoethyl)-1,4,7,10-tetraazacyclododecan-1-yl)propanoic acid (**2**)* S8

*Synthesis of tri-tert-butyl 2,2',2''-(10-(3-((4-((tert-butoxycarbonyl)amino)butyl)amino)-3-oxopropyl)-1,4,7,10-tetraazacyclododecane-1,4,7-triyl)triacetate (**3**)* S9

*Synthesis of 2,2',2''-(10-(3-((4-aminobutyl)amino)-3-oxopropyl)-1,4,7,10-tetraazacyclododecane-1,4,7-triyl)triacetic acid (**4**)* S10

*Synthesis of 2,2',2''-(10-(3-((4-aminobutyl)amino)-3-oxopropyl)-1,4,7,10-tetraazacyclododecane-1,4,7-triyl)triacetate gadolinium(III) (**Gd-C4-NH$_2$**)* S10

*Synthesis of 2,2',2''-(10-(3-((4-(2-iodoacetamido)butyl)amino)-3-oxopropyl)-1,4,7,10-tetraazacyclododecane-1,4,7-triyl)triacetate gadolinium(III) (**Gd-C4-IA**)* S11

3. NMR Spectra of **Gd-C4-IA** Synthons S12
4. MS Spectra of **Gd-C4-IA** Synthons S27
5. HR-MS Spectrum of **Gd-C4-IA** S33
6. HPLC Traces of Gd(III) Complexes S35
7. Preparation and Characterization of Protein Cages S36

Protein sequences S36

Cloning of OP variants S37

Protein expression of AaLS-13 and OP cysteine mutants S37

Cell lysis and Ni-NTA purification of AaLS-13 .... S37

Cell lysis and Ni-NTA purification of OP variants S38

Sulfhydryl groups on protein cages S39

Selected residues in close proximity to reactive sites S40

8. Protein Conjugation with **Gd-C4-IA** S41

*Protein labeling with **Gd-C4-IA*** S41

MS Spectra of Gd-Protein Conjugates S42

UV-vis spectroscopy of Gd(III) complexes S45

Quantification of protein cage labeling with Gd(III) S46

9. Relaxivity Measurements S47
Relaxivity measurements at 1.4 T S47
Relaxivity measurements at 7 T S47
Relaxivity measurements data at 1.4 T and 7 T S49
Determination of relaxivity values S54
10. ^{1}H NMRD Measurements S55
^{1}H NMRD measurements of AaLS-13 and OP S55
Parameters from best fit profiles of ***Gd-C4-IA*** S56
Parameters from best fit profiles of Gd-labeled proteins S57
11. Solution Phantom Images S59
Solution phantom image sample preparation S59
Phantom image measurements at 3 T S59
Phantom image measurements at 7 T S59
Phantom image measurements at 9.4 T S60
Equation for ΔR_1 .... S60
Phantom image data S61
Phantom image analysis S63
12. References S65

1. General Methods

Unless otherwise indicated, all reactions were performed under a nitrogen atmosphere using oven-dried glassware. Anhydrous solvents were used in all reactions and obtained from a J.C. Meyer solvent system (Laguna Beach, CA). Thin-layer chromatography (TLC) was performed on EMD 60 F254 silica gel plates. Standard grade 60 Å 230–400 mesh silica gel was used for normal-phase column chromatography. Unless otherwise stated, all silica gel columns were flashed with air. ^{1}H and ^{13}C NMR spectra were obtained on a Bruker 500 MHz Avance III NMR spectrometer with DCH cryoprobe. ESI-MS was performed on a Bruker AmaZon-SL spectrometer.

Cyclen was purchased from Strem Chemical. Ethylenediaminetetraacetic acid (EDTA) was purchased from AppliChem GmbH. All other buffer components, salts, and reagents were purchased from Sigma Aldrich, Merck KGaA, Fisher Scientific, Acros Organics, or TCI and used without purification.

Analytical HPLC-MS was performed on an Agilent 1260 Infinity II HPLC system with an in-line Agilent 6120 Quad mass spectrometer. Semi-preparative HPLC was performed on an Agilent PrepStar 218 equipped with an Agilent 1260 Infinity diode array detector. HPLC purifications utilized deionized water (18.2 MΩ·cm) obtained from a Millipore Q-Guard System and HPLC grade MeCN, formic acid, and ammonium hydroxide (all obtained from Fisher Scientific). Analytical HPLC-MS used an Atlantis C18 column (4.6 × 250 mm, 5 µm). Semipreparative HPLC used an Atlantis T3 C18 column (19 x 250 mm, 10 µm). **Gd-C4-NH$_2$** and **Gd-C4-IA** were purified using the following method: MeCN held at 0% for 5 min followed by a 20 min ramp to 100%.

All restriction enzymes, T4 polynucleotide kinase (PNK), Phusion® High-Fidelity DNA polymerase, and T4 DNA ligase were obtained from New England BioLabs. Oligonucleotides were synthesized by Microsynth AG. Isopropyl β-D-1-thiogalactopyranoside (IPTG) was purchased from Fluorochem. Kanamycin sulfate was obtained from AppliChem GmbH. Ni-NTA agarose resin was obtained from Qiagen GmbH. Amicon® Ultra centrifugal filters were purchased from Merck and PD Mini-/MidiTrap desalting columns from GE Healthcare. Millipore purification system was used to obtain Milli-Q water. All buffers were prepared using Milli-Q (MQ) water, pH adjusted for the temperature at which the buffer was used, and sterile-filtered (0.2 µm membrane filter).

DNA and protein quantification were carried out using a NanoDrop 2000c spectrophotometer from ThermoFisher Scientific Inc. Size-exclusion chromatography (SEC) was carried out on an NGC™ Medium-Pressure Chromatography System from Bio-Rad Laboratories, Inc., unless otherwise

mentioned. Transmission electron microscopy (TEM) images were obtained on a Morgagni 268 from FEI.

UV-vis spectroscopy was performed on an Agilent Technologies Cary 60 spectrophotometer.

2. Synthetic Route of **Gd-C4-IA**

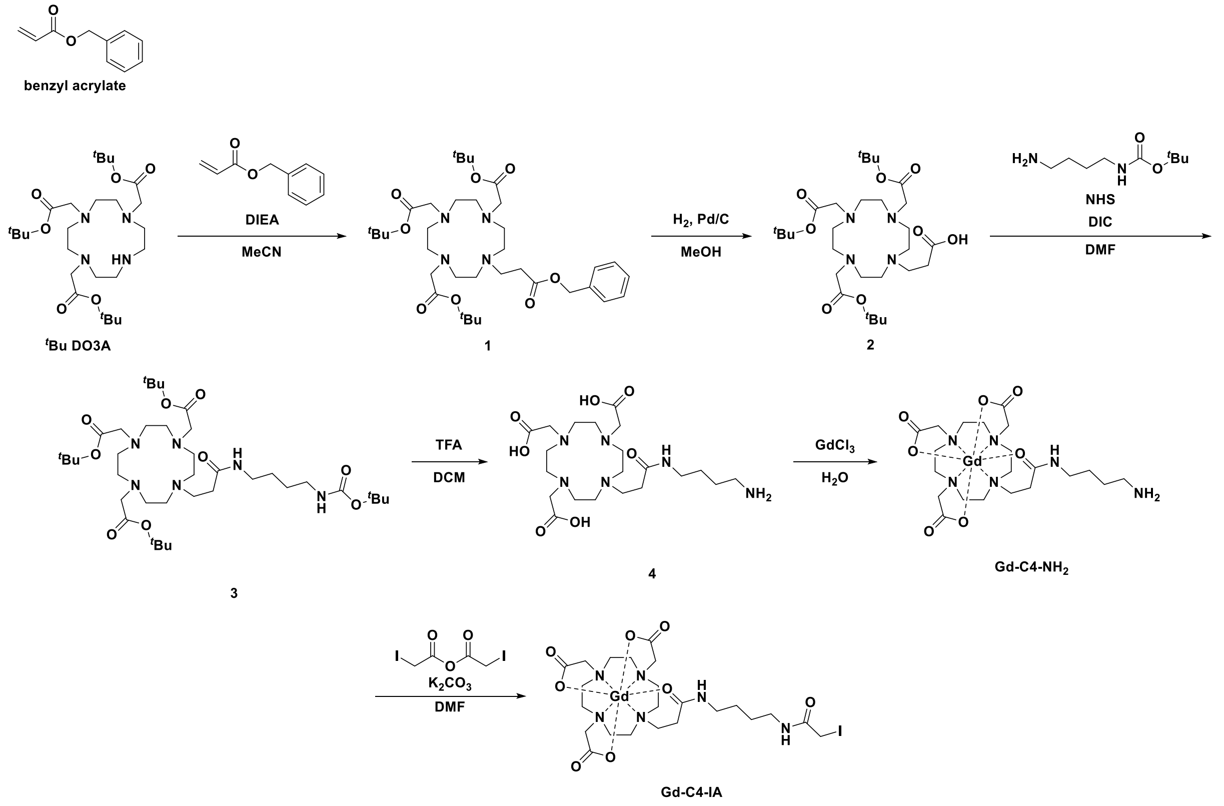

Scheme S1. Synthetic preparation of **Gd-C4-IA** from starting components **benzyl acrylate** and ***ᵗ*BuDO3A**.

Synthesis of benzyl acrylate

Benzyl acrylate was synthesized following literature procedure.[1]

Synthesis of tri-tert-butyl 2,2',2''-(1,4,7,10-tetraazacyclododecane-1,4,7-triyl)triacetate (ᵗBu DO3A)

ᵗBu DO3A was synthesized following literature procedure.[2, 3]

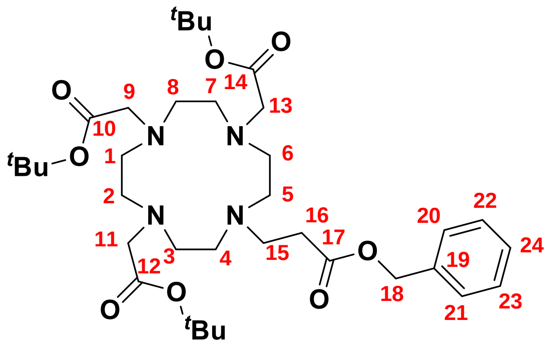

Structure of **1**

Synthesis of tri-tert-butyl 2,2',2''-(10-(3-(benzyloxy)-3-oxopropyl)-1,4,7,10-tetraazacyclododecane-1,4,7-triyl)triacetate (1)

To a stirred solution of ***t*Bu DO3A** (500 mg, 0.971 mmol, 1 equiv.) and **benzyl acrylate** (217.9 mg, 1.94 mmol, 2 equiv.) in anhydrous MeCN (30 mL) was slowly added DIPEA (1 mL, 5.74 mmol, 5.9 equiv.) at room temperature under N_2 (g). The mixture was stirred for 72 hours at room temperature until starting material was consumed (monitored by TLC). The reaction mixture was dried by reduced pressure and the clear residue brought up in 50 mL CH_2Cl_2. The organic layer was washed with saturated $NaHCO_3$ aq. (3 x 20 mL) and saturated NaCl aq. (1 x 20 mL). The organic layer was dried over Na_2SO_4 and concentrated under reduced pressure. The off-white solid was purified by flash chromatography in 1:20 MeOH:CH_2Cl_2 (Rf = 0.25, stained by Pt) to yield **1** as a pale yellow oil (353.7 mg, 47.3% yield). ^{1}H NMR (500 MHz, $CDCl_3$): δ = 7.35 (m, 3 Hs, 22-24), 7.30 (m, 2 Hs, 20-21), 5.09 (s, 2Hs, 18), 3.48 (d, *J* = 4.9 Hz, 4 H, 9, 11, 13), 2.77 – 3.37 (br overlapping signals), 2.55 (t, *J* = 7.2 Hz, 2 H, 16), 2.32 (br s), 1.73 (br s, 5 Hs), 1.43 – 1.46 (br s, 27 Hs, $C(C\underline{H_3})_3$); ^{13}C NMR (500 MHz, $CDCl_3$): δ = 172.9 (10), 172.6 (12), 172.2 (13), 135.3 (19), 128.4 (22-23), 128.2 (24), 127.8 (20-21), 82.3 ($\underline{C}(CH_3)_3$), 82.1 ($\underline{C}(CH_3)_3$), 66.2 (18), 56.3, 55.5, 49.8 (9, 11, 13), 30.8 (16), 27.7 (trans pair of $C(\underline{C}H_3)_3$), 27.6 (single $C(\underline{C}H_3)_3$); ESI/MS$^+$ m/z = 677.638 $[M+H]^+$, 699.621 $[M+Na]^+$.

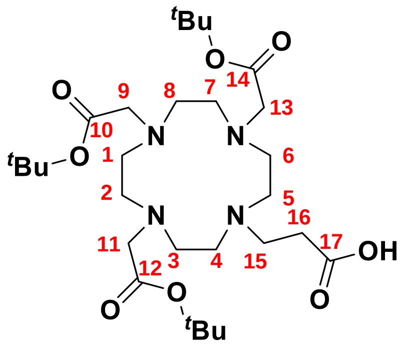

Structure of **2**

Synthesis of 3-(4,7,10-tris(2-(tert-butoxy)-2-oxoethyl)-1,4,7,10-tetraazacyclododecan-1-yl)propanoic acid (2)

A stirring solution of **1** (452.3 mg, 0.668 mmol, 1 equiv.) and Pd/C (approximately 1 g) in 10 mL MeOH was purged with H_2 (g) (3 x 100 mL) and then stirred under H_2 (g) for 24 hours The solution was filtered through celite and washed with MeOH (3 x 20 mL). Analysis by MS and TLC suggested the reaction was not complete, so the organic layer was concentrated under reduced pressure. Again, the stirring solution of **1** and Pd/C in 20mL MeOH was purged with H_2 (g) (3 x 100 mL) and then stirred under H_2 (g) for 18 hours The solution was filtered through celite and washed with MeOH (3 x 20 mL). The organic layer was concentrated under reduced pressure, and the resulting yellow oil purified by flash chromatography in 1:19 MeOH:CH_2Cl_2 to 1:9 MeOH:CH_2Cl_2 to 1:9:40 NH_4OH:MeOH:CH_2Cl_2 ($Rf_{1:9\ MeOH:CH2Cl2}$ = 0.31, stained by Pt) to yield **2** as a pale yellow oil (107.0 mg, 27.3% yield). ^{1}H NMR (500 MHz, CD_3OD): δ = 3.39 (br s, 0.5 Hs, 9, 11, 13), 2.56 – 3.39 (br overlapping signals, 16 Hs), 2.53 (t, *J* = 7.4 Hz, 2 Hs, 16), 1.45-1.46 (overlapping s, 27 Hs , C(C<u>H</u>$_3$)$_3$); ^{13}C NMR (500 MHz, CD_3OD): δ = 83.0 (<u>C</u>(CH_3)$_3$), 57.3 (11, 13), 56.6 (9), 51.5, 49.9, 32.4 (16), 28.4 (trans pair of C(<u>C</u>H$_3$)$_3$), 28.4 (single C(<u>C</u>H$_3$)$_3$); ESI/MS$^+$ m/z = 587.604 $[M+H]^+$, 609.574 $[M+Na]^+$, 1218.422 $[2M+H+Na]^+$.

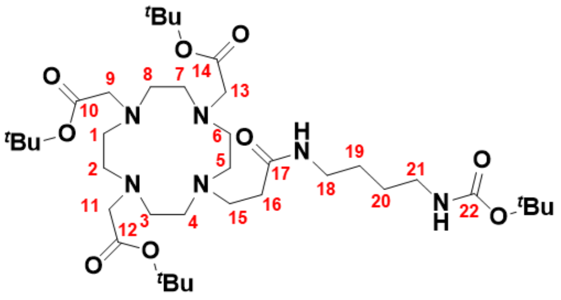

Structure of **3**

Synthesis of tri-tert-butyl 2,2',2''-(10-(3-((4-((tert-butoxycarbonyl)amino)butyl)amino)-3-oxopropyl)-1,4,7,10-tetraazacyclododecane-1,4,7-triyl)triacetate (3)

To a stirring solution of **2** (107.0 mg, 0.182 mmol, 1 equiv.) and NHS (62.96 mg, 0.547 mmol, 3 equiv.) in 10 mL anhydrous DMF was added DIEA (160 µL, 0.912 mmol, 5 equiv.) and DIC (150 µL, 0.912 mmol, 5 equiv.). To this stirring solution was dropwise added t-butyl (4-aminobutyl)carbamate (136.2 mg, 0.279 mmol, 1.5 equiv.) in 10 mL DMF over the course of 40 min and the solution stirred under N_2 (g) for 24 hours The solvent was concentrated under reduced pressure, and the resulting yellow oil dissolved in CH_2Cl_2 and washed with saturated $NaHCO_3$ (3 x 20 mL) and saturated NaCl aq. (3 x 20 mL). The organic layer was dried over Na_2SO_4 and concentrated under reduced pressure. The pale yellow oil was purified by flash chromatography in 1:20 MeOH:CH_2Cl_2 to 1:10 MeOH:CH_2Cl_2 ($Rf_{1:9\ MeOH:CH2Cl2}$ = 0.5, stained by Pt) to yield **3** as a pale yellow oil (150.1 mg, quantitative yield). ^{1}H NMR (500 MHz, CD_3OD): δ = 3.34 (t, *J* = 6.5 Hz, 2 Hs, 15), 3.16 (m, 2Hs, 21), 3.08 (m, 2 Hs, 18), 3.02 (m, 4 Hs, 3-6), 2.33 (t, *J* = 6.6 Hz, 2 Hs, 16), 1.44 – 1.49 (m, 12 Hs, C(C$\underline{H_3}$)$_3$), 1.41 (s, 17Hs, C(C$\underline{H_3}$)$_3$); ^{13}C NMR (500 MHz, CD_3OD): δ = 174.1 (10, 12, 14), 161.0 (17), 158.5 (22), 79.9 ($\underline{C}$(CH$_3$)$_3$), 79.8 ($\underline{C}$(CH$_3$)$_3$), 41.0 (overlapping s, 3, 4, 5, 6), 40.7 (18), 40.0 (21), 37.7 (16), 37.6 (15), 28.8 (NCH$_3$COOC(C$\underline{H_3}$)$_3$), 28.6 (20), 28.3 (NHCOOC(C$\underline{H_3}$)$_3$), 27.7 (19); ESI/MS$^+$ m/z = 757.803 [M+H]$^+$.

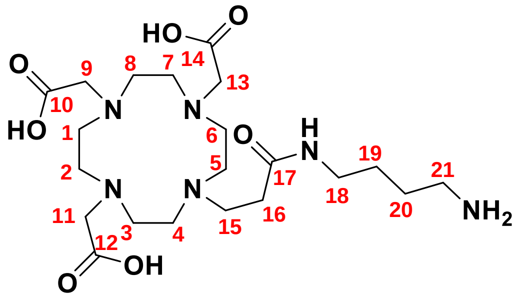

Structure of **4**

Synthesis of 2,2',2''-(10-(3-((4-aminobutyl)amino)-3-oxopropyl)-1,4,7,10-tetraazacyclododecane-1,4,7-triyl)triacetic acid (4)

A solution of **3** (10.8083 mg, 14.3 mmol) in 4:1 TFA:CH_2Cl_2 (100 mL) was stirred under N_2 (g) for 18 hours The solvent was concentrated under reduced pressure, and the resulting brown oil (crude **4**) was carried on crude to the next reaction. ESI/MS$^+$ m/z = 489.394 [M+H]$^+$.

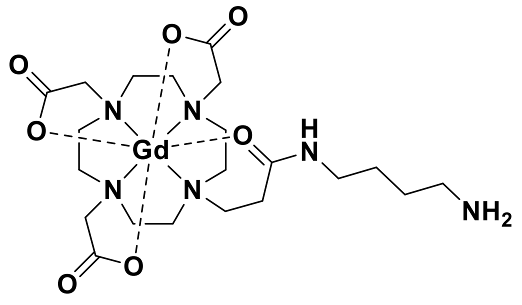

Structure of **Gd-C4-NH$_2$**

Synthesis of 2,2',2''-(10-(3-((4-aminobutyl)amino)-3-oxopropyl)-1,4,7,10-tetraazacyclododecane-1,4,7-triyl)triacetate gadolinium(III) (Gd-C4-NH$_2$)

A crude solution of **4** (7 g, 14.3 mmol, 1 equiv.) in H_2O (100 mL) was adjusted to pH 6.5 with 0.5 M HCl. To this stirring solution, a solution of $GdCl_3$ hexahydrate (6.9763 g, 18.8 mmol, 1.3 equiv.) in H_2O (15 mL) was added. The pH was maintained at 6.5. The reaction was stirred at room temperature under N_2 (g) overnight. The solution was adjusted to neutral pH with 0.5 M NaOH and filtered for HPLC. **Gd-C4-NH$_2$** was collected from the HPLC at 12.1 min. as a yellow oil (3.3685 g, 34 % yield over 2 steps). ESI/MS$^+$ m/z = 644.314 [M+ H]$^+$; HRMS (ESI) m/z = 644.2057 (calcd. 644.2038) [M+H]$^+$

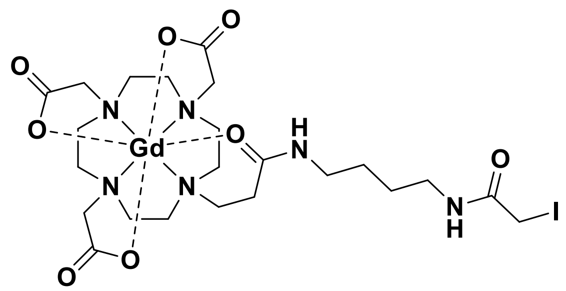

Structure of **Gd-C4-IA**

Synthesis of 2,2',2''-(10-(3-((4-(2-iodoacetamido)butyl)amino)-3-oxopropyl)-1,4,7,10-tetraazacyclododecane-1,4,7-triyl)triacetate gadolinium(III) (Gd-C4-IA)

The materials **Gd-C4-NH$_2$** (187.4 mg, 0.235 mmol, 1 equiv.), iodoacetic anhydride (321.3 mg, 0.706 mmol, 3 equiv.), and K_2CO_3 (99.01 mg, 0.706 mmol, 3 equiv.) were combined and placed under vacuum for 30 min. The materials were then dissolved in 10 mL DMF at 0 °C and stirred under N_2 (g) for 24 hours, allowing the solution to warm to room temperature. The solvent was concentrated by reduced pressure, and the pale yellow residue dissolved in H_2O and adjusted to pH 7. The solution was filtered for HPLC, and **Gd-C4-IA** was collected from the HPLC at 13.5 min. as a white fluffy powder (46.3 mg, 19.6% yield). ESI/MS$^+$ m/z = 811.278 $[M]^+$, 833.257 $[M+Na]^+$, 849.218 $[M+K]^+$; HRMS (ESI) m/z = 812.1113 (calcd. 812.1110) $[M+H]^+$

3. NMR Spectra of **Gd-C4-IA** Synthons

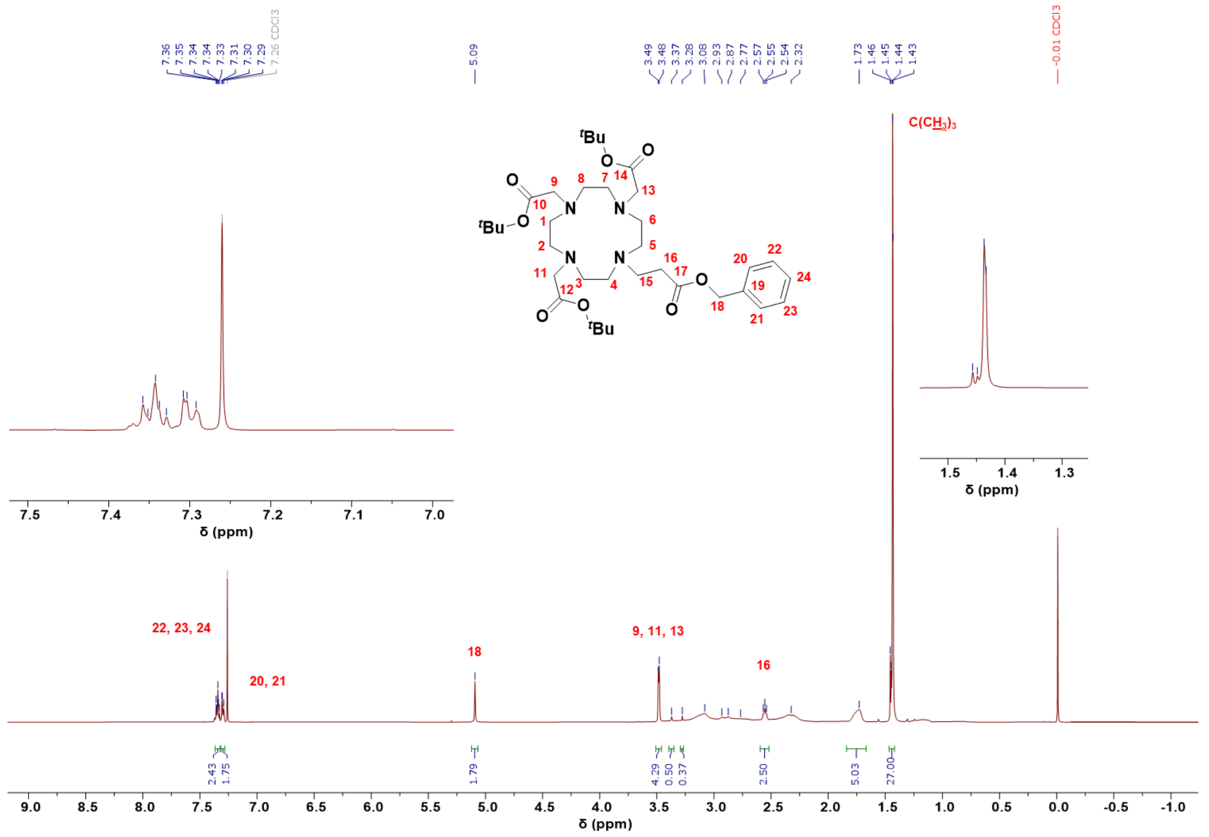

Figure S1. ^{1}H NMR Spectrum of **1** in $CDCl_3$ at 500 MHz.

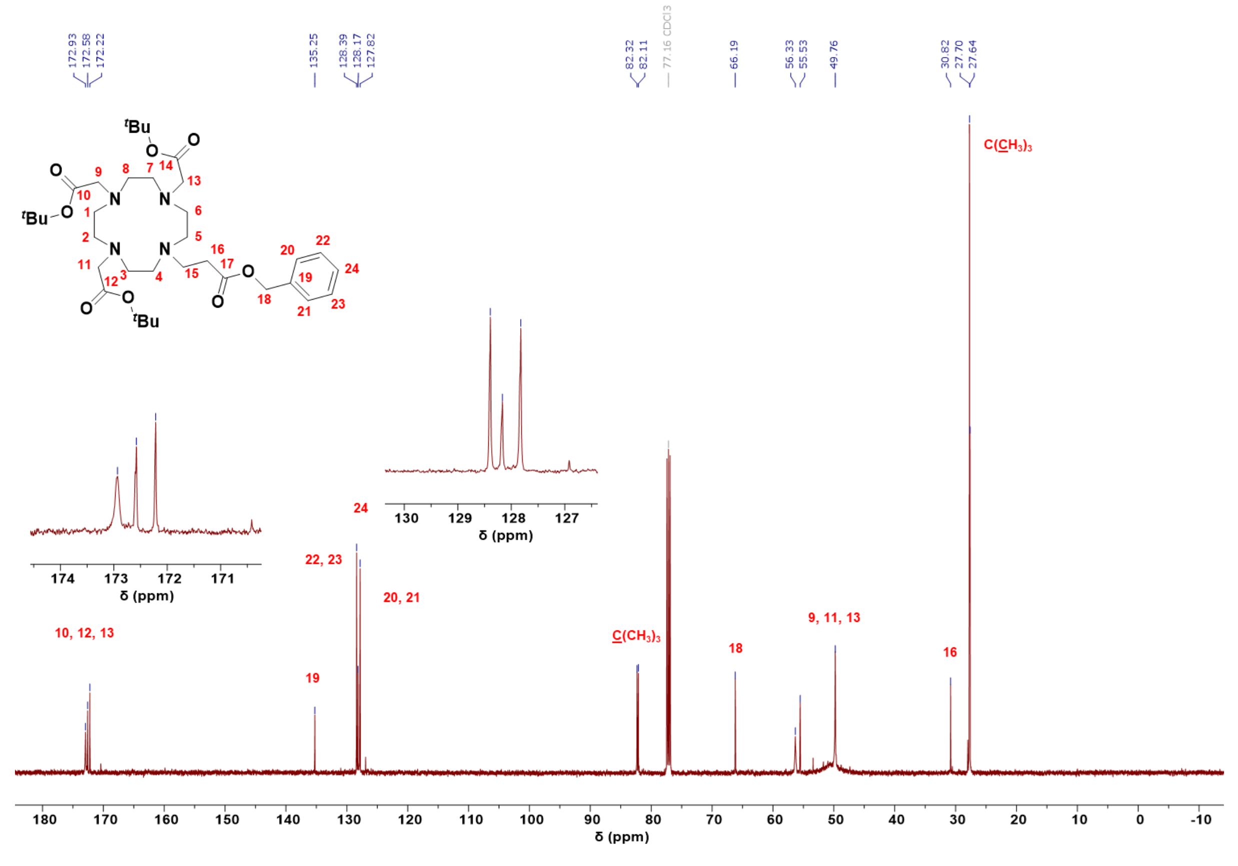

Figure S2. ^{13}C NMR Spectrum of **1** in $CDCl_3$ at 500 MHz.

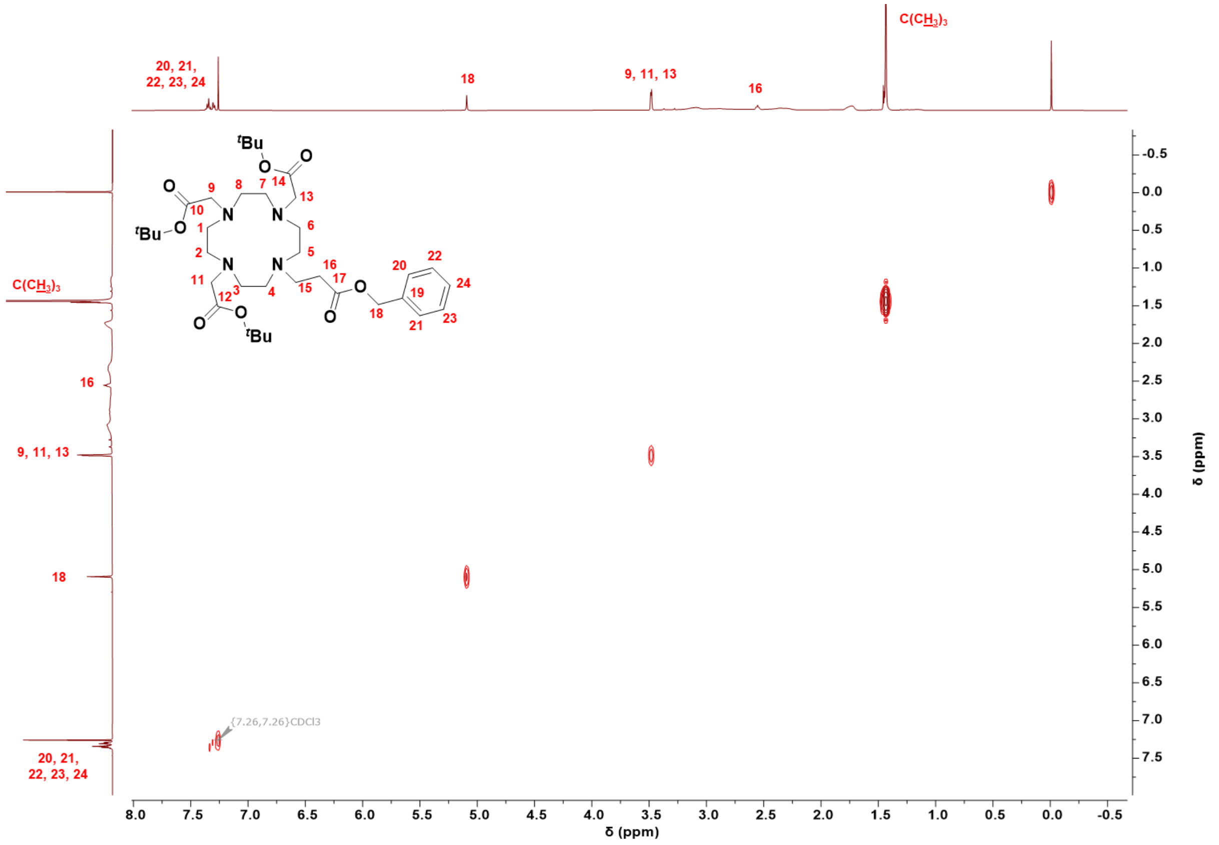

Figure S3. ^1H-^1H COSY Spectrum of **1** in $CDCl_3$ at 500 MHz.

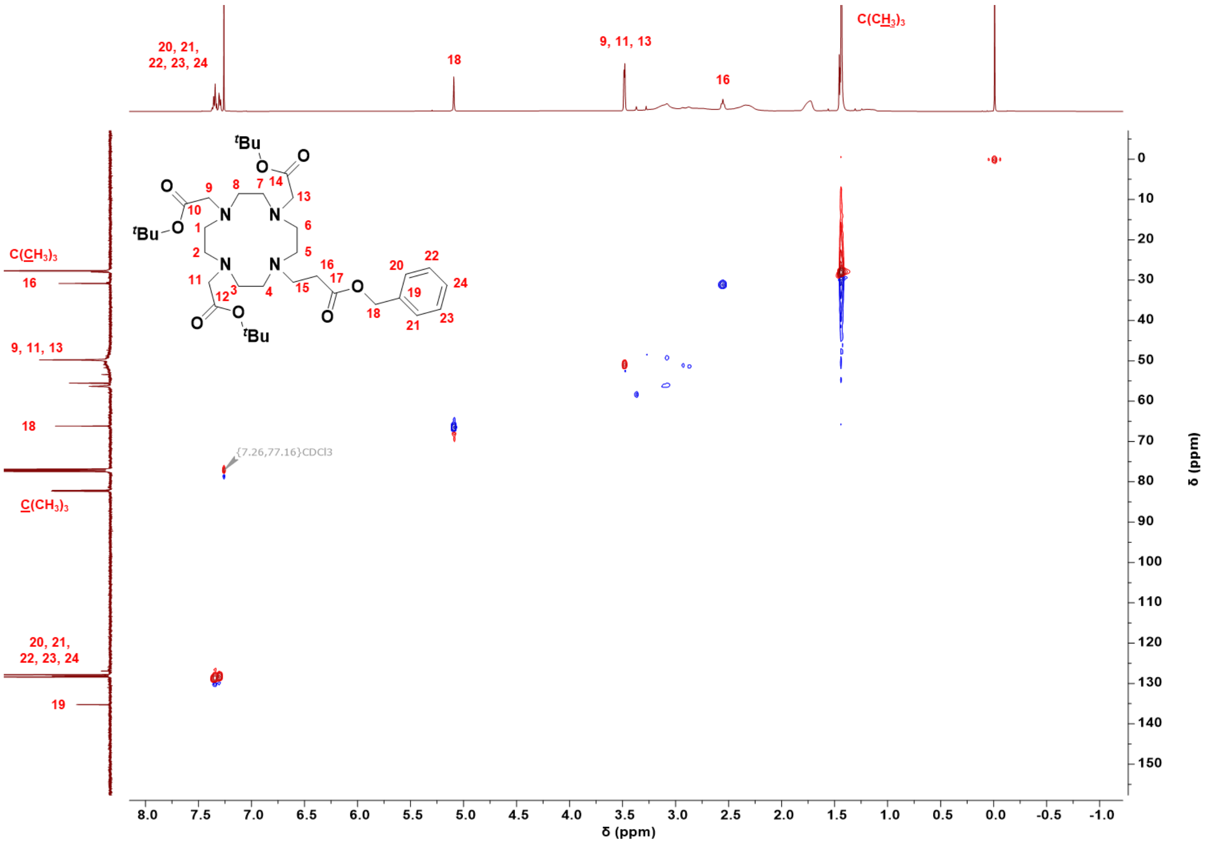

Figure S4. ^{1}H-^{13}C HSQC Spectrum of **1** in $CDCl_3$ at 500 MHz.

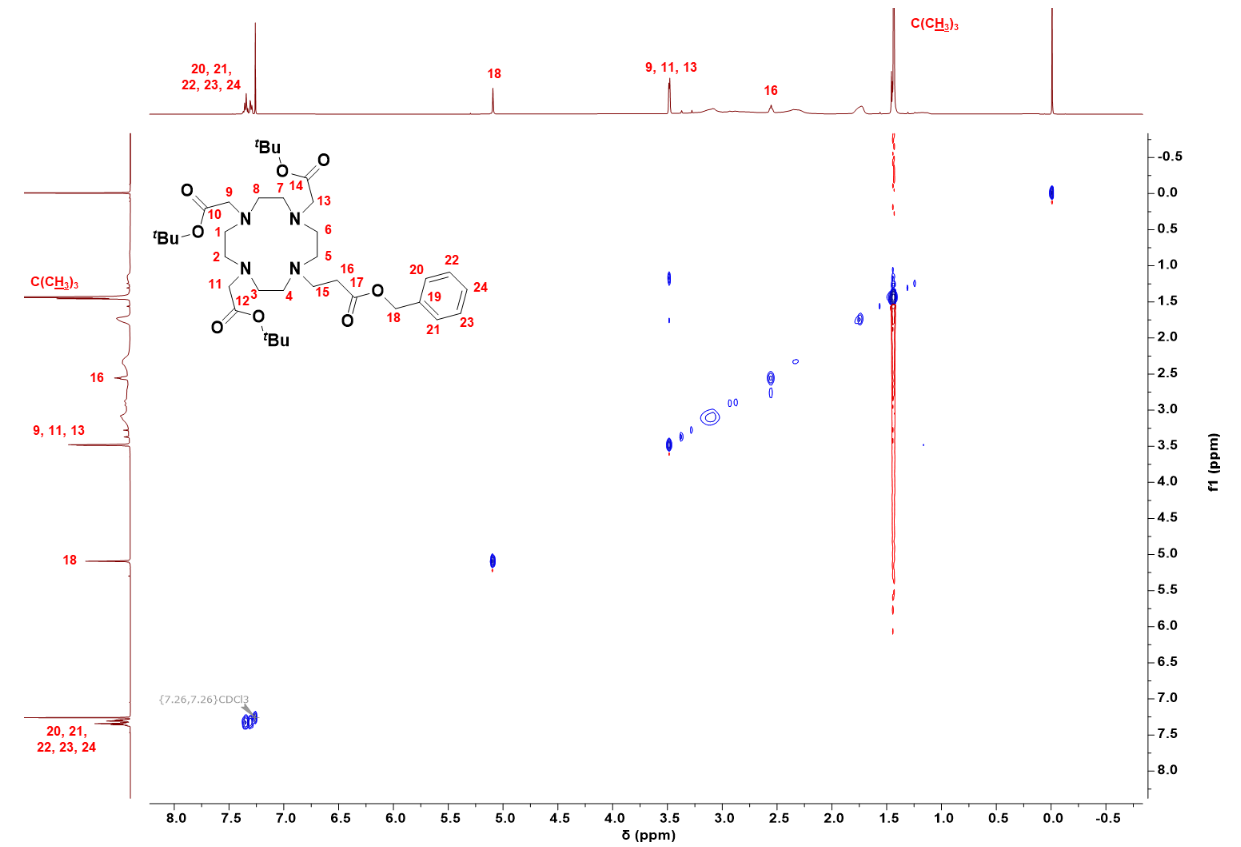

Figure S5. ^{1}H-^{1}H TOCSY Spectrum of **1** in $CDCl_3$ at 500 MHz.

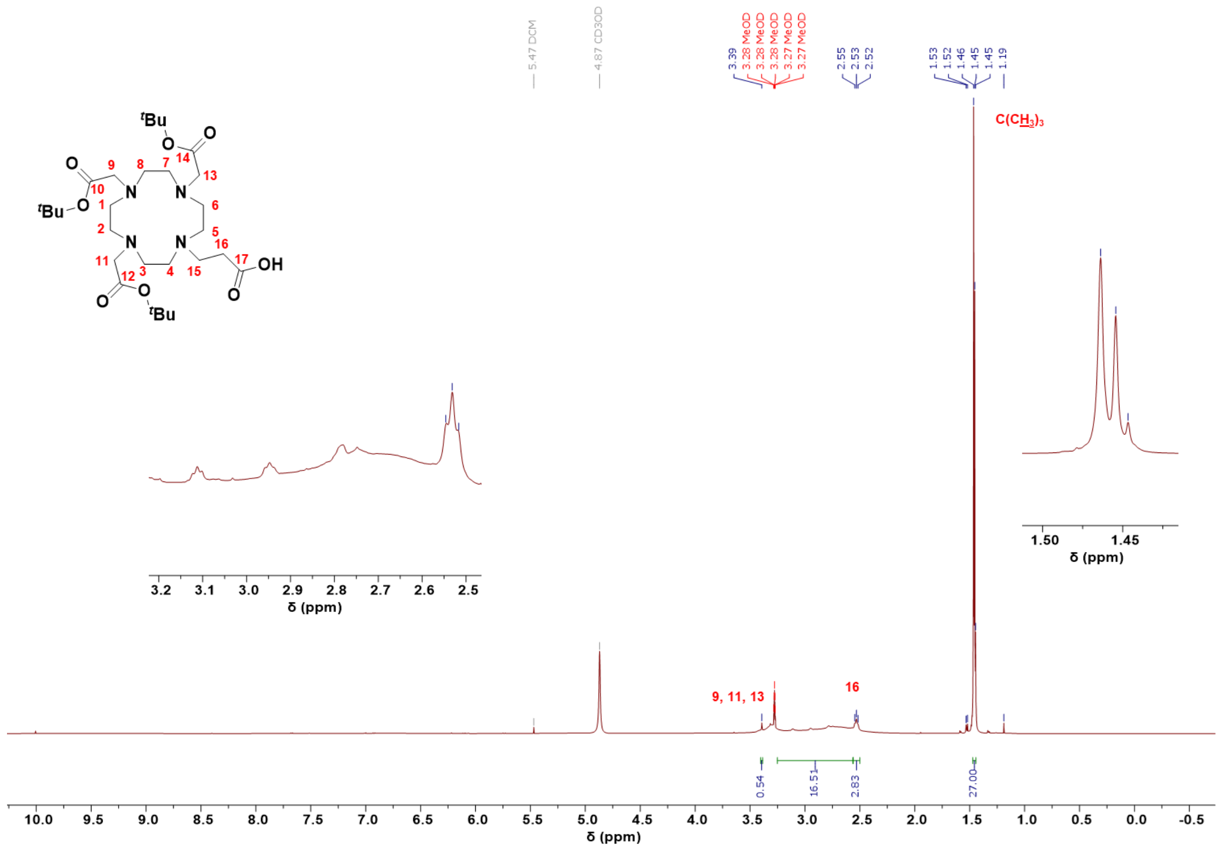

Figure S6. ^{1}H Spectrum of **2** in CD_3OD at 500 MHz.

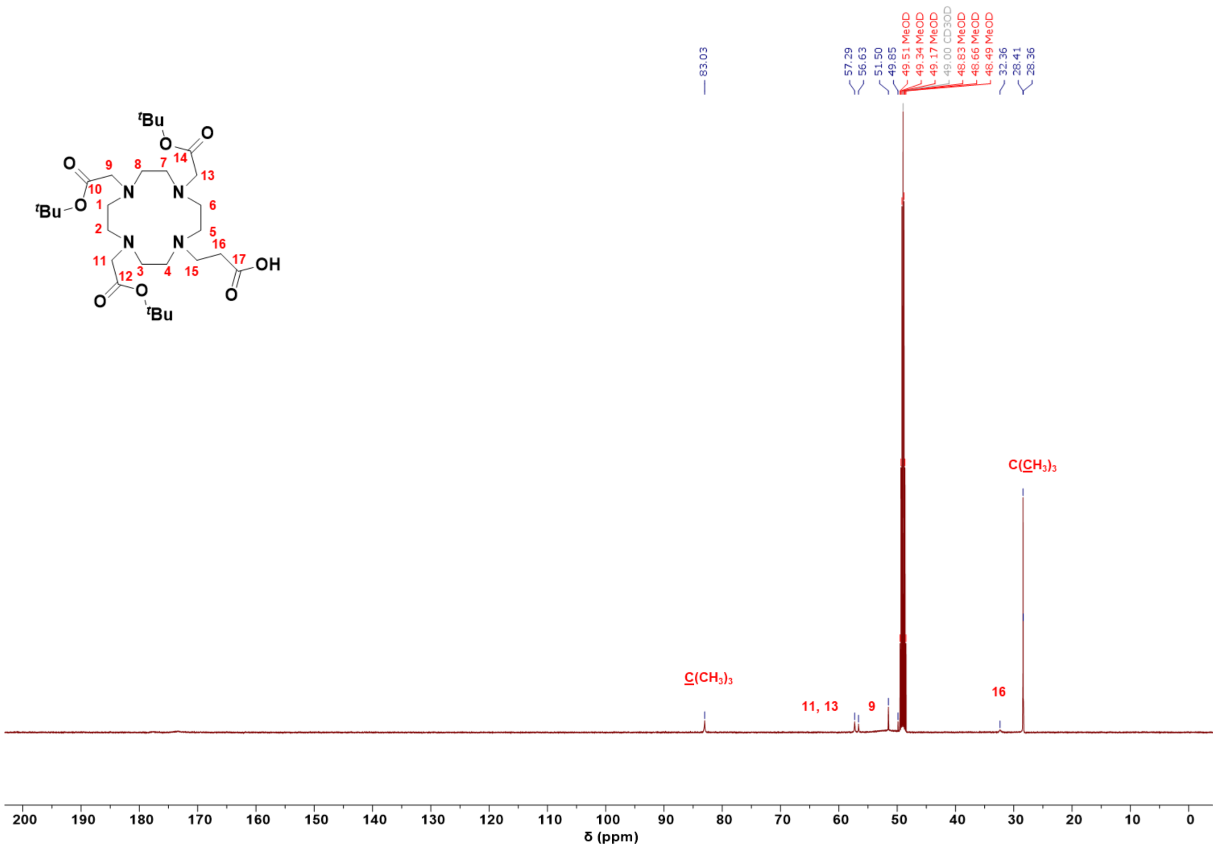

Figure S7. ^{13}C Spectrum of **2** in CD_3OD at 500 MHz.

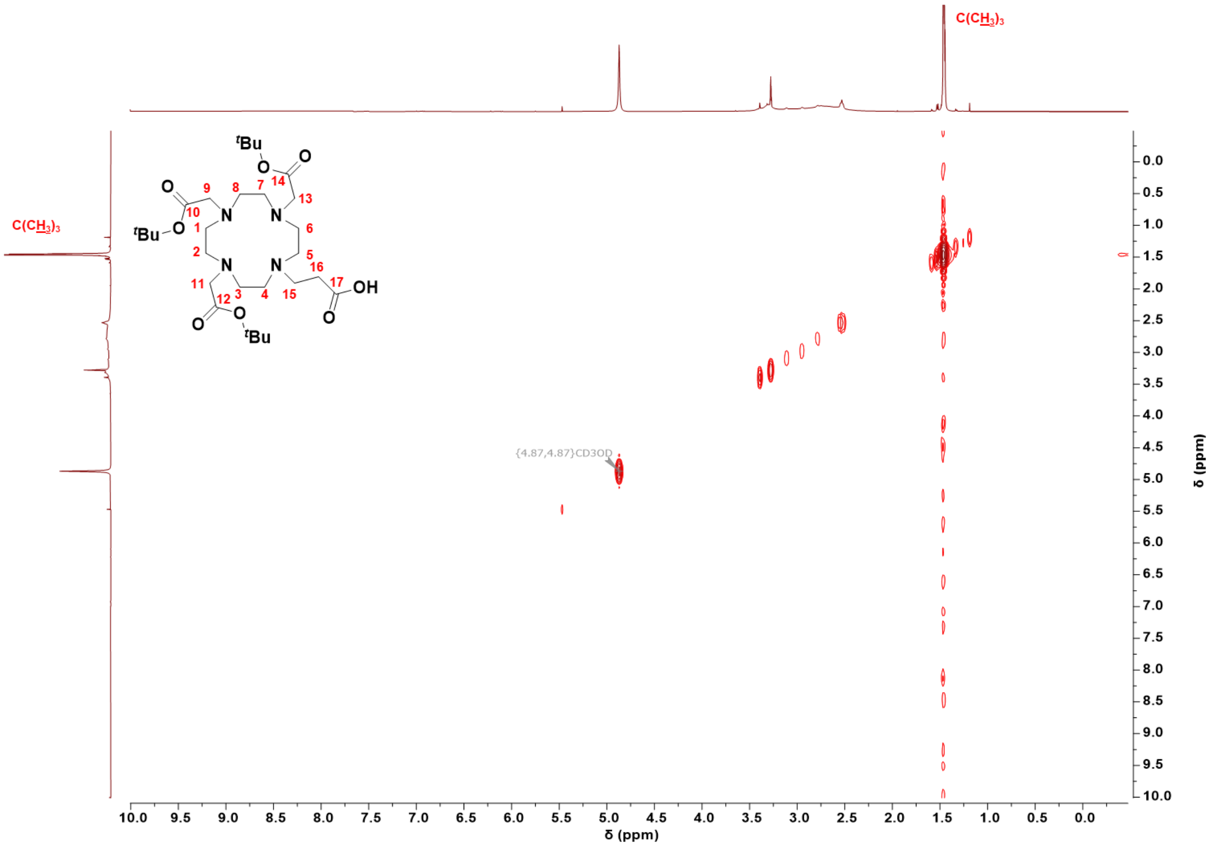

Figure S8. ^{1}H-^{1}H COSY Spectrum of **2** in CD_3OD at 500 MHz.

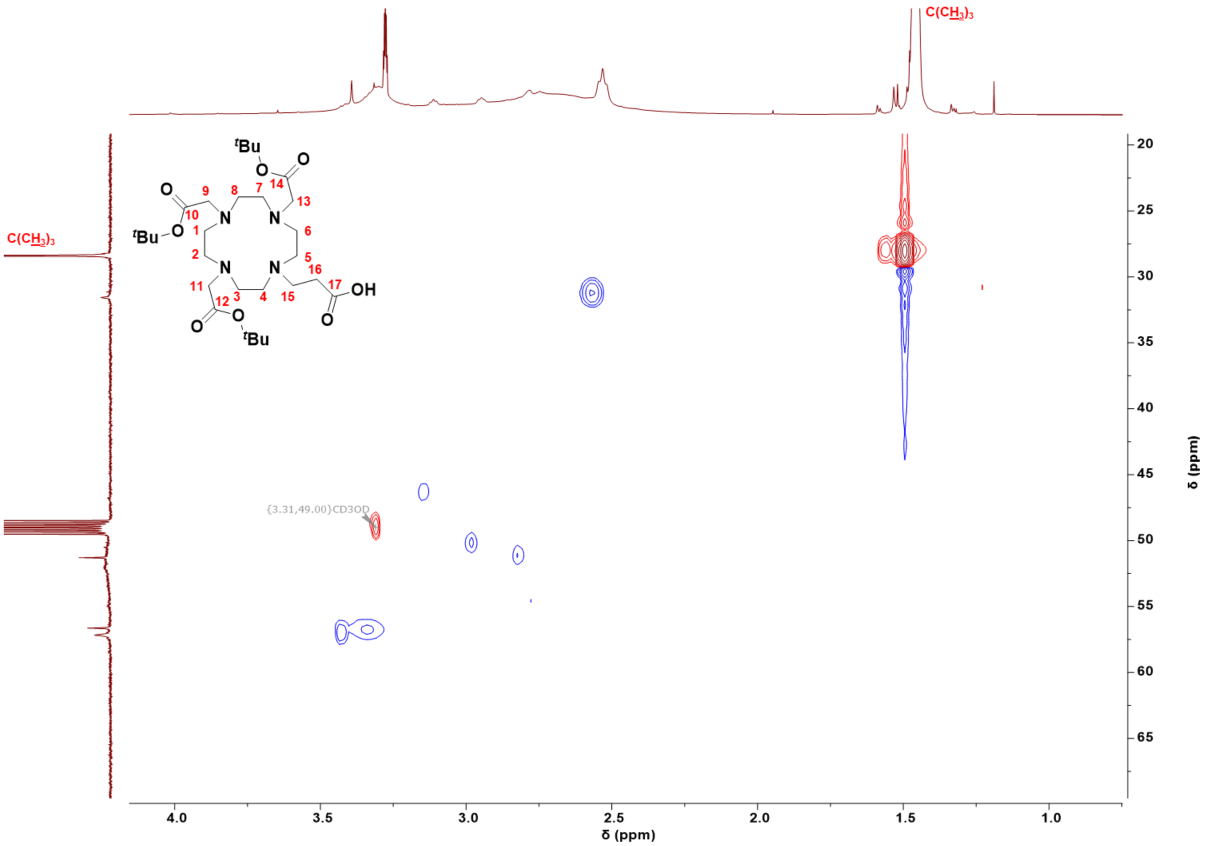

Figure S9. ^{1}H-^{13}C HSQC Spectrum of **2** in CD_3OD at 500 MHz.

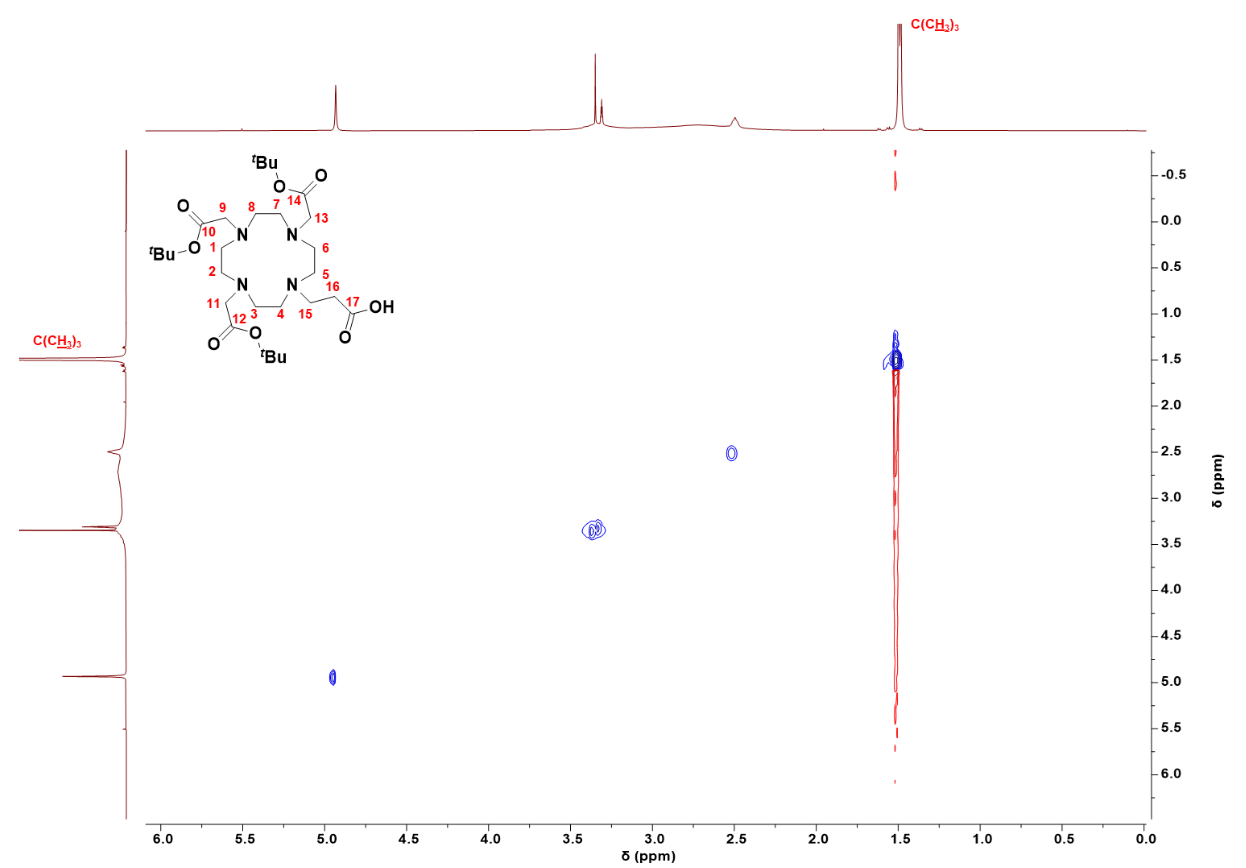

Figure S10. ^1H-^1H TOCSY Spectrum of **2** in CD_3OD at 500 MHz.

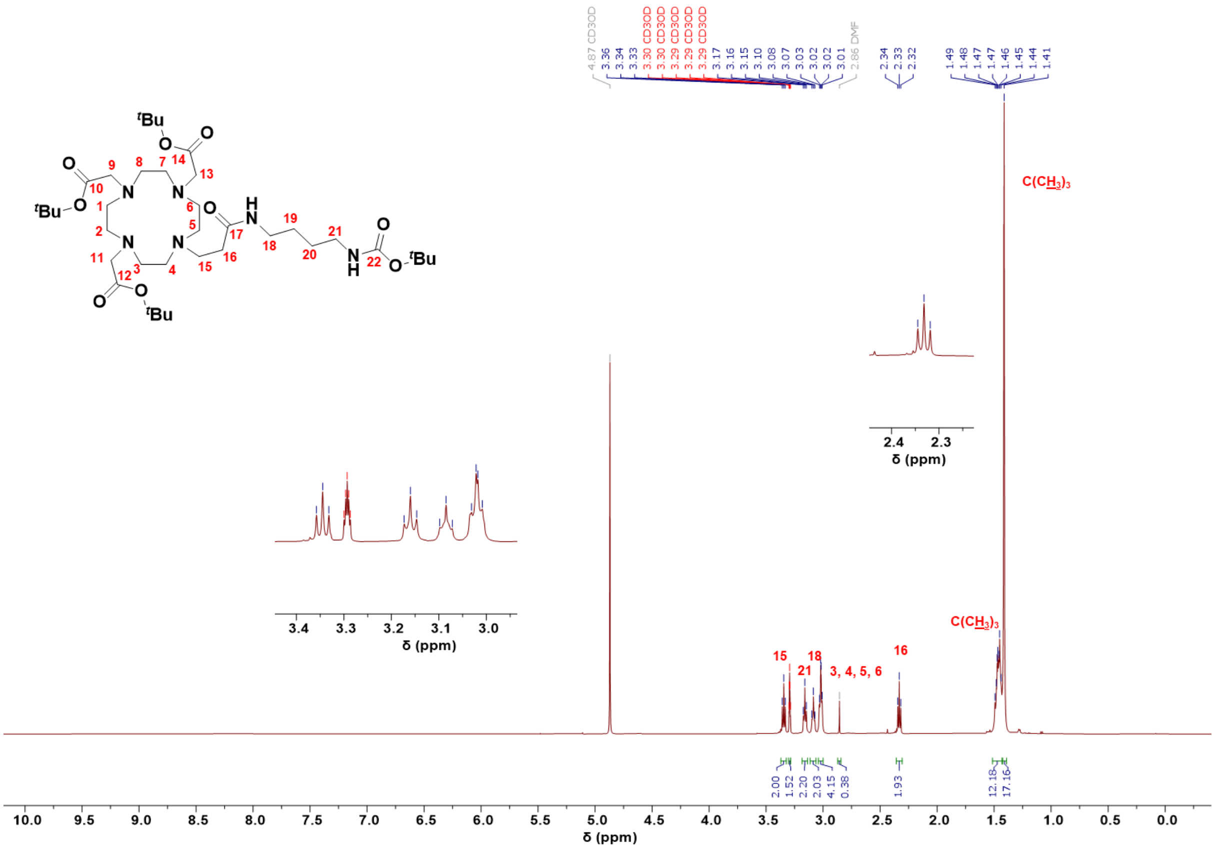

Figure S11. [1]H Spectrum of **3** in CD_3OD at 500 MHz.

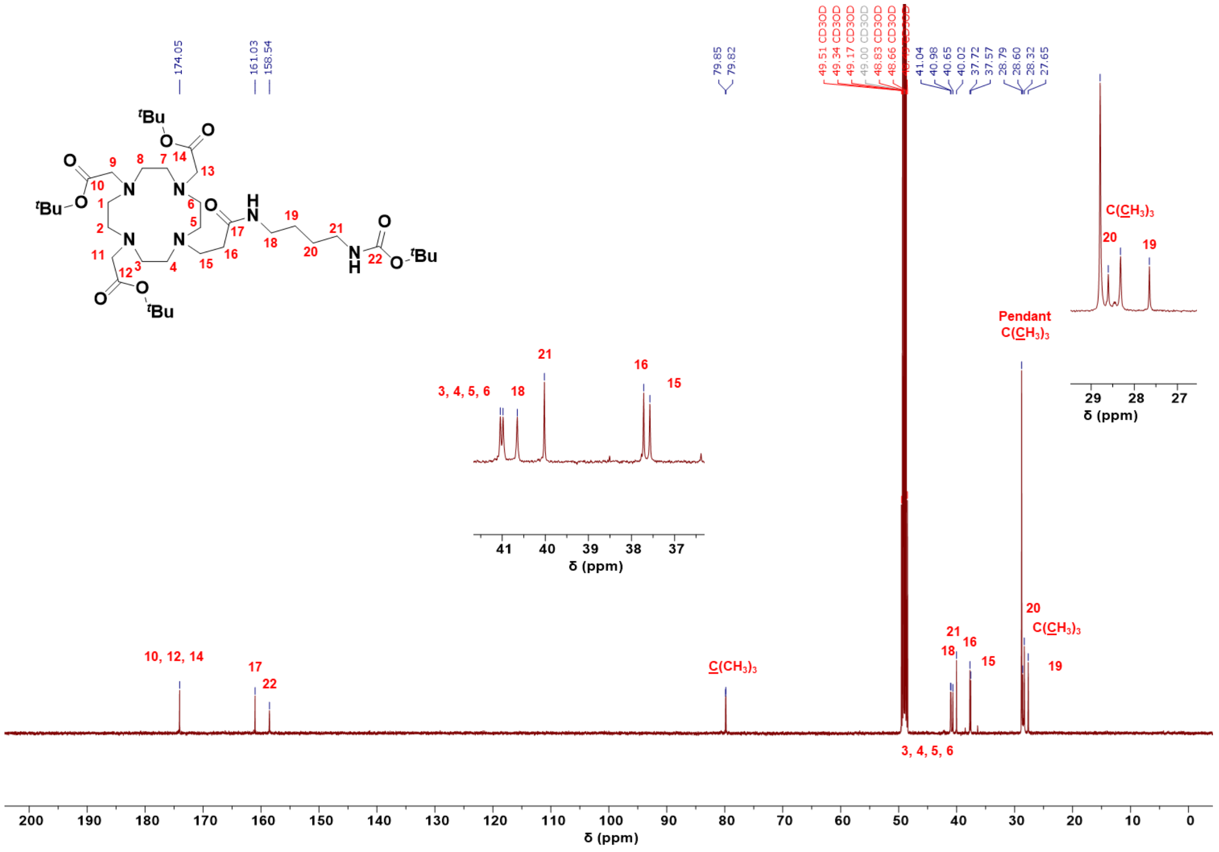

Figure S12. ^{13}C Spectrum of **3** in CD_3OD at 500 MHz.

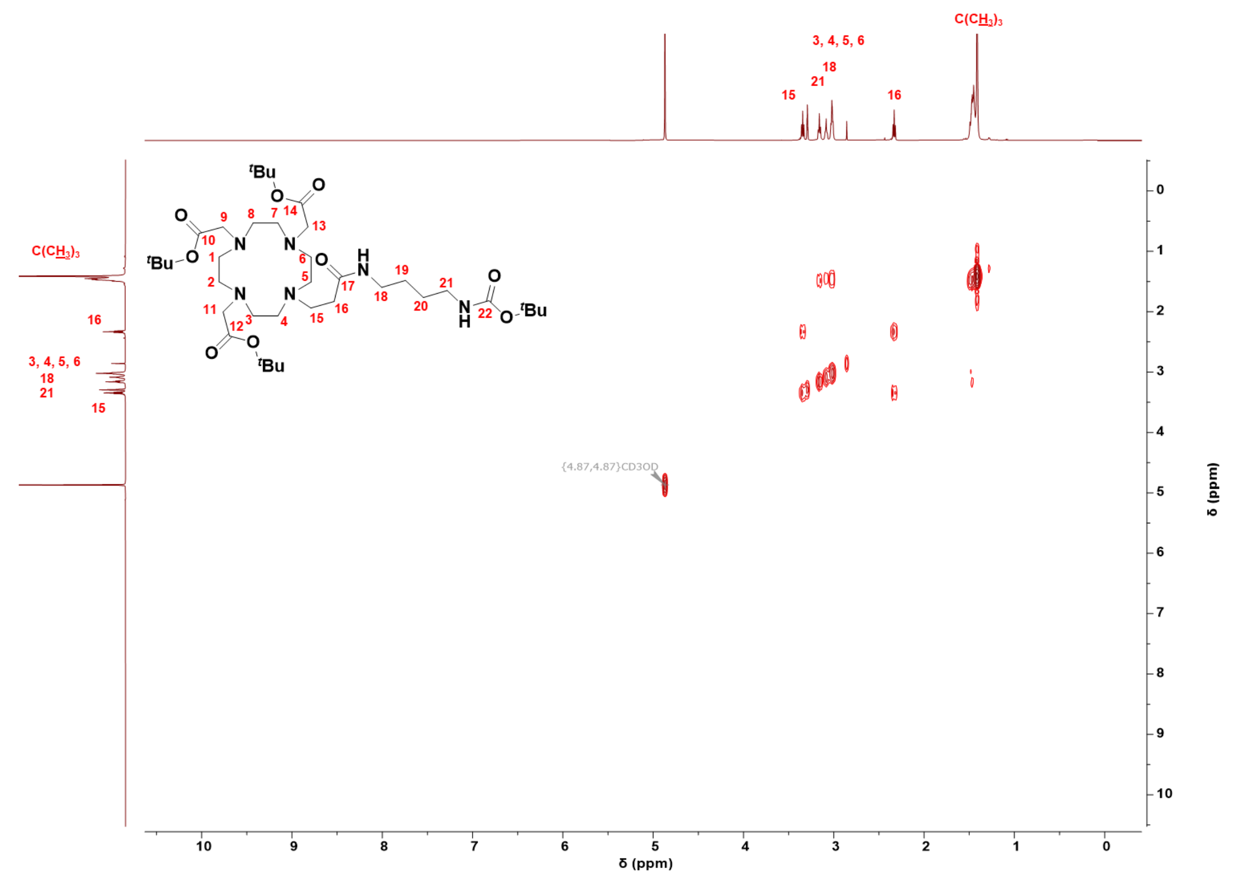

Figure S13. ^{1}H-^{1}H COSY Spectrum of **3** in CD_3OD at 500 MHz.

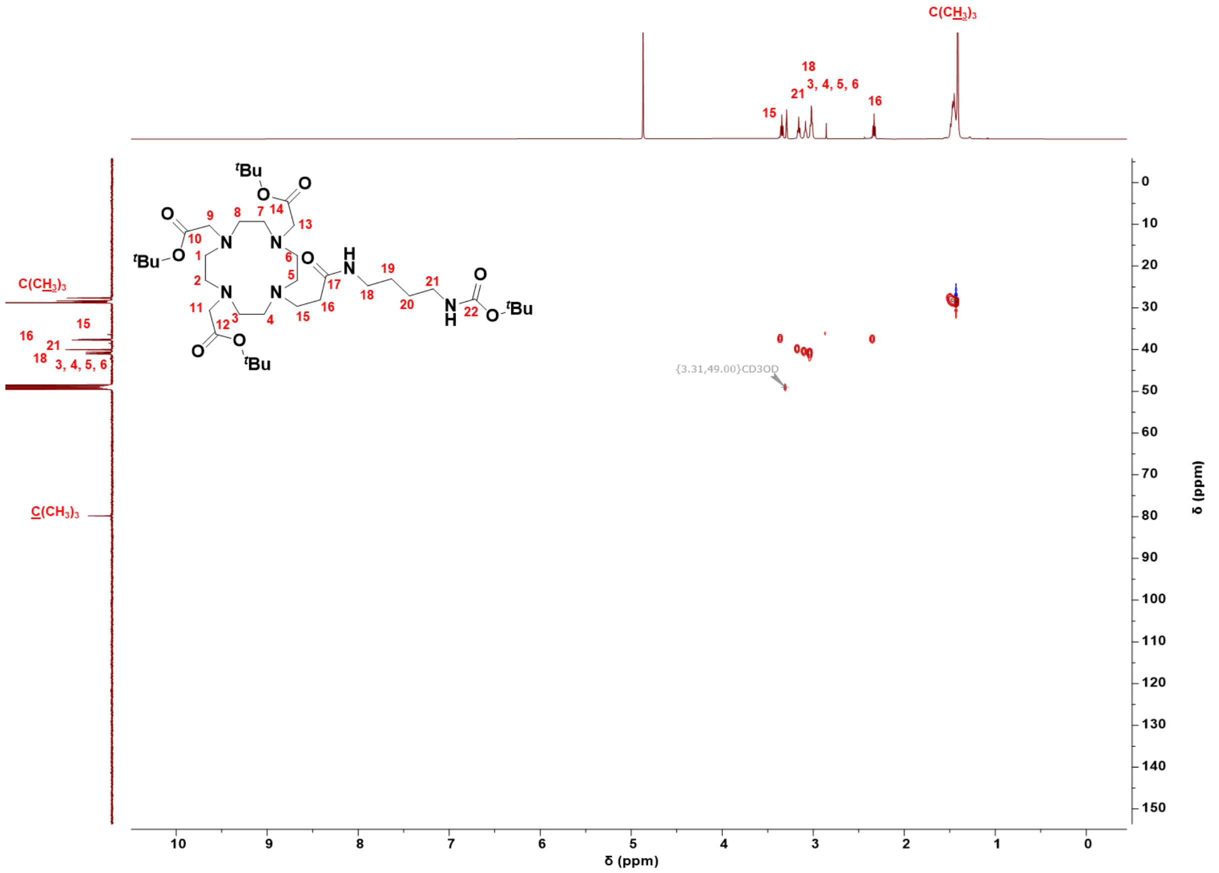

Figure S14. ^{1}H-^{13}C HSQC Spectrum of **3** in CD_3OD at 500 MHz.

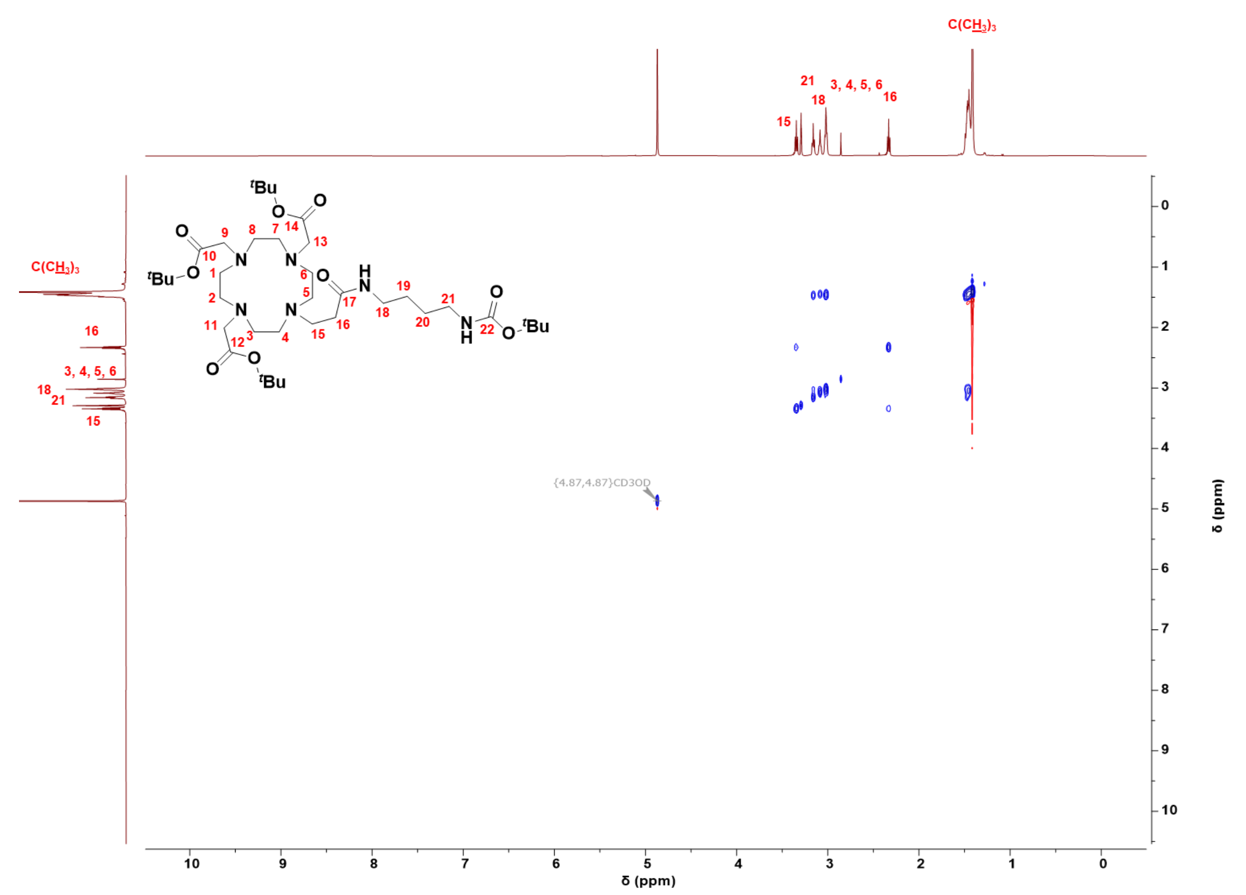

Figure S15. ^{1}H-^{1}H TOCSY Spectrum of **3** in CD_3OD at 500 MHz.

4. MS Spectra of **Gd-C4-IA** Synthons

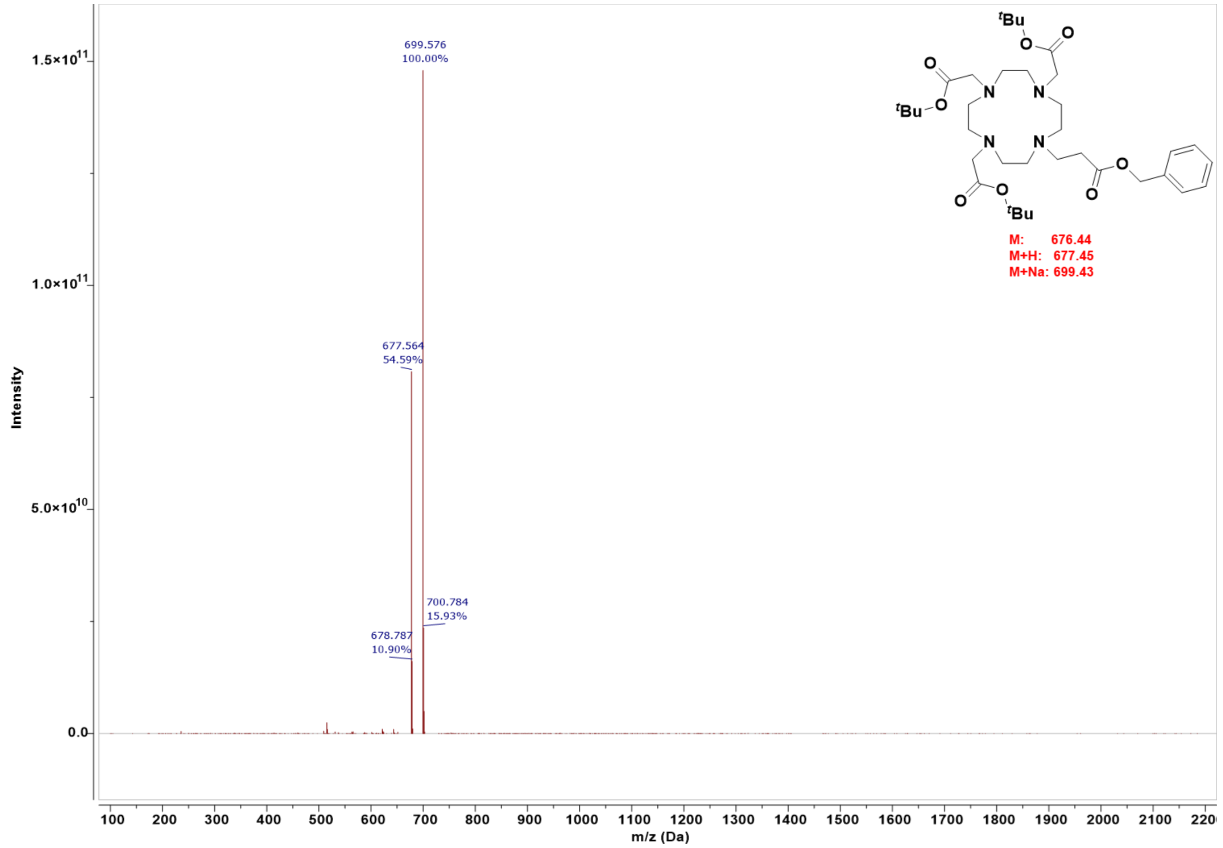

Figure S16. ESI-MS+ Spectrum of **1**.

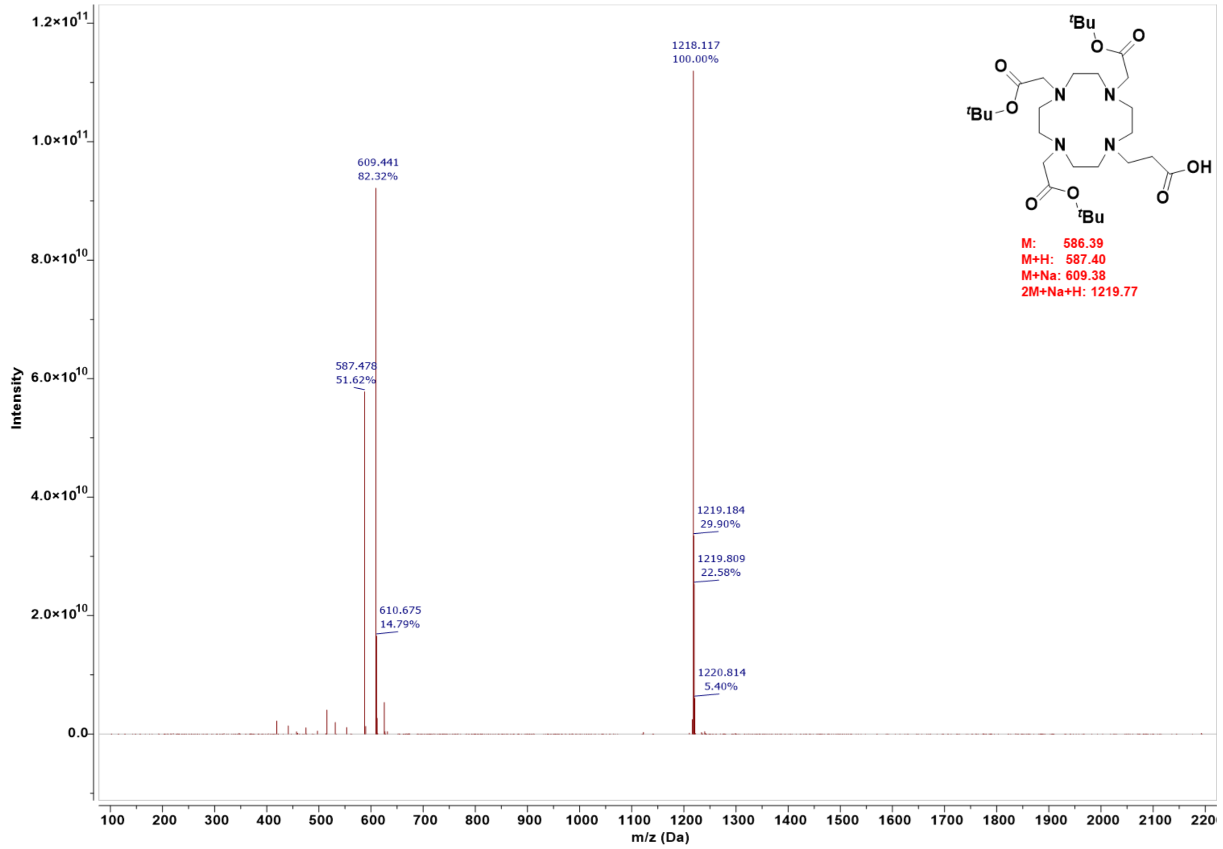

Figure S17. ESI-MS+ Spectrum of **2**.

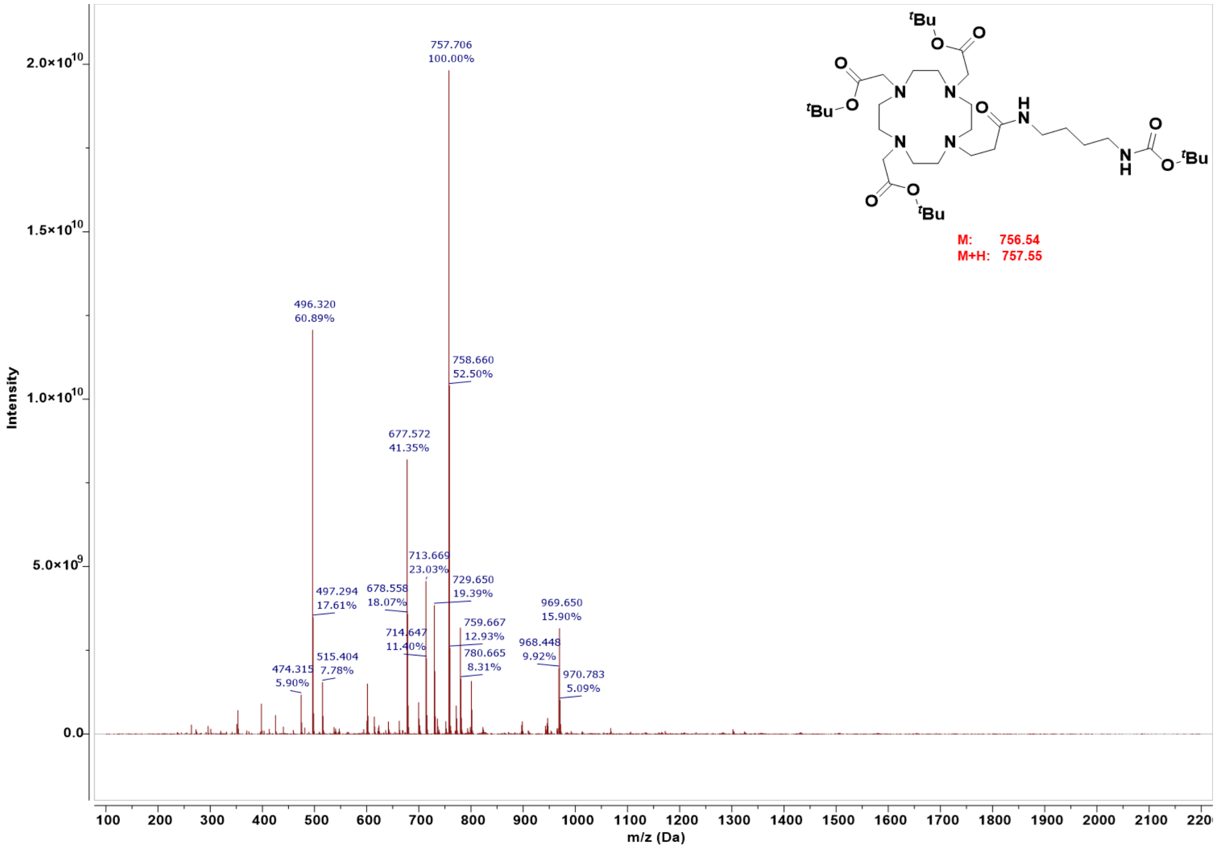

Figure S18. ESI-MS$^+$ Spectrum of **3**.

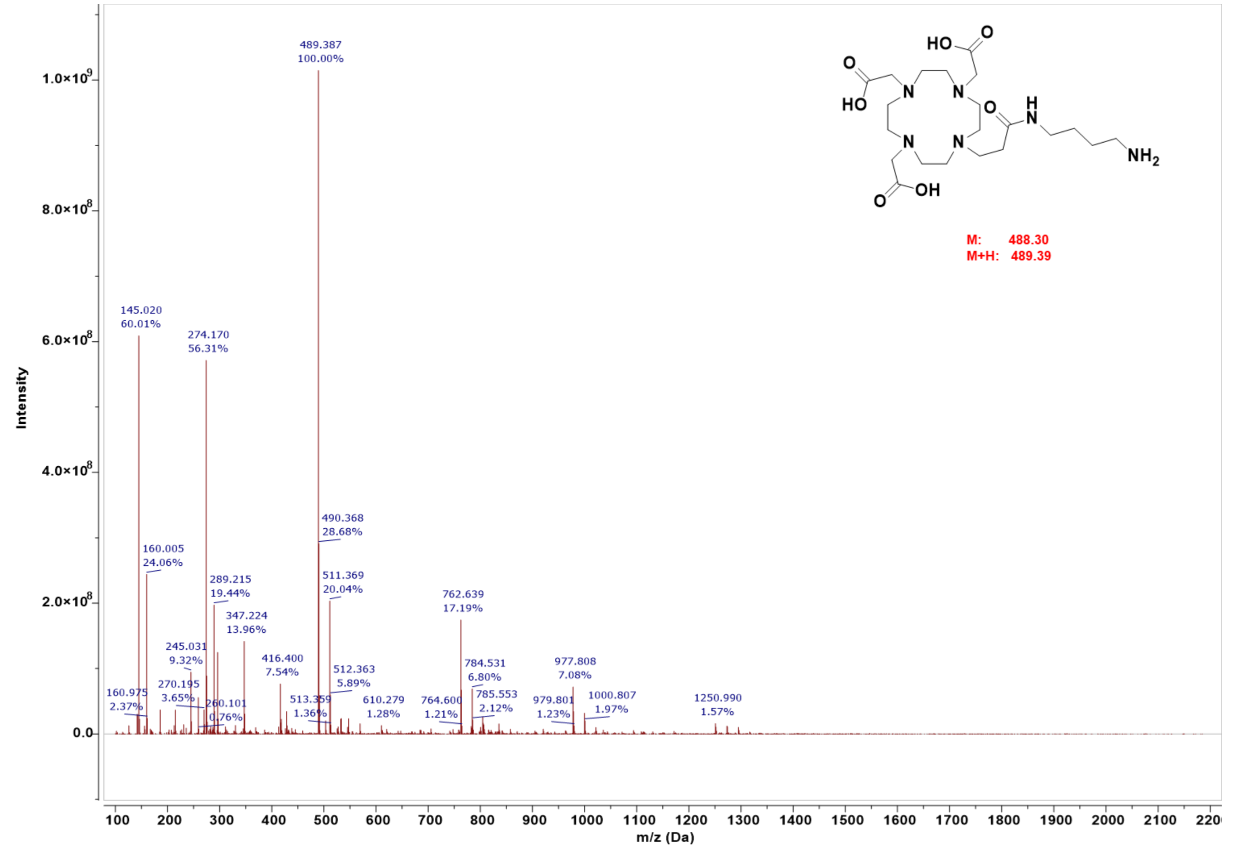

Figure S19. ESI-MS+ Spectrum of crude **4**.

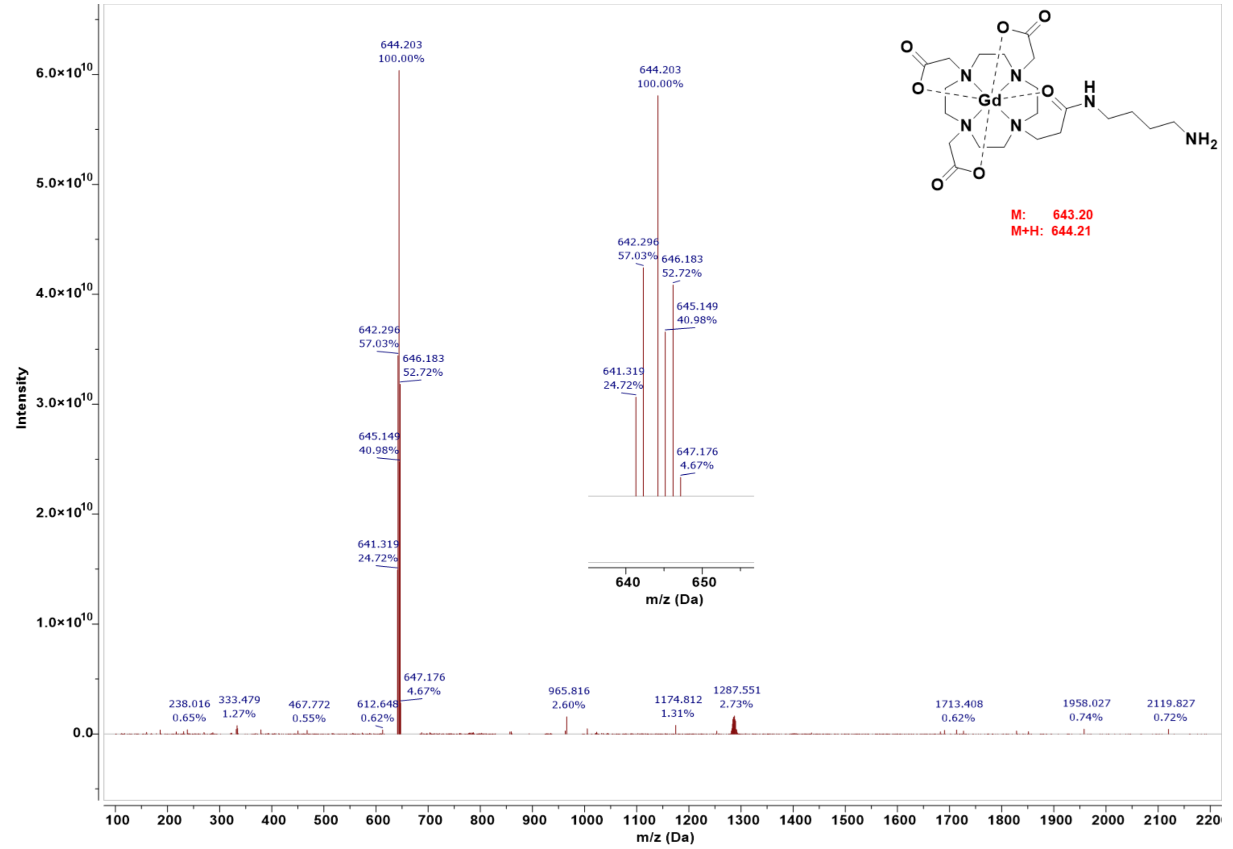

Figure S20. ESI-MS+ Spectrum of **Gd-C4-NH$_2$**.

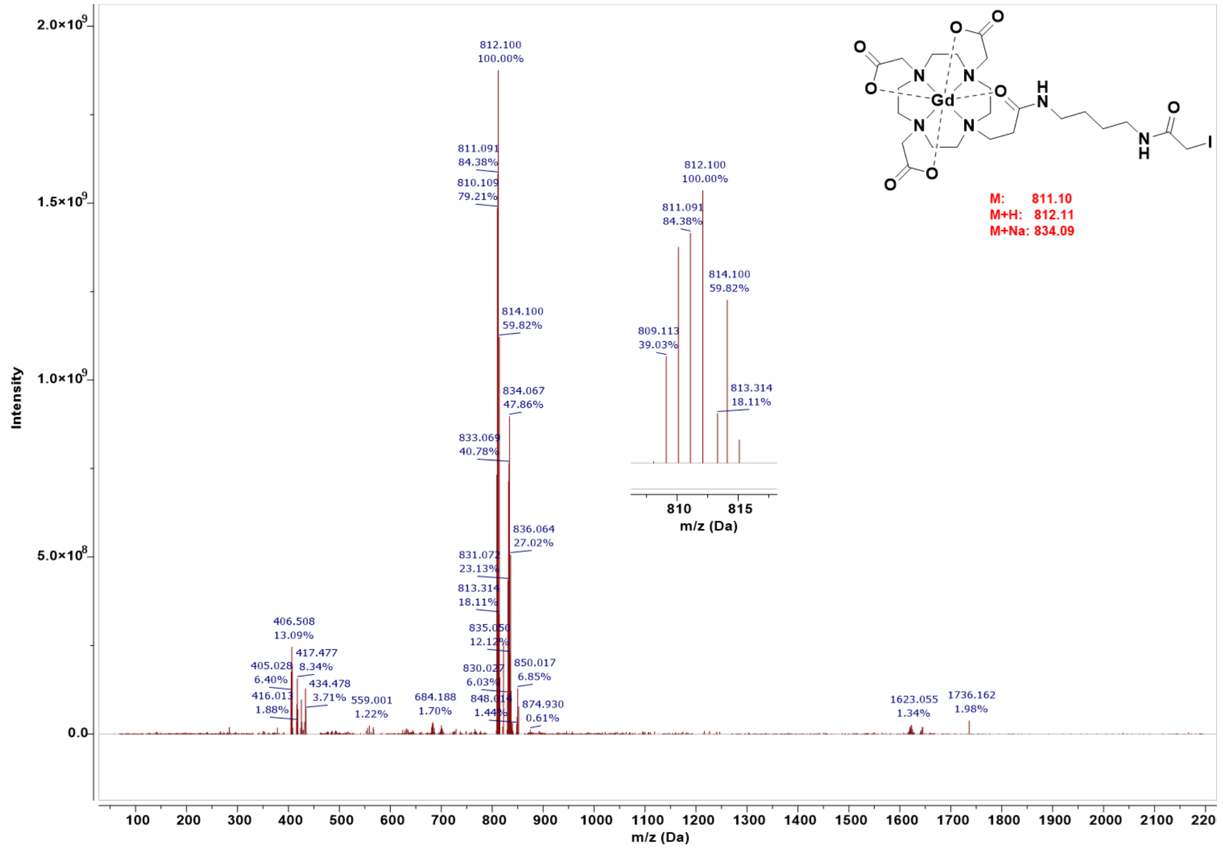

Figure S21. ESI-MS+ Spectrum of **Gd-C4-IA**.

5. HR-MS Spectrum of **Gd-C4-IA**

Samples were prepared at 1 mg/mL in MQ H_2O, and were analyzed using an Agilent 6230 Time of Flight (TOF) mass spectrometer with an electrospray ionization (ESI) source, attached to an Agilent 1200 series HPLC stack. Data was acquired on Agilent Mass Hunter Acquisition software and analyzed on Agilent Mass Hunter Qualitative Analysis software.

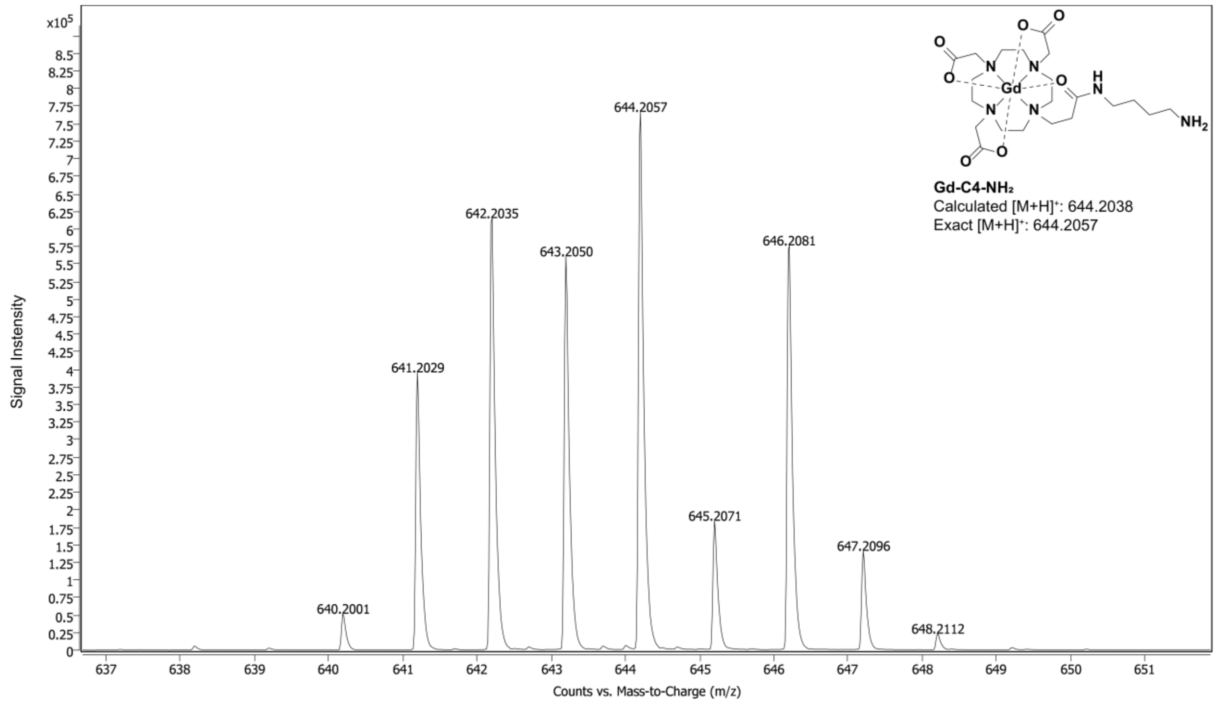

Figure S22. HR-MS Spectrum of **Gd-C4-NH$_2$**.

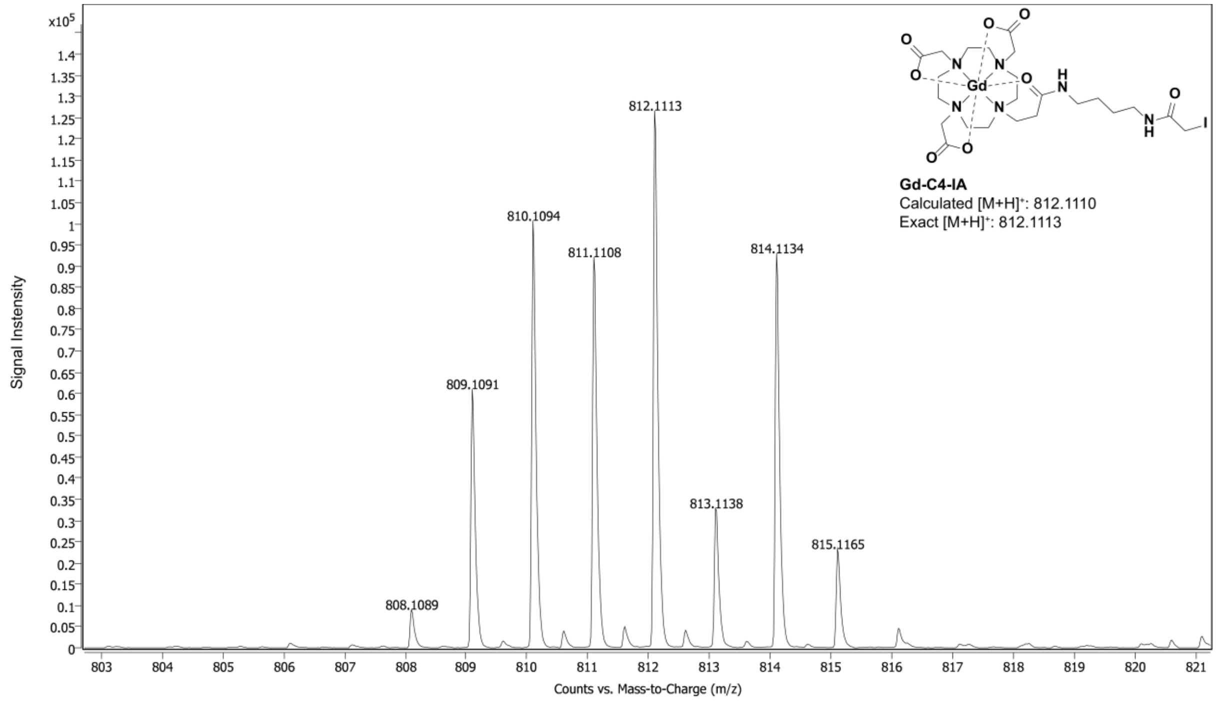

Figure S23. HR-MS Spectrum of **Gd-C4-IA**.

6. HPLC Traces of Gd(III) Complexes

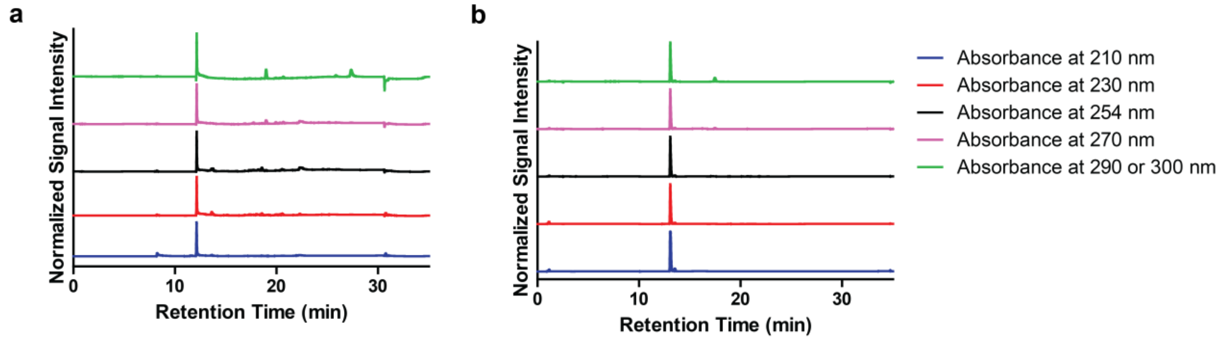

Figure S24. Analytical HPLC chromatogram traces of Gd(III) complexes. (a) **Gd-C4-NH$_2$** and (b) **Gd-C4-IA** HPLC chromatogram traces at 210 nm (blue), 230 nm (red), 254 nm (black), 270 nm (purple), and either 290 nm (b, green) or 300 nm (a, green).

7. Preparation and Characterization of Protein Cages

Protein sequences

Molecular weights (MW), isoelectropoints (pI), and extinction coefficients at 280 nm (ε_{280}, $M^{-1}cm^{-1}$) of proteins were calculated using the SIB Bioinformatics Resource Portal tool (http://web.expasy.org/protparam/).

AaLS-13 (162 residues, MW = 17,684.01 Da, pI = 4.96, ε_{280} = 13,980 $M^{-1}cm^{-1}$)

MEIYEGKLTAEGLRFGIVASRFNHALVGRLVEGAIDCIVRHGGREEDITLVCVPGSWEIPVAAGE
LARKEDIDAVIAIGVLIEGAEPHFDYIASEVSKGLANLSLELRKPISFGDITDDELEEAIECAGTEH
GNKGWEAALSAIEMANLFKSLRLEHHHHHH

OP (192 residues, MW = 20,107.35 Da, pI = 8.67, ε_{280} = 9,970 $M^{-1}cm^{-1}$)

MSQAIGILELRSIAAGMELGDAMLKSANVDLLVSKTISRGKFLLMLGGDIGAIQQAIETGTSQAG
RLLVDSLVLANIHPSVLPAISGLNSVDKRQAVGIVETRSVAACISAADRAVKGSNVTLVRVHMAR
GIGGKCYMVVAGDVSDVALAVTVASSSAGAYGRLVYASLIPRPHEAMWRQMVEGLEHHHHHH

OP-1$_{int}$C (192 residues, MW = 20,123.41 Da, pI = 8.53, ε_{280} = 9,970 $M^{-1}cm^{-1}$)

MSQAIGILELRSIAAGMELGDAMLKSANVDLLVSKTICRGKFLLMLGGDIGAIQQAIETGTSQAG
RLLVDSLVLANIHPSVLPAISGLNSVDKRQAVGIVETRSVAACISAADRAVKGSNVTLVRVHMAR
GIGGKCYMVVAGDVSDVALAVTVASSSAGAYGRLVYASLIPRPHEAMWRQMVEGLEHHHHHH

OP-2$_{int}$C (192 residues, MW = 20,070.36 Da, pI = 7.78, ε_{280} = 9,970 $M^{-1}cm^{-1}$)

MSQAIGILELRSIAAGMELGDAMLKSANVDLLVSKTICRGKFLLMLGGDIGAIQQAIETGTSQAG
RLLVDSLVLANIHPSVLPAISGLNSVDKRQAVGIVETCSVAACISAADRAVKGSNVTLVRVHMAR
GIGGKCYMVVAGDVSDVALAVTVASSSAGAYGRLVYASLIPRPHEAMWRQMVEGLEHHHHHH

OP-3$_{int}$C (192 residues, MW = 20,017.31 Da, pI = 7.11, ε_{280} = 9,970 $M^{-1}cm^{-1}$)

MSQAIGILELRSIAAGMELGDAMLKSANVDLLVSKTICRGKFLLMLGGDIGAIQQAIETGTSQAG
CLLVDSLVLANIHPSVLPAISGLNSVDKRQAVGIVETCSVAACISAADRAVKGSNVTLVRVHMAR
GIGGKCYMVVAGDVSDVALAVTVASSSAGAYGRLVYASLIPRPHEAMWRQMVEGLEHHHHHH

OP-1$_{ext}$C (192 residues, MW = 20,082.32 Da, pI = 7.84, ε_{280} = 9,970 $M^{-1}cm^{-1}$)

MSQAIGILELRSIAAGMELGDAMLKSANVDLLVSKTISRGKFLLMLGGDIGAIQQAIETGTSQAG
RLLVDSLVLANIHPSVLPAISGLNSVDCRQAVGIVETRSVAACISAADRAVKGSNVTLVRVHMAR
GIGGKCYMVVAGDVSDVALAVTVASSSAGAYGRLVYASLIPRPHEAMWRQMVEGLEHHHHHH

Cloning of OP variants

To provide specific handles for conjugation, one, two or three cysteine mutations were introduced per OP protein monomer, affording variants OP-1_{int}C, OP-2_{int}C, and OP-3_{int}C, respectively. The residues targeted from mutation were Ser38, Arg66 and Arg103. The variant OP-1_{int}C, which contains the S38C mutation, has been previously described.[4] As such, plasmid pET29b(+)_OPS38C was used as a basis for generation of the OP-2_{int}C (S38C, R103C), and OP-3_{int}C (S38C, R66C, R103C) variants. The genes for these variants were generated by "QuikChange" (Agilent) site-directed mutagenesis. The primers used for OP-2_{int}C were: OP_R103Cfw; GTATTGTGGAAACCTGTAGCGTGGCGGCG and OP_R103Crv; CGCCGCCACGCTACAGGTTTCCACAATAC, affording plasmid pET29b(+)_OP-2_{int}C, which was used for the next mutagenesis step with primers OP_R66Cfw; AGCCAGGCGGGTTGTCTGCTGGTGG and OP_R66Crv; CCACCAGCAGACAACCCGCCTGGCT. To provide a specific handle for external surface conjugation, a single cysteine mutation was introduced at residue Lys93 to generate the variant OP-1_{ext}C. Using the previously reported pET29b(+)_OP plasmid[5] as a starting point, primers OP_K93Cfw; GGTCTGAATAGCGTGGATTGCCGTCAGGCGGTGGGTATTG, and OP_K93Crv; CAATACCCACCGCCTGACGGCAATCCACGCTATTCAGACC were used for the QuikChange mutagenesis to afford plasmid pET29b(+)_OP. Successful molecular cloning was confirmed by Sanger sequencing (Microsynth AG, Switzerland) of the pET29b(+) plasmids used for protein expression.

Protein expression of AaLS-13 and OP cysteine mutants

AaLS-13 and OP cages were expressed in *E. coli* strain BL21-Gold (DE3) which was transformed with either pMG211-AaLS-13 or the appropriate pET29b(+)-OP. Cells were grown at 37 °C in selective LB medium until the OD_{600} reached ~0.6-0.8, at which point protein production was induced by adding IPTG to a final concentration of 0.1 mM. After culturing at 25 °C for 22 hours, cells were harvested by centrifugation at 5,000 *g* and 4 °C for 10 min. The cell pellet was stored at -20 °C until purification.

Cell lysis and Ni-NTA purification of AaLS-13

The cell pellet from a 400 mL culture was re-suspended in 20 mL lysis buffer (50 mM sodium phosphate (pH 8.0), 300 mM NaCl, 10 mM imidazole) supplemented with lysozyme (0.1 mg/mL), DNase I (5 µg/mL), RNase A (5 µg/mL), and a protease inhibitor cocktail (Sigma). The lysate was incubated for 1 hour at room temperature. After lysis by sonication (using a 50% duty cycle and 80% amplitude setting on ice for 2 min, followed by cooling on ice for 2 min, repeated 5 times) and clearance by centrifugation at 9,500 *g* and 25 ˚C for 25 min, the supernatants were loaded

onto 1.5 mL Ni(II)-NTA Sepharose resin (50% v/v, Qiagen) pre-equilibrated with 30 mL lysis buffer in a gravity flow column. After washing with 40 mL wash buffer (50 mM sodium phosphate (pH 8.0), 800 mM NaCl) containing 20 mM and 40 mM imidazole, AaLS-13 was eluted with 15 mL lysis buffer containing 500 mM imidazole. The buffer was exchanged to AaLS storage buffer (50 mM sodium phosphate buffer (pH 8.0), 200 mM NaCl, 5 mM EDTA) using an Amicon Ultra-15 centrifugal filter unit (30 kDa MWCO) (Merck Millipore). After the buffer was exchanged, a 5 M NaCl solution was added (10% by volume) to the concentrated AaLS-13 sample to give a final NaCl concentration of 600 mM, the mixture was incubated at room temperature for 3 days to complete cage formation. The AaLS-13 cages were then purified by size-exclusion chromatography (SEC) using a Superose 6 increase column (GE Healthcare). The purified AaLS-13 cages were stored at room temperature. The concentration of AaLS-13 was determined by absorbance at 280 nm (ε_{280} = 13,980 $M^{-1}cm^{-1}$).

Cell lysis and Ni-NTA purification of OP variants

Each cell pellet from 800 mL of culture was resuspended in 10 mL of lysis buffer (50 mM sodium phosphate buffer (pH 7.4), 1 M NaCl, 20 mM imidazole) supplemented with lysozyme (0.1 mg/mL), DNase I (5 µg/mL), RNase A (5 µg/mL), 2 mM DTT, and protease inhibitor cocktail (Sigma), and incubated at 37 °C for 1.5 h. After lysis, sonication and centrifugation (10,000 *g*) at 25 °C for 25 min, the supernatant was loaded onto 5 mL of Ni-NTA resin in a gravity flow column and incubated for 15 min. After multiple washes with lysis buffer containing 20 mM and 40 mM imidazole, the target protein was eluted with elution buffer (50 mM sodium phosphate buffer (pH 7.4), 300 mM NaCl, 500 mM imidazole). Typically, between 10-20 mL were collected and supplemented with 2 U/mL RNase A, 5 mM EDTA and protease inhibitor cocktail and incubated overnight at 37 °C to digest any contaminant *E. coli* RNA which was not removed during the Ni-NTA purification. The protein was then purified by SEC using a Superose 6 increase column. After this point, the storage of the protein and all experiments were carried out at room temperature unless specified otherwise. The concentration of OP-3_{int}C was determined by absorbance at 280 nm (ε_{280} = 9,970 $M^{-1}cm^{-1}$).

Sulfhydryl groups on protein cages

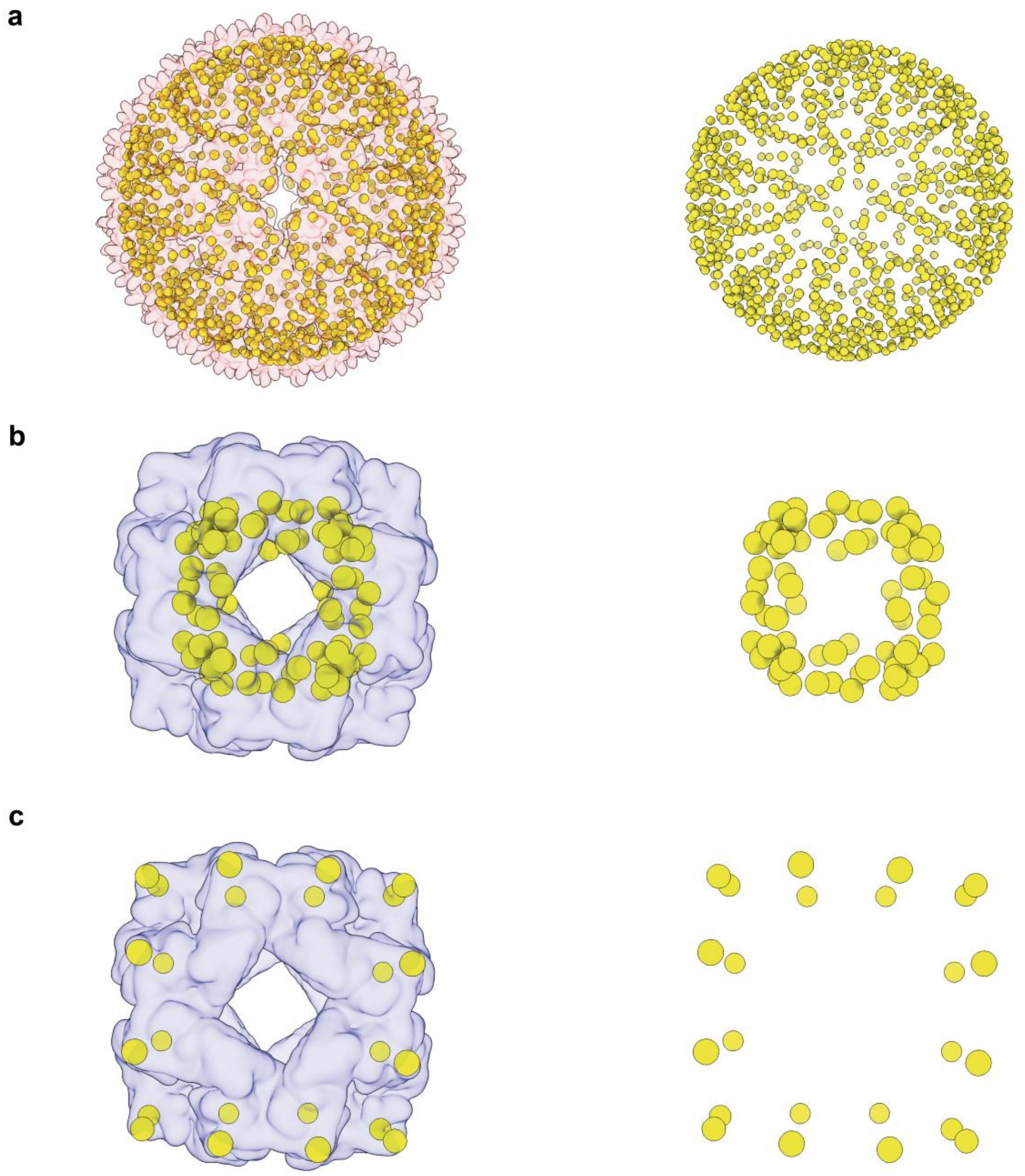

Figure S25. Position of the cysteine residues on AaLS-13 and OP. Cysteines residues for each of the cage variants, (a) AaLS-13 (Cys127), (b) OP-3_{int}C (Cys38, Cys66, and Cys103), and (c) OP-1_{ext}C (Cys93) are highlighted as yellow spheres, with (left) or without (right) surface representation.

Selected residues in close proximity to reactive sites

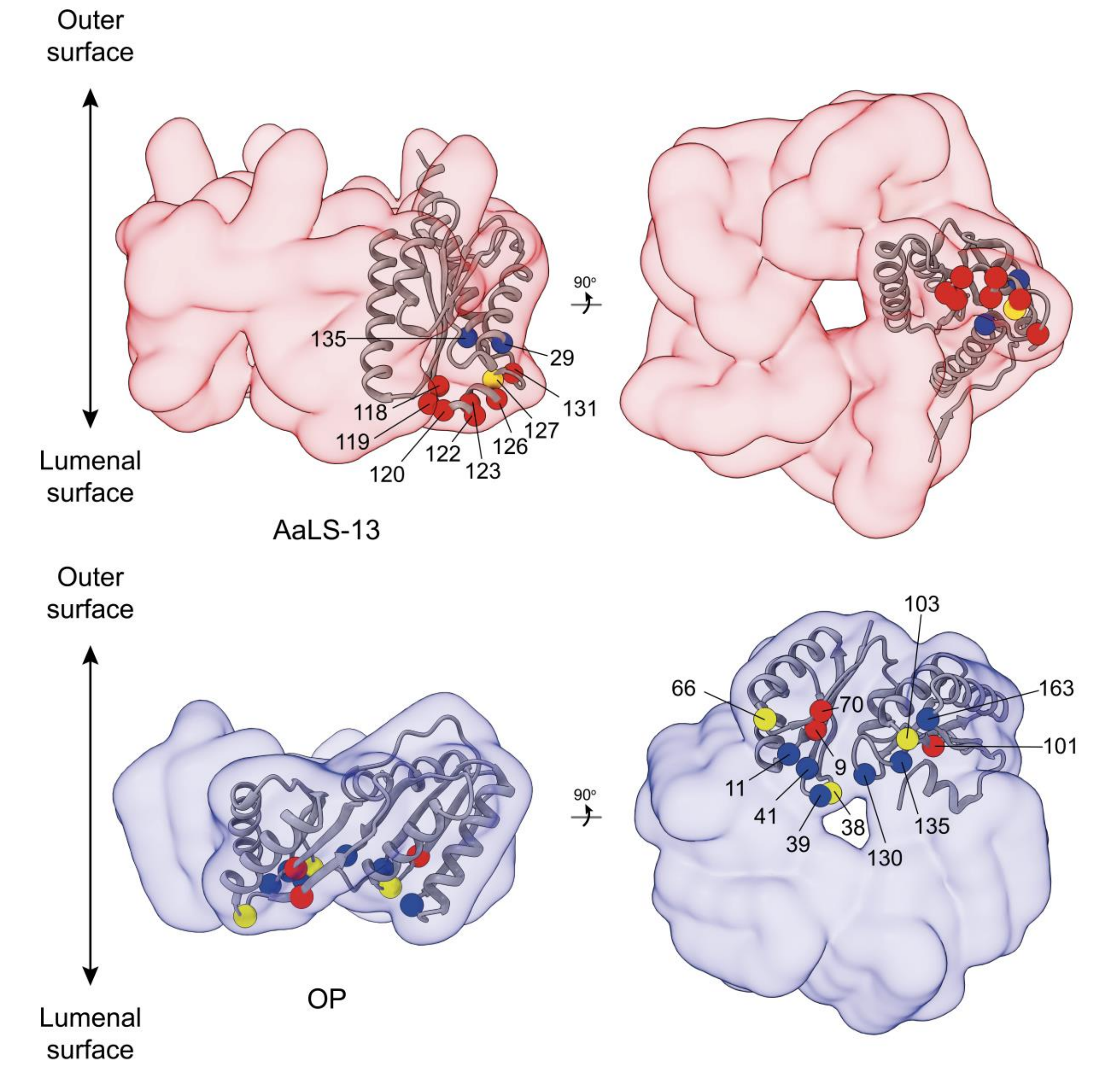

Figure S26. Charged residues in close proximity to the reactive sites. Transparent surface of a pentamer used to construct an AaLS-13 cage (top) and a trimer used to construct OP (bottom). Monomers are shown as gray ribbons. Negatively charged residues close to Cys127 on AaLS-13 (yellow sphere) are shown as red spheres (Asp118, Asp119, Glu122, Glu123, Glu126, and Glu131), while positively charged residues are shown as blue spheres (Arg29 and Lys135). Positively and negatively charged residues near the positions targeted for cysteine mutations (Ser38, Arg66, and Arg103, represented as yellow spheres) on OP are shown as blue spheres (Arg11, Arg39, Arg130, Lys135, Arg163) and red spheres (Glu9, Asp70, and Glu101).

8. Protein Conjugation with **Gd-C4-IA**

Protein labeling with Gd-C4-IA

AaLS-13 (820 μM) was mixed with 5 equivalents of **Gd-C4-IA** per monomer in a final volume of 500 μL. OP constructs (520 μM) were mixed with 4 equivalents of **Gd-C4-IA** per reactive cysteine residue, also in a final volume of 500 μL. After 4.5 h incubation at room temperature in the dark, the samples were loaded onto a PD-10 minitrap desalting column to remove unbound Gd complexes. The samples were subjected to a further round of purification by size-exclusion chromatography using a Superose 6 increase column, pre-equilibrated in the appropriate protein storage buffer: 50 mM sodium phosphate (pH 8.0), 200 mM NaCl, 5 mM EDTA for AaLS-13 and 25 mM Tris-HCl (pH 7.6), 200 mM NaCl, 5 mM EDTA for OP cages.

Size exclusion chromatography of Gd-protein conjugates

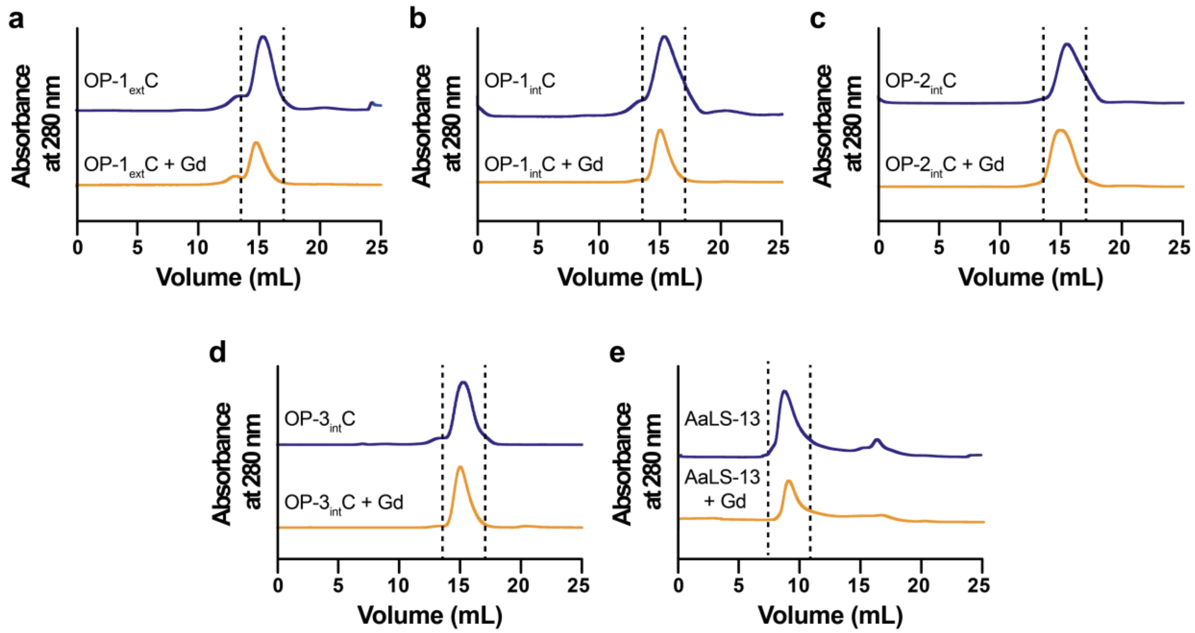

Figure S27. Size-exclusion chromatography (SEC) of AaLS-13 and OP cages. SEC chromatograms of the crude mixtures after Ni-NTA affinity purification are shown (top blue curves) for (a) OP-1extC, (b) OP-1intC, (c) OP-2intC, (d) OP-3intC, and (e) AaLS-13. The elution traces of Gd-labeled cages are shown in orange (bottom curves).

MS Spectra of Gd-Protein Conjugates

The stability of Gd(III)-labeled protein cages was evaluated by MS. AaLS-13 as studied in 50 mM sodium phosphate (pH 8.0), 200 mM NaCl, 5 mM EDTA; while the OP cages were studied in 25 mM Tris (pH 7.6), 200 mM NaCl, 5 mM EDTA. MS samples were prepared at 1 mg/mL in appropriate buffers. After the first MS, AaLS-13 and OP-3intC were left in their respective buffers at room temperature at 50 µM for 4 months and 500 µM for 7 months, respectively, and MS spectra prepared again. Ions were detected by Bruker Impact II o-TOF High Resolution Time of Flight Mass Spectrometer connected to a Bruker Elute UHPLC. Samples were injected onto a Waters Acquity UPLC Protein BEH C4 column (300 A, 1.7 µM, 2.1 mm x 50 mm) using a gradient separation flowing at 0.3 mL/min of Water with 0.1% formic acid (A) and acetonitrile with 0.1% formic acid (B) programmed as follows: 0 minutes 95%A:5%B to 10 minutes 5%A:95%B. Two column washes were performed with each injection using the same gradient percentages described above before re-equilibration was performed to eliminate any carryover. Compass HyStar 4.1 Data Acquisition software was used for instrument operation, and Compass DataAnalysis was used for data analysis and processing. All proteins were deconvoluted using MaxEntropy deconvolution.

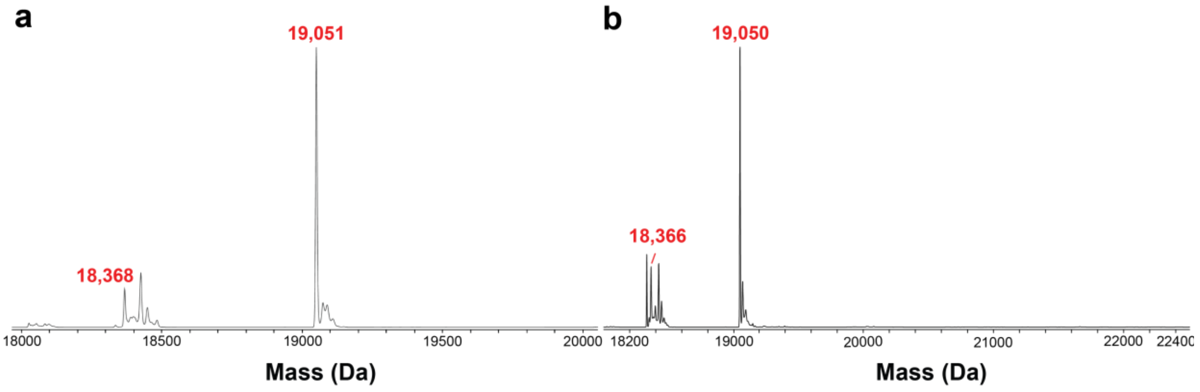

AaLS-13 calc.	17,684.0 Da
Gd-C4-IA	+ 683.0 Da
	18,367.0 Da
Gd-C4-IA	+ 683.0 Da
	19,050.0 Da

Figure S28. MS Traces of Gd-AaLS-13 at (a) 0 and (b) 4 months. Masses correspond to AaLS-13 monomer.

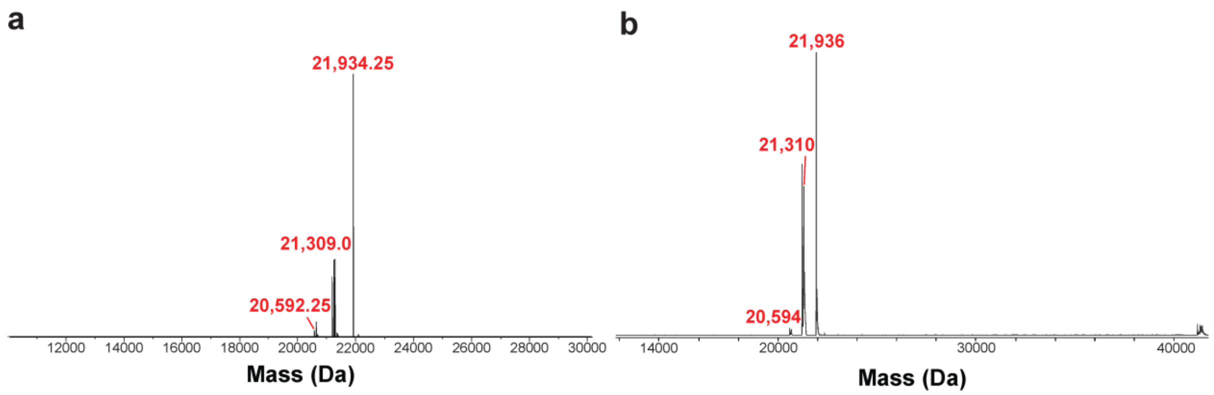

OP-3_{int}C calc.	20,017.0 Da
Met	- 131.2 Da
	19,886.1 Da
Gd-C4-IA	+ 683.0 Da
	20,569.1 Da
Gd-C4-IA	+ 683.0 Da
	21,252.1 Da
Gd-C4	
	21,935.1 Da

Figure S29. MS Traces of Gd-OP-3_{int}C at (a) 0 and (b) 7 months. Masses correspond to OP-3_{int}C monomer.

Analysis of Gd(III) content by ICP-MS

Quantification of Gd in relaxivity samples was accomplished using inductively coupled plasma mass spectrometry (ICP-MS) of acid digested samples. Specifically, 10 μL of each sample was digested in 300 μL concentrated trace nitric acid (> 69%, Thermo Fisher Scientific, Waltham, MA, USA) and placed at 65 °C for at least 3 hours to allow for complete sample digestion. Ultrapure H_2O (18.2 MΩ·cm) was then added to produce a final solution of 3.0% nitric acid in a total sample volume of 10 mL. Quantitative standards were made using a 1,000 μg/mL Gd elemental standard (Inorganic Ventures, Christiansburg, VA, USA) which were used to create a 200 ng Gd / g solvent element standard in 3.0% nitric acid (v/v) in a total sample volume of 50 mL. A second quantitative standard was made by performing a 100x dilution of the 200 ng/g Gd standard, to create a 2 ng/g element standard in 3.0% nitric acid (v/v) in a total sample volume of 50 mL. A solution of 3.0% nitric acid (v/v) was used as the calibration blank.

ICP-MS was performed on a computer-controlled (QTEGRA software) Thermo iCapQ ICP-MS (Thermo Fisher Scientific, Waltham, MA, USA) operating in STD mode and equipped with an ESI SC-2DX PrepFAST autosampler (Omaha, NE, USA). Internal standard was added inline using the prepFAST system and consisted of 1 ng/mL of a mixed element solution containing Li, Sc, Y, In, Tb, Bi (IV-ICPMS-71D from Inorganic Ventures). Online dilution was also carried out by the prepFAST system and used to generate a calibration curve consisting of 200, 100, 50, 10, 2, 1, 0.5, 0.1, 0.01, and 0.01 ppb Gd. Each sample was acquired using 1 survey run (10 sweeps) and 3 main (peak jumping) runs (40 sweeps). The isotopes selected for analysis were ^{156,157}Gd, and ^{115}In, ^{159}Tb (chosen as internal standards for data interpolation and machine stability). Instrument performance is optimized daily through autotuning followed by verification via a performance report (passing manufacturer specifications).

UV-vis spectroscopy of Gd(III) complexes

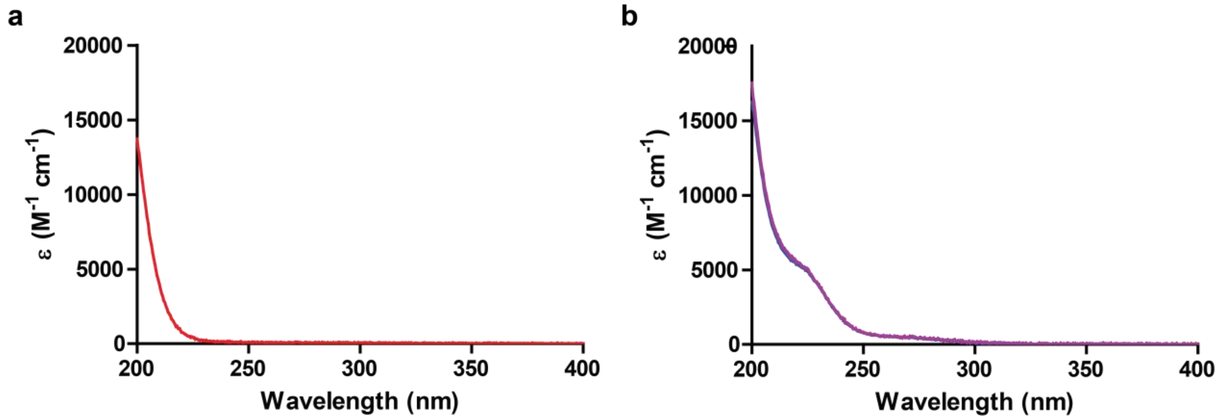

Figure S30. UV-vis spectra of Gd(III) complexes in MQ H_2O. Molar extinction coefficient of (a) **Gd-C4-NH₂** and (b) **Gd-C4-IA**.

Gd-C4-NH_2 and **Gd-C4-IA** were dissolved in MQ H_2O at 1 mM and the UV-vis spectra obtained to confirm the lack of absorption at 280 nm, the wavelength used to quantify protein concentration.

The absorption at 280 nm was measured for Gd-protein conjugates in 50 mM sodium phosphate (pH 8.0), 200 mM NaCl, 5 mM EDTA for AaLS-13 and 25 mM Tris (pH 7.6), 200 mM NaCl, 5 mM EDTA for the OP variants. The molar extinction coefficient at 280 nm is 13,980 M^{-1} cm^{-1} and 10,095 M^{-1} cm^{-1} for AaLS-13 and OP variants, respectively.

Quantification of protein cage labeling with Gd(III)

Loading of Gd-C4-IA was quantified by UV-vis measurement of protein concentration and ICP measurement of Gd concentration. The loading for each sample was measured in triplicate and averaged to give the loading value.

Table S1. Gd-protein Conjugate Labeling Calculation

Batch Number	[Gd] (uM)	[monomer] (uM)	[Cage] (uM)	Avg Gd/cage	Reactive Sites	Total Avg Gd/cage	Labeling Efficiency
**Gd-AaLS-13**[a]							
1	146 ± 2	365 ± 6	1.01	144 ± 2	360	149 ± 12	0.41
2	116 ± 2	310 ± 11	0.860	135 ± 3			
3	207 ± 4	450 ± 10	1.25	166 ± 3			
4	112 ± 5	266 ± 5	0.740	152 ± 7			
**Gd-OP-3$_{int}$C**[b]							
1	368 ± 16	286 ± 7	12.0	31 ± 1	72	33 ± 7	0.46
2	312 ± 11	334 ± 6	13.9	22.4 ± 0.8			
3	360 ± 19	301 ± 11	12.5	29 ± 2			
4	903 ± 5	502	20.9	43.2 ± 0.2			
5	738 ± 7	509 ± 4	21.2	34.8 ± 0.3			
6	300 ± 4	179 ± 4	7.48	40.1 ± 0.5			
**Gd-OP-2$_{int}$C**[b]							
1	335 ± 8	380	15.8	21.8 ± 0.5	48	23 ± 1	0.47
2	349 ± 3	380	15.8	22.0 ± 0.2			
3	466 ± 18	490 ± 4	20.4	22.8 ± 0.9			
4	194 ± 6	190 ± 4	7.93	24.4 ± 0.7			
**Gd-OP-1$_{int}$C**[b]							
1	244 ± 3	459	19.1	12.7 ± 0.1	24	11 ± 1	0.46
2	169 ± 7	402 ± 2	16.7	10.1 ± 0.4			
3	57 ± 1	133 ± 2	5.55	10.2 ± 0.2			
**Gd-OP-1$_{ext}$C**[b]							
1	785 ± 31	1132	47.2	16.6 ± 0.7	24	14 ± 2	0.60
2	652 ± 2	1034 ± 7	43.1	15.13 ± 0.06			
3	101 ± 3	211 ± 2	8.81	11.5 ± 0.4			

[a]Measurements in 50 mM sodium phosphate (pH 8.0), 200 mM NaCl, 5 mM EDTA

[b]Measurements in 25 mM Tris (pH 7.6), 200 mM NaCl, 5 mM EDTA

9. Relaxivity Measurements

Relaxivity measurements at 1.4 T

Gd-AaLS-13 was studied in sodium phosphate buffer (50 mM sodium phosphate (pH 8.0), 200 mM NaCl, 5 mM EDTA) while the OP proteins were studied in Tris buffer (25 mM Tris (pH 7.6), 200 mM NaCl, 5 mM EDTA). **Gd-C4-IA** was studied in sodium phosphate buffer and Tris buffer.

For each material, a stock sample was prepared at the following concentrations: 784 µM **Gd-AaLS-13**, 440 µM **Gd-OP-1$_{int}$C**, 360 µM **Gd-OP-2$_{int}$C**, 453 µM **Gd-OP-3$_{int}$C**, 1169 µM **Gd-OP-1$_{ext}$C**, and 4.5 mM (sodium phosphate buffer) or 3.5 mM (Tris buffer) **Gd-C4-IA**. The stock sample was serially diluted four times, generating 5 samples of 400 µL or 500 µL each (as material allowed), and heated to 37 °C. Relaxation times were measured on a Bruker mq60 NMR analyzer equipped with Minispec v 2.51 Rev.00/NT software (Bruker Biospin, Billerica, MA, USA) operating at 1.41 T (60 MHz) and 37 °C. Measurement of T_1 relaxation times were made using an inversion recovery pulse sequence using the following parameters: 4 scans per point, 10 data points, mono-exponential curve fitting, phase cycling, 10 ms first pulse separation, and a recycle delay and final pulse separation ≥ 5 T_1. Measurement of T_2 relaxation times were made using the Carr-Purcell-Meiboom-Gill (CPMG) pulse sequence using the following parameters: 10 scans per point, 5000 data points, mono-exponential curve fitting, phase cycling, a recycle delay of 10 sec, and τ of 1 sec. The inverse of the relaxation time ($1/T_1$ or $1/T_2$, s^{-1}) was plotted against the Gd(III) concentration (mM) determined by ICP-MS for each of the five samples. By applying a linear fit to this data, the slope generated was defined as the relaxivity (r_1, r_2) of the agent in units of mM^{-1} s^{-1}.

Relaxivity measurements at 7 T

A 60 µL aliquot of each sample from 1.4 T measurements was pipetted into flame sealed Pasteur pipettes. The pipette tips containing solution were scored, separated, and sealed with parafilm to make small capillaries containing solution. These capillaries were imaged using a Bruker PharmaScan 7 T MR imaging spectrometer (Bruker BioSpin, Billerca, MA, USA). T_1 relaxation times were measured using a rapid-acquisition rapid-echo (RARE-VTR) T_1-map pulse sequence with static TE (10 ms) and variable TR (100, 200, 400, 500, 750, 1000, 2500, 7500, 10000 ms) values. Imaging parameters were as follows: field of view, 25 x 25 mm^2; matrix size, 256 x 256; number of axial slices, 5; slice thickness, 1.0 mm; and averages, 4. T_2 relaxation times were measured using a multislice multiecho (MSME) T_2-map pulse sequence, with static TR (5000 ms) and 32 fitted echoes in 11 ms intervals (11, 22, ..., 352 ms). Imaging parameters were as follows: field of view, 25 × 25 mm2; matrix size, 256 × 256; number of axial slices, 4; slice thickness, 1.0

mm; and averages, 3. T_1 and T_2 analysis was carried out using the image sequence analysis tool in Paravision 6.0 software (Bruker) with mono-exponential curve-fitting of image intensities of selected ROIs for each axial slice. The relaxation rates ($1/T_1$ or $1/T_2$, s^{-1}) was plotted against the Gd(III) concentration (mM) determined by ICP-MS for each of the five samples. By applying a linear fit to this data, the generated was defined as the relaxivity (r_1, r_2) of the agent in units of mM^{-1} s^{-1}.

Relaxivity measurements data at 1.4 T and 7 T

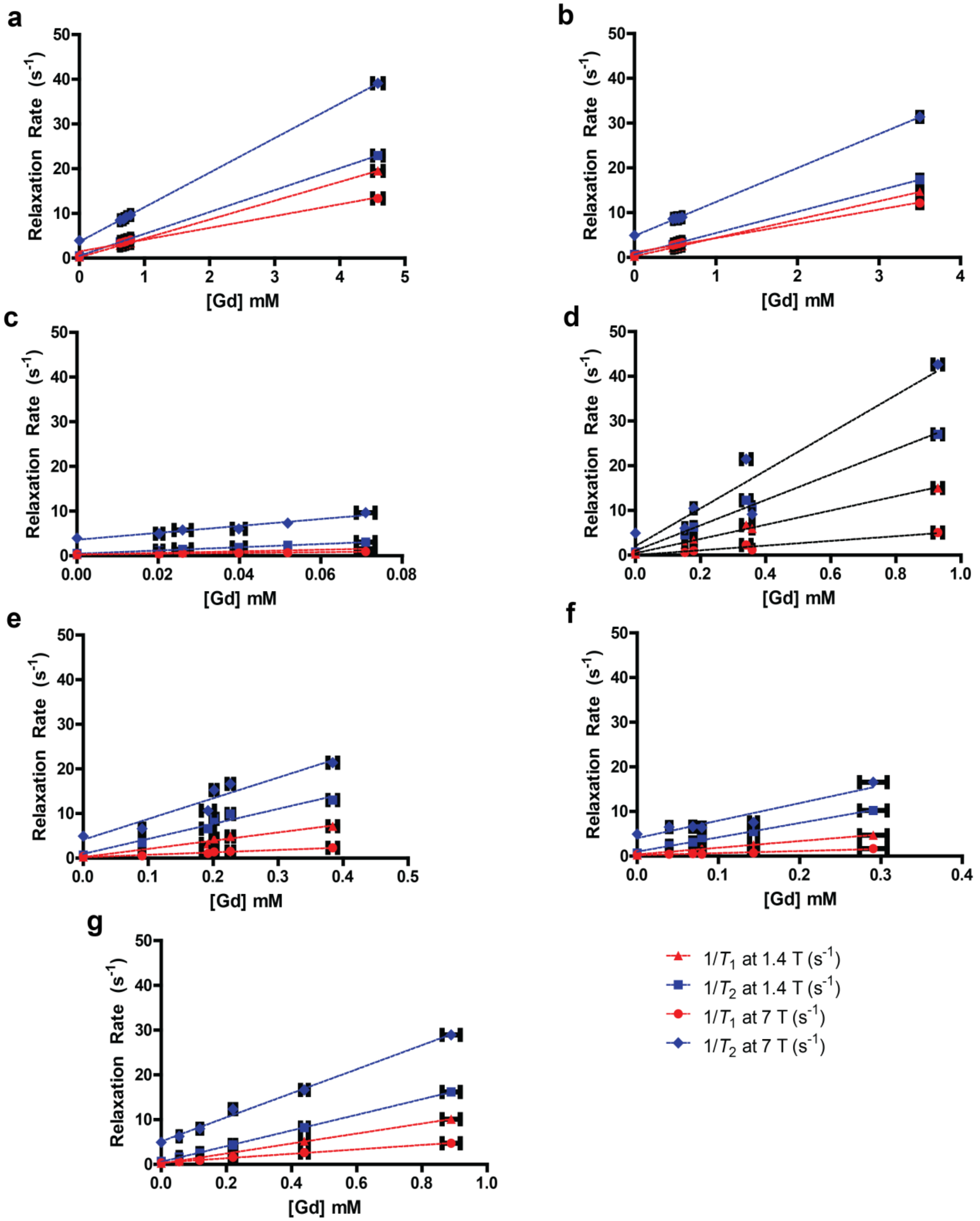

Figure S31. Longitudinal (r_1) and transverse (r_2) relaxivity measurements of Gd(III) complexes and Gd(III)-labeled protein cages. Longitudinal relaxation rates ($1/T_1$) at 3 T and 7 T. Transverse relaxation rates ($1/T_2$) at 3 T and 7 T. Relaxivity of **Gd-C4-IA** in (a) 50 mM sodium phosphate (pH = 8.0), 200 mM NaCl, 5 mM EDTA, and (b) 25 mM Tris (pH = 7.6), 200 mM NaCl, 5 mM EDTA. Relaxivity of (c) **Gd-AaLS-13** in 50 mM sodium phosphate (pH = 8.0), 200 mM NaCl, 5 mM EDTA. Relaxivity of (d) **Gd-OP-3$_{int}$C**, (e) **Gd-OP-2$_{int}$C**, (f) **Gd-OP-1$_{int}$C**, and (g) **Gd-OP-1$_{ext}$C** in 25 mM Tris (pH = 7.6), 200 mM NaCl, 5 mM EDTA.

Solution phantom images at 7 T for relaxivity

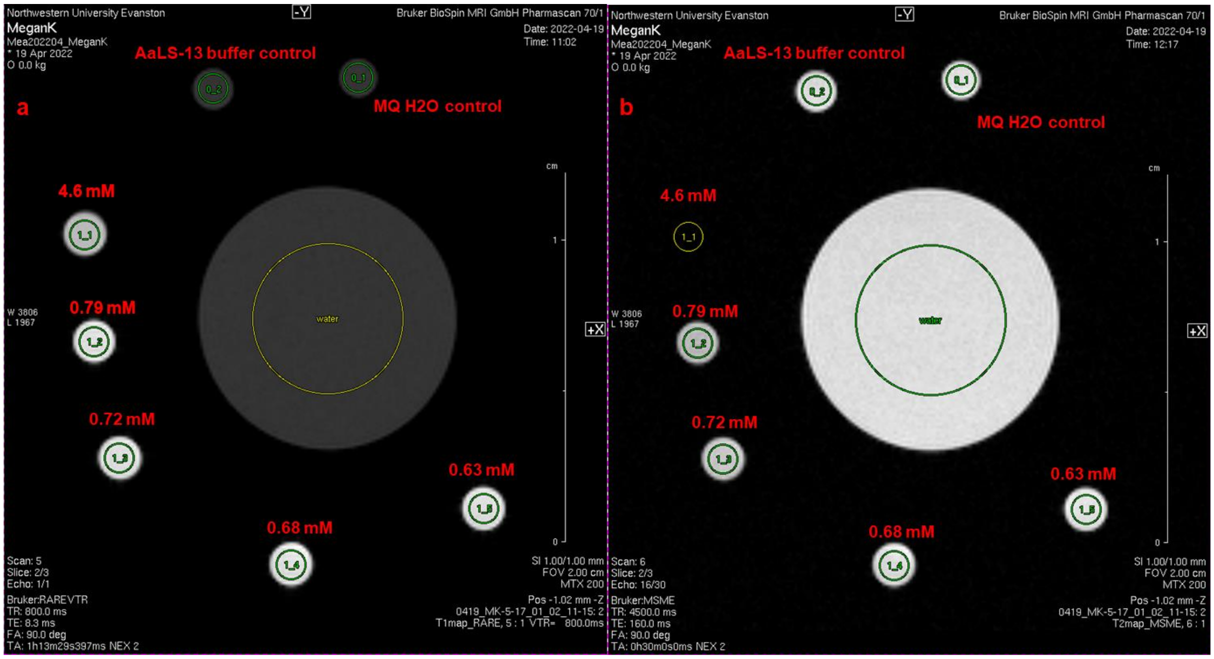

Figure S32. Solution phantom images of **Gd-C4-IA** in 50 mM sodium phosphate (pH 8.0), 200 mM NaCl, 5 mM EDTA at 7 T. (a) T_1 and (b) T_2 relaxation measurements.

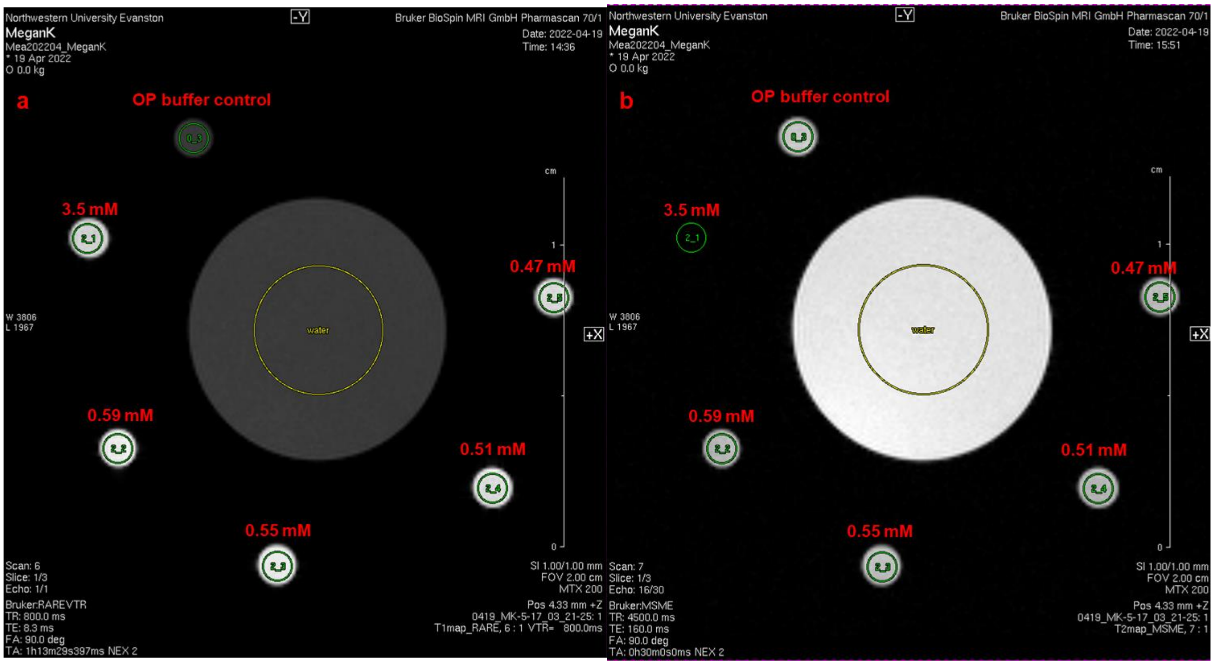

Figure S33. Solution phantom images of **Gd-C4-IA** in 25 mM Tris (pH 7.6), 200 mM NaCl, 5 mM EDTA at 7 T. (a) T_1 and (b) T_2 relaxation measurements.

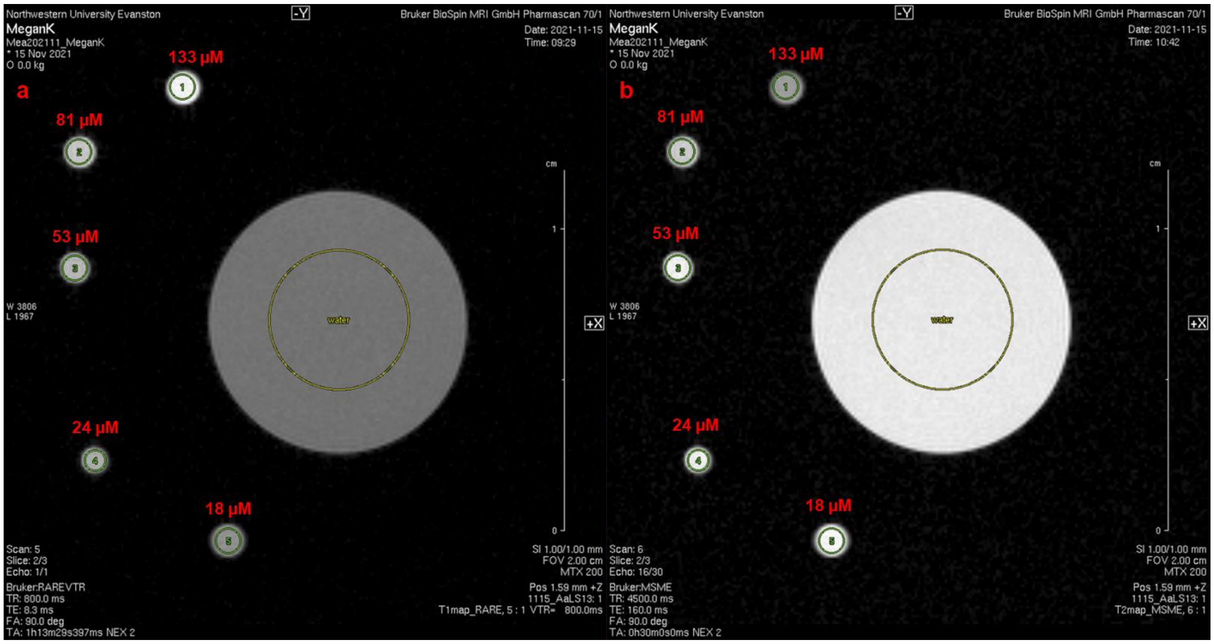

Figure S34. Solution phantom images of **Gd-AaLS-13** in 50 mM sodium phosphate (pH 8.0), 200 mM NaCl, 5 mM EDTA at 7 T. (a) T_1 and (b) T_2 relaxation measurements.

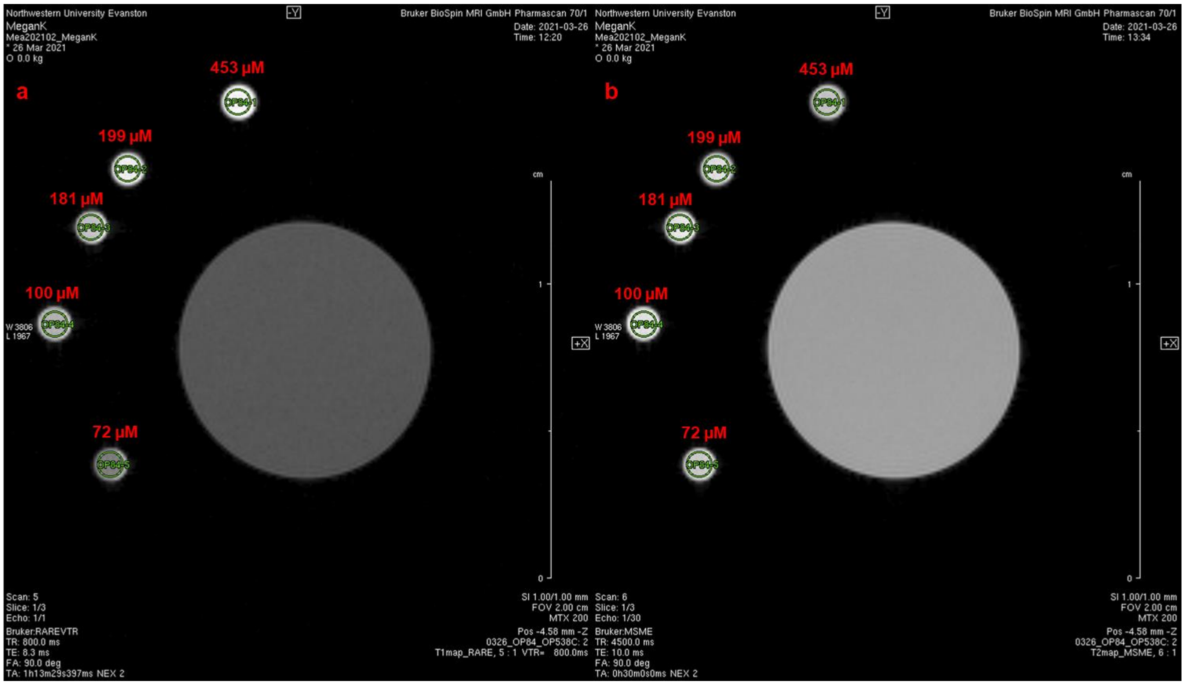

Figure S35. Solution phantom images of **Gd-OP-3$_{int}$C** in 25 mM Tris (pH 7.6), 200 mM NaCl, 5 mM EDTA at 7 T. (a) T_1 and (b) T_2 relaxation measurements.

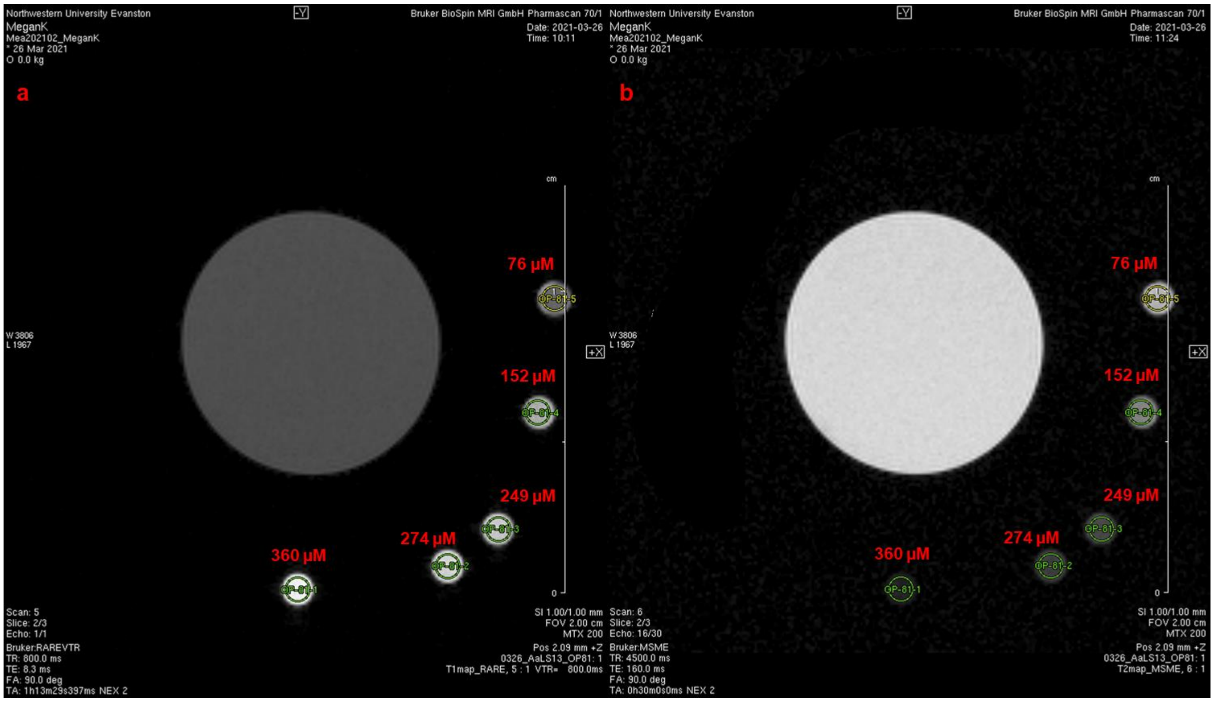

Figure S36. Solution phantom images of **Gd-OP-2$_{int}$C** in 25 mM Tris (pH 7.6), 200 mM NaCl, 5 mM EDTA at 7 T. (a) T_1 and (b) T_2 relaxation measurements.

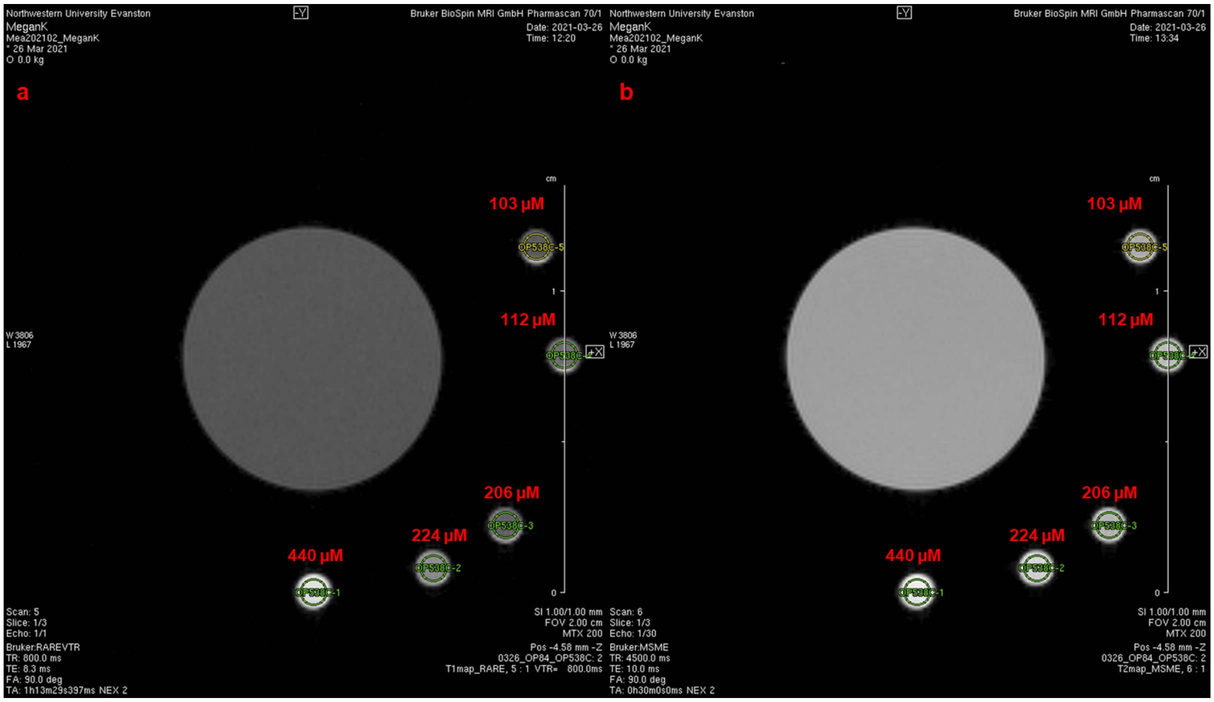

Figure S37. Solution phantom images of **Gd-OP-1$_{int}$C** in 25 mM Tris (pH 7.6), 200 mM NaCl, 5 mM EDTA at 7 T. (a) T_1 and (b) T_2 relaxation measurements.

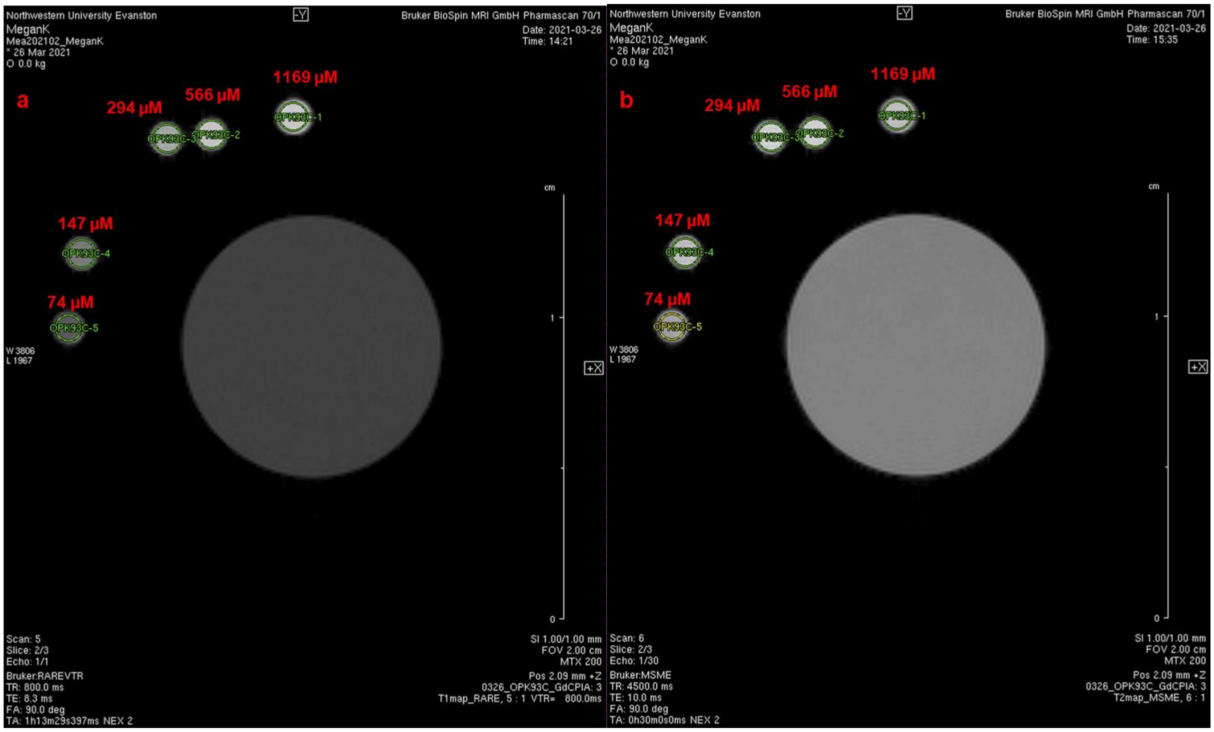

Figure S38. Solution phantom images of **Gd-OP-1$_{ext}$C** in 25 mM Tris (pH 7.6), 200 mM NaCl, 5 mM EDTA at 7 T. (a) T_1 and (b) T_2 relaxation measurements.

Determination of relaxivity values

The ionic relaxivity values ($r_{1,ionic}$ and $r_{2,ionic}$) obtained from the linear fit of relaxation rates versus concentration of Gd(III) at 1.4 T and 3 T using GraphPad Prism software. The reported error in ionic relaxivity measurements associated in each measurement was propagated through the linear regression analysis. Particle relaxivity values ($r_{1,particle}$ and $r_{2,particle}$) are calculated by multiplying the ionic relaxivity (per Gd relaxivity, $r_{1,ionic}$ and $r_{2,ionic}$) by the number of Gd(III) complexes per particle (**Eq. S1**).

$$r_{i,particle} = \left(r_{i,ionic}\right) \times (Avg\ Gd\ per\ cage);\ i = 1,2 \qquad \textbf{Eq. S1}$$

Table S2. Transverse Relaxivity Measurements

Sample Name	1.4 T at 37 °C		7 T at 25 °C	
	$r_{2,\text{ionic}}$ (mM^{-1} s^{-1})	$r_{2,\text{particle}}$ (mM^{-1} s^{-1})	$r_{2,\text{ionic}}$ (mM^{-1} s^{-1})	$r_{2,\text{particle}}$ (mM^{-1} s^{-1})
**Gd-C4-IA**[a]	4.90 ± 0.01	N/A	7.72 ± 0.05	N/A
**Gd-C4-IA**[b]	4.758 ± 0.008	N/A	7.58 ± 0.08	N/A
**Gd-AaLS-13**[a]	36.3 ± 0.9	5409	77 ± 9	11473
**Gd-OP-3$_{\text{int}}$C**[b]	28 ± 1	924	42 ± 7	1386
**Gd-OP-2$_{\text{int}}$C**[b]	34 ± 4	782	46 ± 7	1058
**Gd-OP-1$_{\text{int}}$C**[b]	32 ± 2	352	39 ± 6	429
**Gd-OP-1$_{\text{ext}}$C**[b]	17.5 ± 0.1	245	27 ± 1	378

Relaxation times (T_2) were measured with error of < 1%, while standard deviations of [Gd(III)] were determined by ICP-MS of triplicate samples.

[a]Relaxivity data in 50 mM sodium phosphate buffer (pH 8.0), 200 mM NaCl, 5 mM EDTA.

[b]Relaxivity data in 5 mM Tris (pH 7.6), 200 mM NaCl, 5 mM EDTA.

10. ^{1}H NMRD Measurements

^{1}H NMRD measurements of AaLS-13 and OP

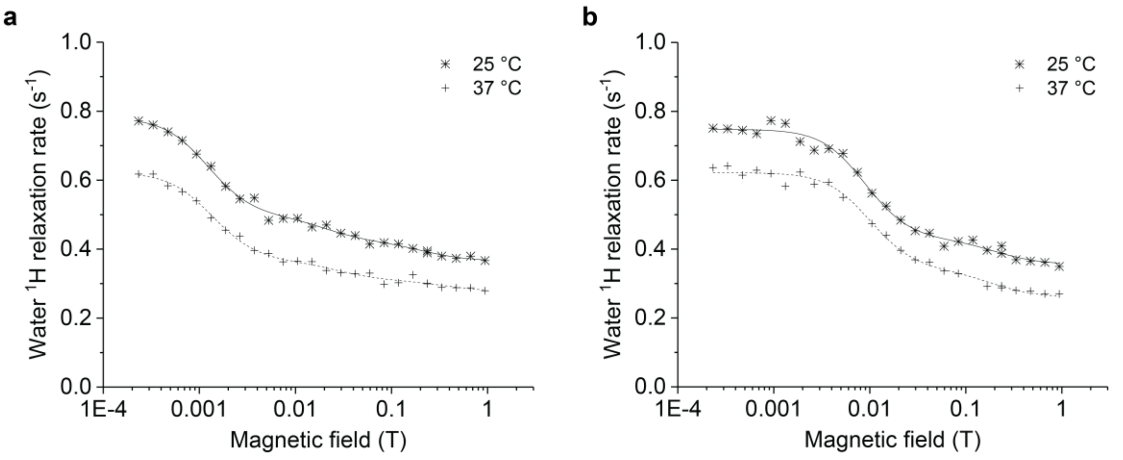

Figure S39. ^{1}H NMRD profiles of a water solution of unlabeled AaLS-13 and OP protein cages. Profiles of (a) **AaLS-13** and (b) **OP** at 0.222 and 0.442 mmol/dm^3 monomeric protein concentrations, respectively, at 25 and 37°C. The solid and dotted lines are the best fit profiles corresponding to the parameters reported in Table S3.

Proton Nuclear magnetic relaxation dispersion (^{1}H NMRD) profiles were acquired with a fast-field-cycling Stelar relaxometer. They provide the field dependence of the longitudinal relaxation rate of water protons in samples from 0.0002 to 1 T. Relaxation rates were measured with an error below 1%. Protein and Gd concentrations were 0.232 and 0.0979 mmol/dm^3, respectively, in the **Gd-AaLS-13** sample; 0.357 and 0.517 mmol/dm^3 in the **Gd-OP-3$_{int}$C** sample; 0.466 and 0.443 mmol/dm^3 in the **Gd-OP-2$_{int}$C** sample; 0.416 and 0.175 mmol/dm^3 in the **Gd-OP-1$_{int}$C** sample; 0.941 and 0.593 mmol/dm^3 in the **Gd-OP-1$_{ext}$C** sample. The relaxation profiles were calculated with **Eq. S2** and the best fit parameters are reported in Table S3 – S6.

$$R_1 = \alpha + \beta \sum_{i=1}^{3} c_i \left(\frac{0.2\,\tau_i}{1+\omega^2\tau_i^2} + \frac{0.8\,\tau_i}{1+4\omega^2\tau_i^2} \right) \qquad \textbf{Eq. S2}$$

**Table S3.** Diamagnetic Parameters from Best Fit Profiles				
Parameters	AaLS-13[a]		OP[b]	
	25 °C	37 °C	25 °C	37 °C
α (s^{-1})	0.37	0.28	0.36	0.26
β (s^{-2})	6.6×10^{6}		8.4×10^{6}	
c_1	0.03		0.16	
τ_1 (s)	1.7×10^{-6}	1.5×10^{-6}	2.3×10^{-7}	2.1×10^{-7}
c_2	0.11		0.84	
τ_2 (s)	9.9×10^{-8}	7.4×10^{-8}	1.0×10^{-8}	1.1×10^{-8}
τ_3 (s)	9.6×10^{-9}	5.6×10^{-9}	N/A	N/A
$^{*}c_3 = 1 - c_1 - c_2$				

Parameters from best fit profiles of Gd-C4-IA

**Table S4. Gd-C4-IA** Parameters from Best Fit Profiles				
Parameters	**Gd-C4-IA**[a]		**Gd-C4-IA**[b]	
	25 °C	37 °C	25 °C	37 °C
*r** (Å)	3.05			
*q**	1			
Δ_t (cm^{-1})	0.030		0.030	
τ_v (s)	17×10^{-12}	13×10^{-12}	17×10^{-12}	10×10^{-12}
τ_R (s)	86×10^{-12}	51×10^{-12}	77×10^{-12}	45×10^{-12}
τ_m (s)	$\approx10^{-7}$			

*fixed values.
[a]NMRD measurements in 50 mM sodium phosphate (pH 8.0), 200 mM NaCl, 5 mM EDTA
[b]NMRD measurements in 25 mM Tris (pH 7.6), 200 mM NaCl, 5 mM EDTA
The outer-sphere parameters *d* (distance of closest approach) and *D* (diffusion coefficient) were fixed to 3.6 Å and to 2.3×10^{-9} and 3.0×10^{-9} m^2/s at 25 and 37 °C, respectively.

Parameters from best fit profiles of Gd-labeled proteins

**Table S5. Gd-AaLS-13** Parameters from Best Fit Profiles		
Parameters	**Gd-AaLS-13**[a]	
	25 °C	37 °C
*r** (Å)	3.05	
*q**	1	
Δ_t (cm^{-1})	0.010	
τ_v (s)	32×10^{-12}	31×10^{-12}
τ_i (s)	5.2×10^{-9}	3.5×10^{-9}
τ_m (s)	1.3×10^{-7}	1.0×10^{-7}
S^2	0.28	
τ_l (s)	5.4×10^{-10}	2.5×10^{-10}
ZFS (cm^{-1})	0.013	
θ (°)	42	

*fixed values.

[a]NMRD measurements in 50 mM sodium phosphate (pH 8.0), 200 mM NaCl, 5 mM EDTA

[b]NMRD measurements in 25 mM Tris (pH 7.6), 200 mM NaCl, 5 mM EDTA

The outer-sphere parameters *d* (distance of closest approach) and *D* (diffusion coefficient) were fixed to 3.6 Å and to 2.3×10^{-9} and 3.0×10^{-9} m^2/s at 25 and 37 °C, respectively.

Parameters	Gd-OP-3$_{int}$C[b]		Gd-OP-2$_{int}$C[b]		Gd-OP-1$_{int}$C[b]		Gd-OP-1$_{ext}$C[b]	
	25 °C	37 °C	25 °C	37 °C	25 °C	37 °C	25 °C	37 °C
*r** (Å)	3.05		3.05		3.05		3.05	
*q**	1		1		1		1	
Δ_t (cm^{-1})	0.0095		0.0098		0.0097		0.0097	
τ_v (s)	28×10^{-12}	23×10^{-12}	32×10^{-12}	28×10^{-12}	28×10^{-12}	26×10^{-12}	28×10^{-12}	26×10^{-12}
τ_i (s)	3.7×10^{-9}	3.3×10^{-9}	4.0×10^{-9}	3.2×10^{-9}	4.3×10^{-9}	3.3×10^{-9}	1.8×10^{-9}	1.1×10^{-9}
τ_m (s)	1.0×10^{-7}	7.4×10^{-8}	1.0×10^{-7}	7.4×10^{-8}	1.0×10^{-7}	7.4×10^{-8}	1.0×10^{-7}	7.4×10^{-8}
S^2	0.46		0.39		0.36		0.36	
τ_l (s)	1.8×10^{-9}	4.8×10^{-10}	3.2×10^{-10}	9.8×10^{-11}	6.8×10^{-11}	3.8×10^{-11}	7.8×10^{-11}	2.8×10^{-11}
ZFS (cm^{-1})	0.020		0.018		0.020		0.018	
θ (°)	47		43		41		40	

Table S6. Gd-labeled OP Cages Parameters from Best Fit Profiles

*fixed values.

[a]NMRD measurements in 50 mM sodium phosphate (pH 8.0), 200 mM NaCl, 5 mM EDTA

[b]NMRD measurements in 25 mM Tris (pH 7.6), 200 mM NaCl, 5 mM EDTA

The outer-sphere parameters *d* (distance of closest approach) and *D* (diffusion coefficient) were fixed to 3.6 Å and to 2.3×10^{-9} and 3.0×10^{-9} m^2/s at 25 and 37 °C, respectively.

Best fit analysis of the profiles indicates that the parameters describing electron relaxation (Δ_t and τ_v) are very similar for all Gd-labeled protein cages. This finding shows that the electron relaxation mechanisms do not change significantly depending on the attachment point of the Gd(III) complex to the protein cages, as also suggested by the similar frequencies at which the relaxometry peaks are centered. A similar electron relaxation for **Gd-OP-3$_{int}$C**, **Gd-OP-2$_{int}$C** and **Gd-OP-1$_{int}$C** also confirms that magnetic coupling between different Gd ions is negligible even when multiple Gd-tags are attached to each protein monomer, because it would have the effect of decreasing the electron relaxation time. We note that the electron relaxation parameters are largely determined by the relaxivity at high fields (1 to 100 MHz), which is not influenced by the presence of static ZFS and can be analyzed with the SBM model, pointing out to the robustness of these parameters.

11. Solution Phantom Images

Solution phantom image sample preparation

Gd-AaLS-13 was studied in 50 mM sodium phosphate (pH 8.0), 200 mM NaCl, 5 mM EDTA, while **Gd-OP-3$_{int}$C** was studied in 25 mM Tris (pH 7.6), 200 mM NaCl, 5 mM EDTA. Samples were prepared at 90% target concentration in specific buffer and diluted with 10% FBS to achieve final concentration.

<u>Trial 1:</u> stock samples were prepared at 403 ± 2 µM **Gd-AaLS-13** and 351 ± 2 µM **Gd-OP-3$_{int}$C**. The stock samples were diluted to obtain 0.5 µM, 5 µM, 20 µM, and 67 µM for **Gd-AaLS-13**; and 0.5 µM, 5 µM, 20 µM, and 200 µM for **Gd-OP-3$_{int}$C**. A 1.5 mL aliquot of each solution was pipetted into a protein low-bind Eppendorf tube. Control samples were prepared at 10% FBS with the appropriate buffer. These Eppendorf tubes were imaged using a Bruker PharmaScan 7 T MR imaging spectrometer

<u>Trial 2:</u> stock samples were prepared at 279.1 ± 0.8 µM **Gd-AaLS-13** and 177 ± 2 µM **Gd-OP-3$_{int}$C**. These stock samples were diluted to obtain 67 µM **Gd-AaLS-13**; and 12.5 µM and 20 µM **Gd-OP-3$_{int}$C**. Control samples were prepared at 10% FBS with the appropriate buffer. A 60 µL aliquot of each sample was pipetted into flame sealed Pasteur pipettes. The pipette tips containing solution were scored, separated, and sealed with parafilm to make small capillaries containing solution. These capillaries were imaged using a Bruker PharmaScan 7 T MR imaging spectrometer and Bruker BioSpec 9.4 T MR imaging spectrometer. A 1.5 mL aliquot of each sample was pipetted into a protein low-bind Eppendorf tube. These Eppendorf tubes were imaged using a Siemens Prisma 3 T MR imaging spectrometer.

Phantom image measurements at 3 T

The Eppendorf tubes from experiment 2 were imaged using a Siemens 3 T Prisma MR imaging spectrometer. T_1 relaxation times were measured using a dual gradient echo method (StaGE) with two different flip angles. T_1 analysis was carried out using the image sequence analysis tool in Paravision 6.0 software (Bruker) to selected ROIs for each axial slice.

Phantom image measurements at 7 T

These eppendorfs were imaged using a Bruker PharmaScan 7 T MR imaging spectrometer (Bruker BioSpin, Billerca, MA, USA). T_1 relaxation times were measured using a rapid-acquisition rapid-echo (RARE-VTR) T1-map pulse sequence with static TE (10 ms) and variable TR (100, 200, 400, 500, 750, 1000, 2500, 7500, 10000 ms) values. Imaging parameters were as follows: field of view, 25 x 25 mm^2; matrix size, 256 x 256; number of axial slices, 5; slice thickness, 1.0 mm; and averages, 4. T_2 relaxation times were measured using a multislice multiecho (MSME)

T_2-map pulse sequence, with static TR (5000 ms) and 32 fitted echoes in 11 ms intervals (11, 22, ..., 352 ms). Imaging parameters were as follows: field of view, 25 × 25 mm; matrix size, 256 × 256; number of axial slices, 4; slice thickness, 1.0 mm; and averages, 3. T_1 and T_2 analysis was carried out using the image sequence analysis tool in Paravision 6.0 software (Bruker) with mono-exponential curve-fitting of image intensities of selected ROIs for each axial slice.

Phantom image measurements at 9.4 T

These eppendorfs were imaged using a Bruker PharmaScan 7 T MR imaging spectrometer (Bruker BioSpin, Billerca, MA, USA). T_1 relaxation times were measured using a rapid-acquisition rapid-echo (RARE-VTR) T_1-map pulse sequence with static TE (10 ms) and variable TR (100, 200, 400, 500, 750, 1000, 2500, 7500, 10000 ms) values. Imaging parameters were as follows: field of view, 25 x 25 mm^2; matrix size, 256 x 256; number of axial slices, 5; slice thickness, 1.0 mm; and averages, 4. T_2 relaxation times were measured using a multislice multiecho (MSME) T_2-map pulse sequence, with static TR (5000 ms) and 32 fitted echoes in 11 ms intervals (11, 22, ..., 352 ms). Imaging parameters were as follows: field of view, 25 × 25 mm; matrix size, 256 × 256; number of axial slices, 4; slice thickness, 1.0 mm; and averages, 3. T_1 and T_2 analysis was carried out using the image sequence analysis tool in Paravision 6.0 software (Bruker) with mono-exponential curve-fitting of image intensities of selected ROIs for each axial slice.

Equation for ΔR₁

Percent change in relaxation rate is determined by **Eq. S3**.[6]

$$\%\Delta \boldsymbol{R_1} = \frac{(\boldsymbol{R_{1,sample}} - \boldsymbol{R_{1,control}})}{\boldsymbol{R_{1,control}}} \cdot \mathbf{100}\% \qquad \textbf{Eq. S3}$$

Phantom image data

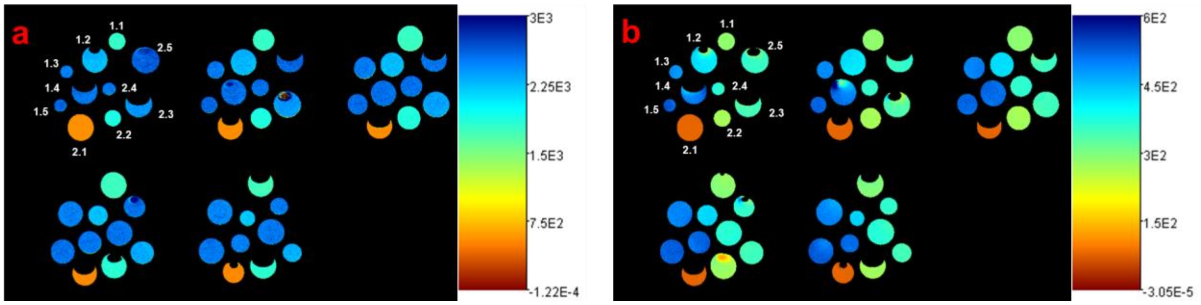

Figure S40. Trial 1 (a) T_1 and (b) T_2 serum phantom images of **Gd-AaLS-13** and **Gd-OP-3$_{int}$C** at 7 T. Imaged samples are (1.1) 67 µM **Gd-AaLS-13** in 10% FBS in 50 mM sodium phosphate (pH 8.0), 200 mM NaCl, 5 mM EDTA; (1.2) 20 µM **Gd-AaLS-13** in 10% FBS in 50 mM sodium phosphate (pH 8.0), 200 mM NaCl, 5 mM EDTA; (1.3) 5 µM **Gd-AaLS-13** in 10% FBS in 50 mM sodium phosphate (pH 8.0), 200 mM NaCl, 5 mM EDTA; (1.4) 0.5 µM **Gd-AaLS-13** in 10% FBS in 50 mM sodium phosphate (pH 8.0), 200 mM NaCl, 5 mM EDTA; (1.5) 10% FBS in 50 mM sodium phosphate (pH 8.0), 200 mM NaCl, 5 mM EDTA; (2.1) 200 µM **Gd-OP-3$_{int}$C** in 10% FBS in 25 mM Tris (pH 7.6), 200 mM NaCl, 5 mM EDTA; (2.2) 20 µM **Gd-OP-3$_{int}$C** in 10% FBS in 25 mM Tris (pH 7.6), 200 mM NaCl, 5 mM EDTA; (2.3) 5 µM **Gd-OP-3$_{int}$C** in 10% FBS in 25 mM Tris (pH 7.6), 200 mM NaCl, 5 mM EDTA; (2.4) 0.5 µM **Gd-OP-3$_{int}$C** in 10% FBS in 25 mM Tris (pH 7.6), 200 mM NaCl, 5 mM EDTA; and (2.5) 10% FBS in 25 mM Tris (pH 7.6), 200 mM NaCl, 5 mM EDTA.

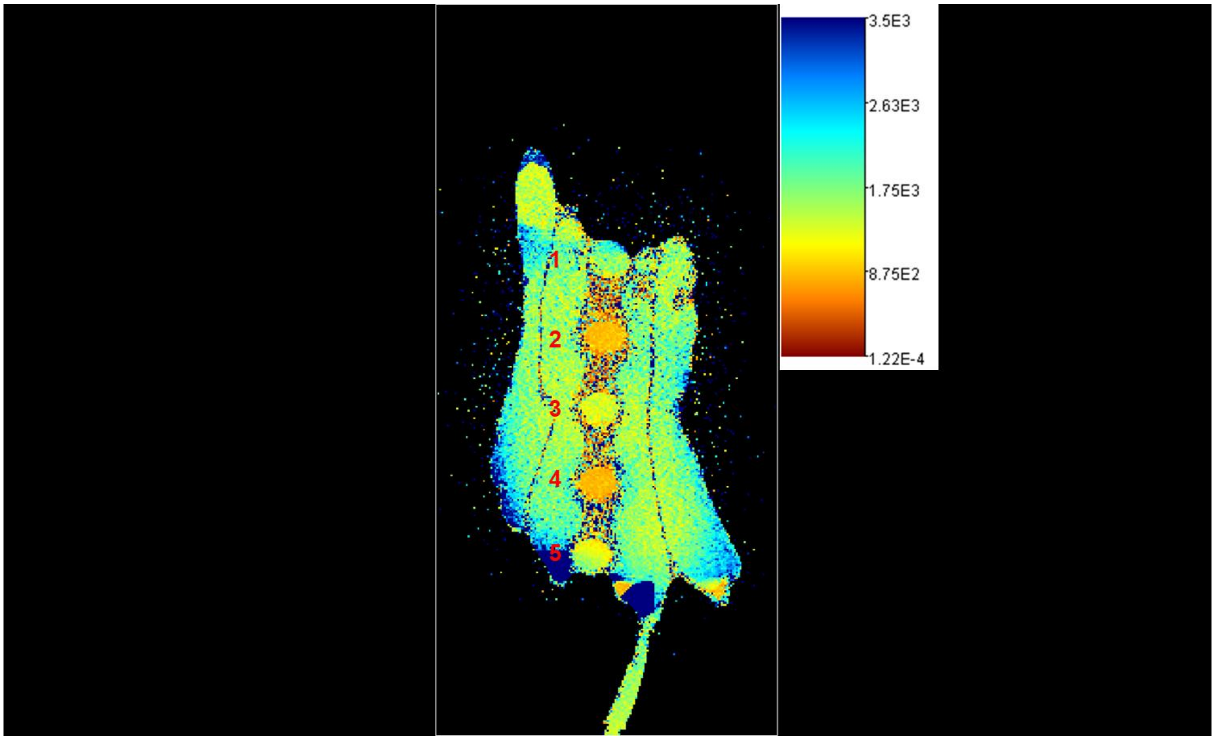

Figure S41. Trial 2 T_1 serum phantom image of **Gd-AaLS-13** and **Gd-OP-3$_{int}$C** at 3 T. Imaged samples are (1) 10% FBS in 50 mM sodium phosphate (pH 8.0), 200 mM NaCl, 5 mM EDTA; (2) 67 µM **Gd-AaLS-13** in 10% FBS in 50 mM sodium phosphate (pH 8.0), 200 mM NaCl, 5 mM EDTA; (3) 10% FBS in 25 mM Tris (pH 7.6), 200 mM NaCl, 5 mM EDTA; (4) 20 µM **Gd-OP-3$_{int}$C** in 10% FBS in 25 mM Tris (pH 7.6), 200 mM NaCl, 5 mM EDTA; and (5) 12.5 µM **Gd-OP-3$_{int}$C** in 10% FBS in 25 mM Tris (pH 7.6), 200 mM NaCl, 5 mM EDTA.

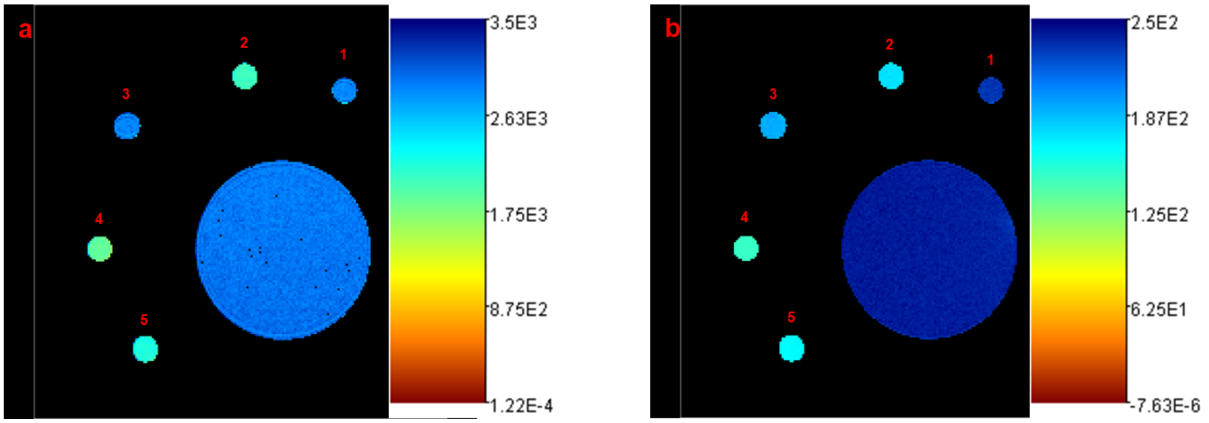

Figure S42. Trial 2 (a) T_1 and (b) T_2 serum phantom images of **Gd-AaLS-13** and **Gd-OP-3$_{int}$C** at 7 T. Imaged samples are (1) 10% FBS in 50 mM sodium phosphate (pH 8.0), 200 mM NaCl, 5 mM EDTA; (2) 67 µM **Gd-AaLS-13** in 10% FBS in 50 mM sodium phosphate (pH 8.0), 200 mM NaCl, 5 mM EDTA; (3) 10% FBS in 25 mM Tris (pH 7.6), 200 mM NaCl, 5 mM EDTA; (4) 20 µM **Gd-OP-3$_{int}$C** in 10% FBS in 25 mM Tris (pH 7.6), 200 mM NaCl, 5 mM EDTA; and (5) 12.5 µM **Gd-OP-3$_{int}$C** in 10% FBS in 25 mM Tris (pH 7.6), 200 mM NaCl, 5 mM EDTA.

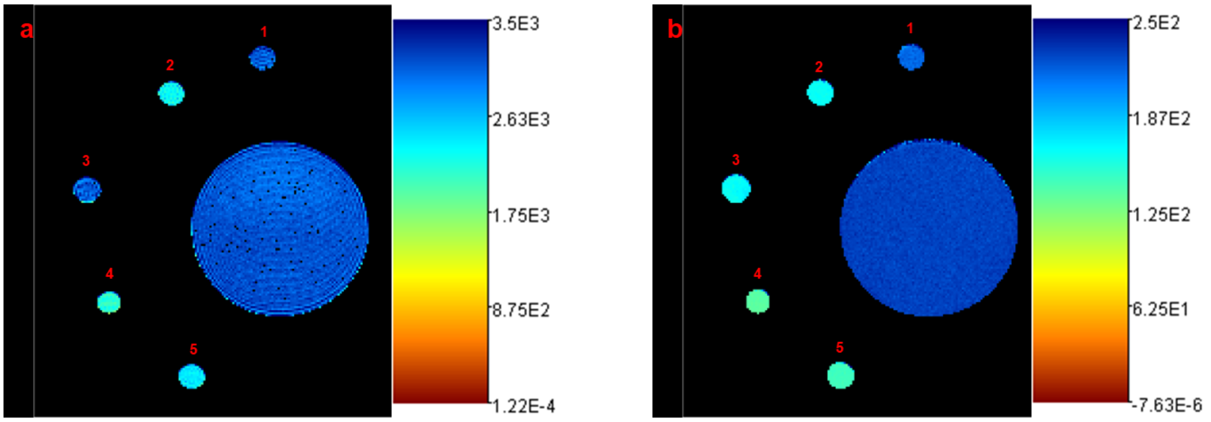

Figure S43. Trial 2 (a) T_1 and (b) T_2 serum phantom images of **Gd-AaLS-13** and **Gd-OP-3$_{int}$C** at 9.4 T. Imaged samples are (1) 10% FBS in 50 mM sodium phosphate (pH 8.0), 200 mM NaCl, 5 mM EDTA; (2) 67 µM **Gd-AaLS-13** in 10% FBS in 50 mM sodium phosphate (pH 8.0), 200 mM NaCl, 5 mM EDTA; (3) 10% FBS in 25 mM Tris (pH 7.6), 200 mM NaCl, 5 mM EDTA; (4) 20 µM **Gd-OP-3$_{int}$C** in 10% FBS in 25 mM Tris (pH 7.6), 200 mM NaCl, 5 mM EDTA; and (5) 12.5 µM **Gd-OP-3$_{int}$C** in 10% FBS in 25 mM Tris (pH 7.6), 200 mM NaCl, 5 mM EDTA.

Phantom image analysis

Determined contrast enhancement as percent change in relaxation rate (**Eq. S3**) with serum phantom image data from Trial 1 and Trial 2 T_1 and T_2 measurements (**Figures S40**-**S43**).

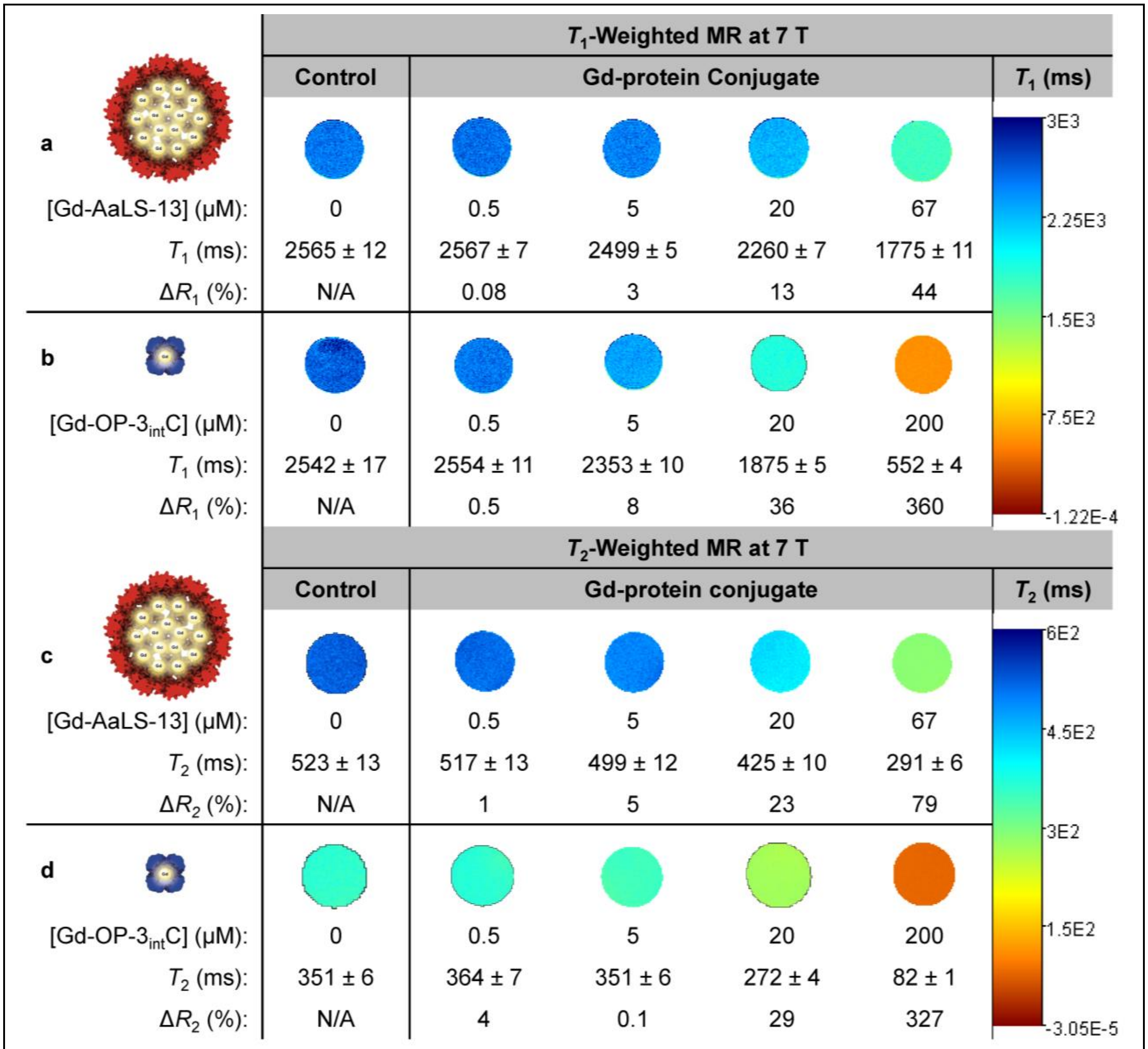

Figure S44. Trial 1 (a, b) T_1-weighted and (c, d) T_2-weighted MR solution phantom images of (a, c) **Gd-AaLS-13** and (b, d) **Gd-OP-3$_{int}$C** at 7 T. (a) and (c) Control sample of 10% FBS in 50 mM sodium phosphate (pH 8.0), 200 mM NaCl, 5 mM EDTA. **Gd-AaLS-13** samples were prepared at 0.5 µM, 5 µM, 20 µM, and 67 µM monomer. (b) and (d) Control sample of 10% FBS in 25 mM Tris (pH 7.6), 200 mM NaCl, 5 mM EDTA. **Gd-OP-3$_{int}$C** samples were prepared at 0.5 µM, 5 µM, 20 µM, 200 µM monomer.

At 7 T, 20 µM **Gd-AaLS-13** with respect to monomer increased R_1 by 13%, while 67 µM **Gd-AaLS-13** with respect to monomer increased R_1 by 44%. However, 20 µM **Gd-OP-3$_{int}$C** with respect to monomer increased R_1 by 36%, while 200 µM **Gd-OP-3$_{int}$C** with respect to monomer increased R_1 by 360%. The T_2-weighted MR images also show relaxation enhancement at the same concentrations. 20 µM **Gd-AaLS-13** with respect to monomer increased R_2 by 23%, while

67 µM **Gd-AaLS-13** with respect to monomer increased R_2 by 79%. However, 20 µM **Gd-OP-3$_{int}$C** with respect to monomer increased R_2 by 29%, while 200 µM **Gd-OP-3$_{int}$C** with respect to monomer increased R_2 by 327%.

Figure S45 includes 12.5 µM **Gd-OP-3$_{int}$C** with respect to monomer data, which increased R_1 by 4% at 3 T, 32% at 7 T, and 27% at 9.4 T.

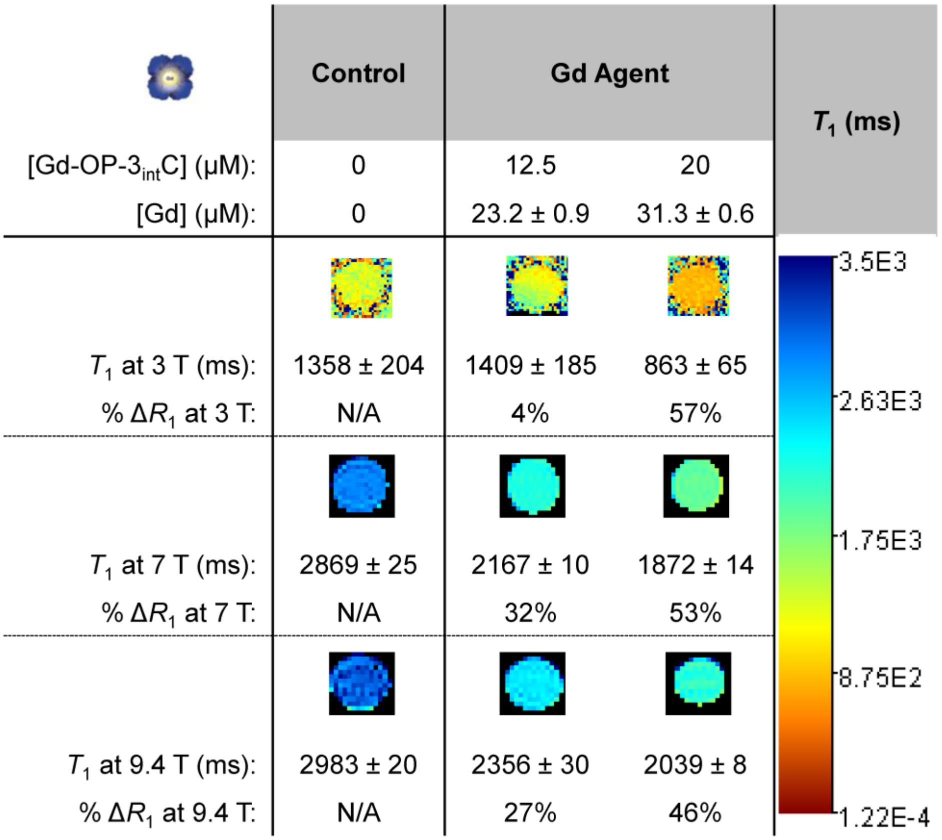

	Control	Gd Agent	
[Gd-OP-3$_{int}$C] (µM):	0	12.5	20
[Gd] (µM):	0	23.2 ± 0.9	31.3 ± 0.6
T_1 at 3 T (ms):	1358 ± 204	1409 ± 185	863 ± 65
% ΔR_1 at 3 T:	N/A	4%	57%
T_1 at 7 T (ms):	2869 ± 25	2167 ± 10	1872 ± 14
% ΔR_1 at 7 T:	N/A	32%	53%
T_1 at 9.4 T (ms):	2983 ± 20	2356 ± 30	2039 ± 8
% ΔR_1 at 9.4 T:	N/A	27%	46%

Figure S45. Trial 2 T_1-weighted MR solution phantom images of **Gd-OP-3$_{int}$C** at 3 T, 7 T, and 9.4 T. Control sample of 10% FBS in 25 mM Tris (pH 7.6), 200 mM NaCl, 5 mM EDTA; while **Gd-OP-3$_{int}$C** samples were prepared at 12.5 µM and 20 µM with respect to monomer.

12. References

(1) Chanthamath, S.; Takaki, S.; Shibatomi, K.; Iwasa, S. Highly stereoselective cyclopropanation of alpha,beta-unsaturated carbonyl compounds with methyl (diazoacetoxy)acetate catalyzed by a chiral ruthenium(II) complex. *Angew Chem Int Ed Engl* **2013**, *52* (22), 5818-5821. DOI: 10.1002/anie.201300468 From NLM Medline.

(2) Prasuhn, D. E., Jr.; Yeh, R. M.; Obenaus, A.; Manchester, M.; Finn, M. G. Viral MRI contrast agents: coordination of Gd by native virions and attachment of Gd complexes by azide-alkyne cycloaddition. *Chem Commun (Camb)* **2007**, (12), 1269-1271. DOI: 10.1039/b615084e From NLM Medline.

(3) Machitani, K.; Sakamoto, H.; Nakahara, Y.; Kimura, K. Molecular design of tetraazamacrocyclic derivatives bearing a spirobenzopyran and three carboxymethyl moeities and their metal-ion complexing behavior. *Anal. Sci* **2008**, *24*, 463-469.

(4) Edwardson, T. G. W.; Tetter, S.; Hilvert, D. Two-tier supramolecular encapsulation of small molecules in a protein cage. *Nat Commun* **2020**, *11* (1), 5410. DOI: 10.1038/s41467-020-19112-1 From NLM Medline.

(5) Edwardson, T. G. W.; Mori, T.; Hilvert, D. Rational Engineering of a Designed Protein Cage for siRNA Delivery. *J Am Chem Soc* **2018**, *140* (33), 10439-10442. DOI: 10.1021/jacs.8b06442 From NLM Medline.

(6) Elster, A. D. Field-Strength Dependence of Gadolinium Enhancement - Theory and Implications. *American Journal of Neuroradiology* **1994**, *15* (8), 1420-1423.
